# High-resolution adaptive optical imaging within thick scattering media using closed-loop accumulation of single scattering

**DOI:** 10.1038/s41467-017-02117-8

**Published:** 2017-12-18

**Authors:** Sungsam Kang, Pilsung Kang, Seungwon Jeong, Yongwoo Kwon, Taeseok D. Yang, Jin Hee Hong, Moonseok Kim, Kyung–Deok Song, Jin Hyoung Park, Jun Ho Lee, Myoung Joon Kim, Ki Hean Kim, Wonshik Choi

**Affiliations:** 1 Center for Molecular Spectroscopy and Dynamics, Institute for Basic Science (IBS), Seoul, 02841 Korea; 2 Department of Physics, Korea University, Seoul, 02841 Korea; 3 Department of Ophthalmology and University of Ulsan College of Medicine, Asan Medical Center, Seoul, 05505 Korea; 4 Division of Integrative Biosciences & Biotechnology, Department of Mechanical Engineering, Pohang University of Science and Technology, Pohang, 37673 Korea

## Abstract

Thick biological tissues give rise to not only the multiple scattering of incoming light waves, but also the aberrations of remaining signal waves. The challenge for existing optical microscopy methods to overcome both problems simultaneously has limited sub-micron spatial resolution imaging to shallow depths. Here we present an optical coherence imaging method that can identify aberrations of waves incident to and reflected from the samples separately, and eliminate such aberrations even in the presence of multiple light scattering. The proposed method records the time-gated complex-field maps of backscattered waves over various illumination channels, and performs a closed-loop optimization of signal waves for both forward and phase-conjugation processes. We demonstrated the enhancement of the Strehl ratio by more than 500 times, an order of magnitude or more improvement over conventional adaptive optics, and achieved a spatial resolution of 600 nm up to an imaging depth of seven scattering mean free paths.

## Abstract (web summary)

Optical imaging deep in biological tissue is difficult due to multiple scattering and specimen induced aberrations of both the incident and reflected light. Here, Kang et al. develop an adaptive closed-loop algorithm to correct tissue aberrations in the presence of multiple scattering for deep tissue imaging.

## Introduction

Reaching diffraction-limited spatial resolution has been a challenging task for optically imaging targets embedded deep within scattering media, such as biological tissues^1^. Multiple scattering events attenuate light waves that preserve the original incidence momenta and generate multiply scattered waves that act as strong background noise. As the target depth is increased, these combined effects lead to the exponential decrease of the signal-to-noise ratio (SNR). Consequently, sub-micron-scale biological reactions occurring inside living tissues have been optically inaccessible, limiting the effectiveness of optical microscopy in the investigation of the early stages of disease progression and the studies of various biological microenvironments such as nervous systems.

When considering a target spatial resolution close to the diffraction limit, the attenuation of the SNR by multiple light scattering is not the only challenge. In fact, the so-called specimen-induced aberration is an equally important issue to address^2^. The signal waves that preserve original incidence momenta are not only attenuated in their intensity by the multiple light scattering, but their phases are also retarded due to the heterogeneity of the medium. These phase retardations vary depending on the propagation angle^3^, which broadens the width of the point spread function and reduces its peak height. Consequently, the spatial resolving power is degraded and the SNR is further reduced in addition to the reduction caused by multiple light scattering. These detrimental effects of the specimen-induced aberration are much more pronounced for high-resolution imaging as waves propagating at large incidence angles, which retain the high spatial frequency information, travel long paths and are more likely to experience large phase retardations.

Numerous previous studies have tried to deal with scattering and aberrations for deep-tissue imaging. The methods for dealing with scattering have used temporal and/or confocal gating for the selective detection of signal waves. Examples include optical coherence microscopy (OCM) and multi-photon microscopy^4–9^. We also reported a method termed collective accumulation of single scattering (CASS) microscopy^10^. CASS microscopy uses both time-gated detection and spatial input–output correlation to preferentially accumulate single-scattered waves, which are the waves scattered only once by the target object and not at all by the medium. But the specimen-induced aberration easily undermines these gating operations. Using eigenchannels to better accumulate signal waves has been attempted, but this does not guarantee aberration compensation^11,12^.

Various methods have been proposed to deal with aberrations in the context of adaptive optics (AO)^13,14^. A straightforward approach has been to use the intensity or the sharpness of the acquired images as indirect measures of the degree of aberrations and to maximize them by iteratively shaping the wavefront of incident waves^15–19^. In most cases, control of orthogonal modes, such as Zernike polynomials, was used to deal with slowly varying aberrations in the pupil plane^20^. These so-called sensorless approaches usually require many measurements for the experimental feedback control, and fluorescent molecules can be bleached during the process. However, some approaches employed pixel-based wavefront-shaping devices to compensate very steep phase gradients and high-order modes of the aberrations^15,17^, and introduced parallelization schemes to minimize the number of measurements^19^.

The other approach is to identify the aberrations by the wavefront sensing of backscattered waves using Shack–Hartmann wavefront sensors^21^ or coherence-gated wavefront sensing^22,23^. Especially when there exist point particles called guide stars nearby the objects of interest, recording the wavefront of the backscattered waves from the point particles is a direct measure of aberrations^24^. This wavefront sensing AO can be ideal since aberrations can be determined by only a few measurements even up to high-order modes. However, the need for guide stars is a stringent condition for most biological applications. To alleviate this requirement, one can either assume that the target object is mostly point particles^22^ or the aberration has translational memory effects^25^, but the assumptions may not work for thick biological tissues.

Even if the existing aberration correction methods mentioned above have shortcomings, they tend to work much better for fluorescence imaging than reflectance imaging. In the case of fluorescence imaging, the aberrations in the illumination and imaging paths are rather independent, and their distinction is clear as emission wavelengths are different from those of the excitation. Therefore, it is possible to separately address the aberrations in the illumination and imaging paths. In the case of reflectance imaging, however, incident and backscattered waves have the same wavelength, and yet they are convolved in forming an object image. For these reasons, it is extremely difficult to separate out one-way aberration necessary for the aberration compensation unless there exist guide stars. Moreover, the existence of strong multiple light scattering having the same wavelength as signal waves makes the problem even worse. Indeed, there has been almost no successful implementation of adaptive optics for high-resolution reflectance imaging although this imaging modality is readily applicable to in vivo biomedical imaging due to its label-free imaging capability.

In this article, we present a label-free and high-resolution imaging method that can identify sample-induced aberrations in illumination and imaging paths separately without guide stars and even in the presence of multiple light scattering. We used a time-gated optical coherence imaging to record the amplitude and phase maps of backscattered waves from the specimens for various illumination angles. In the image reconstruction process, we introduced separate angle-dependent phase factors for the incident and reflected waves, and identified phase corrections that preferentially accumulate single-scattered waves over multiple-scattered ones for the forward and phase-conjugation processes. By applying these angle-dependent phase corrections to the initial data, we could not only optimize the accumulation of single-scattering signals but also significantly reduce the effect of image distortion. Using this method, which we term ‘closed-loop accumulation of single scattering’ (CLASS) microscopy, we achieved a spatial resolution of 600 nm up to the imaging depth of seven scattering mean free paths. To demonstrate the applicability of CLASS microscopy to biological tissues, we conducted imaging of a rabbit’s cornea infected by the *Aspergillus fumigatus*, a type of fungi, and successfully visualized individual fungal filaments embedded within opaque fungal infection.

## Results

### The effects of sample-induced aberrations

**Fig. 1:**
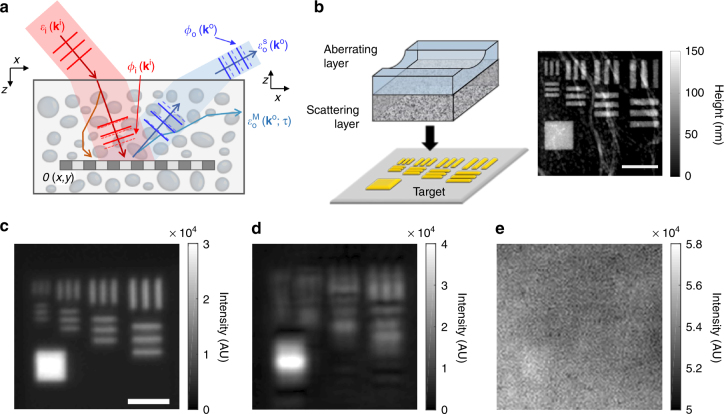
The effect of scattering and sample-induced aberrations in the reflectance imaging. **a** Description of sample-induced aberrations in the illumination and imaging paths. The phase of the unscattered component of an incident wave with transverse wavevector ${\vec{\bf k}}^{\mathrm{i}}$ is retarded by $\phi _{\mathrm{i}}\left( {{\vec{\bf k}}^{\mathrm{i}}} \right)$, and that of the reflected wave from the target object is retarded by $\phi _{\mathrm{o}}\left( {{\vec{\bf k}}^{\mathrm{o}}} \right)$. ${\cal E}_{\mathrm{o}}^{\mathrm{S}}\left( {{\vec{\bf k}}^{\mathrm{o}}} \right)$ and ${\cal E}_{\mathrm{o}}^{\mathrm{M}}\left( {{\vec{\bf k}}^{\mathrm{o}}} \right)$ stand for single- and multiple-scattered waves. O(*x*,*y*): amplitude reflectance of a target object. **b** Layout of the phantom sample. An asymmetric aberrating layer made of a clean PDMS block with a cylindrical groove was placed on the top of a 7*l*_s_-thick scattering layer. A resolution target was placed underneath the scattering layer. The gray scale image is the topography of the target measured by atomic force microscopy. Scale bar, 4 μm. Color map, height in nanometers. **c** Incoherent image of the target without the scattering and aberrating layers. The image was recorded in the reflection geometry, and light emitting diode (*λ* = 780nm) was used as a light source. Scale bar, 4 µm. **d**, **e** Same as (**c**), but with an aberrating layer and with both the scattering and aberrating layers, respectively. Color bars in (**c**–**e**), intensity in arbitrary unit

Let us consider a plane wave, $E\left( {x,y,z = 0;{\vec{\mathbf k}}^{\mathrm{i}}} \right) = {\mathrm{exp}}\left[ { - ik_{\mathrm{x}}^{\mathrm{i}}x - ik_{\mathrm{y}}^{\mathrm{i}}y} \right]$, incident (superscript i) to a target object embedded in a thick scattering medium, where ${\vec{\mathbf k}}^{\mathrm{i}} = \left( {k_{\mathrm{x}}^{\mathrm{i}},k_{\mathrm{y}}^{\mathrm{i}}} \right)$ is the transverse wavevector of the incident wave (Fig. 1a). When this wave travels through the scattering medium of thickness *L*, the intensity of the wave that preserves its original momentum is attenuated by a factor of exp(−*L*/*l*_s_), where *l*_s_ is the scattering mean free path, due to multiple light scattering. Moreover, this unscattered wave undergoes the phase retardation $\phi _{\mathrm{i}}\left( {{\vec{\mathbf k}}^{\mathrm{i}}} \right)$ depending on ${\vec{\mathbf k}}^{\mathrm{i}}$. Subsequently, it is reflected by the target object whose amplitude reflectance can be described by the object function O(*x,y*), and gains the transverse wavevector ${\mathrm{\Delta }}{\vec{\mathbf k}}$ driven by the object spectrum ${\cal O}\left( {{\mathrm{\Delta }}{\vec{\mathbf k}}} \right)$, which is the Fourier transform of the object function. On its way out (superscript o), the wave that now has the wavevector of ${\vec{\mathbf k}}^{\mathrm{o}} = {\vec{\mathbf k}}^{\mathrm{i}} + {\mathrm{\Delta }}{\vec{\mathbf k}}$ is again attenuated by the multiple scattering process and also experiences the additional aberration described by the angle-dependent phase retardation $\phi _{\mathrm{o}}\left( {{\vec{\mathbf k}}^{\mathrm{o}}} \right)$. Therefore, the angular spectrum of the reflected wave that has the flight time of *τ*_0_ = 2*L/c* is written as

1 $${\cal E}\left( {{\vec{\mathbf k}}^{\mathrm{o}};{\vec{\mathbf k}}^{\mathrm{i}},\tau _0} \right) 	= \sqrt \gamma P_{\mathrm{o}}^{\mathrm{a}}\left( {{\vec{\mathbf k}}^{\mathrm{i}} + {\mathrm{\Delta }}{\vec{\mathbf k}}} \right){\cal O}\left( {{\mathrm{\Delta }}{\vec{\mathbf k}}} \right)P_{\mathrm{i}}^{\mathrm{a}}\left( {{\vec{\mathbf k}}^{\mathrm{i}}} \right) \\ 	+ \sqrt \beta {\cal E}_{\mathrm{o}}^{\mathrm{M}}\left( {{\vec{\mathbf k}}^{\mathrm{i}} + {\mathrm{\Delta }}{\vec{\mathbf k}};\tau _0} \right).$$

Here, the first term on the right-hand side is the single-scattered wave containing object information, and the second term is the multiple-scattered waves that have the same wavevector and flight time as those of the single-scattered wave. The rest of the multiple-scattered waves can be ruled out by the time-gated detection^10^. $P_{\mathrm{i}}^{\mathrm{a}}\left( {{\vec{\mathbf k}}^{\mathrm{i}}} \right) = P\left( {{\vec{\mathbf k}}^{\mathrm{i}}} \right) \cdot {\mathrm{exp}}\left[ { - i\phi _{\mathrm{i}}\left( {{\vec{\mathbf k}}^{\mathrm{i}}} \right)} \right]$ and $P_{\mathrm{o}}^{\mathrm{a}}\left( {{\vec{\mathbf k}}^{\mathrm{o}}} \right) = P\left( {{\vec{\mathbf k}}^{\mathrm{o}}} \right){\mathrm{exp}}\left[ { - i\phi _{\mathrm{o}}\left( {{\vec{\mathbf k}}^{\mathrm{o}}} \right)} \right]$ are the complex pupil functions for the illumination and imaging paths, respectively, where $P\left( {{\vec{\mathbf k}}} \right)$ is the pupil function of the ideal objective lens ($P\left( {{\vec{\mathbf k}}} \right) = 1$ for $\left| {{\vec{\mathbf k}}} \right| \le k_0\alpha$, with *α* being the numerical aperture of the objective lens and *k*_0_ the magnitude of the wavevector in free space; otherwise *P* = 0). The factor $\gamma = {\mathrm{exp}}\left[ { - 2L/l_{\mathrm{s}}} \right]$ describes the intensity attenuation of the single-scattered wave for the round trip through the scattering medium. *β* is the average intensity of the multiple-scattered waves detected at the camera, which is determined by the imaging optics, the time-gating window, and the optical properties of the scattering medium. The single-scattered wave can be obscured by multiple-scattered waves, because *γ*/*β* is reduced to well below unity with increasing target depth.

To enlighten the effects of scattering and aberration in image formation, we prepared an asymmetric aberrating layer containing a cylindrical groove with a radius of curvature of 6 mm (Fig. 1b). The layer was made of 1-mm-thick clean polydimethylsiloxane (PDMS, refractive index: 1.41). Because of the refractive index mismatch between the layer and the immersion medium (refractive index of water: 1.33), the cylindrical groove causes asymmetric aberrations such as astigmatisms. A 7*l*_s_-thick scattering layer was placed underneath this aberrating layer. The scattering layer was fabricated by dispersing polystyrene beads (diameter of 1 μm) in PDMS, and its scattering mean free path was determined by measuring the intensity of ballistic photons as a function of the thickness of the layer (Supplementary note 3). The typical scattering mean free path of the samples used in the experiment was *l*_s_ = 102 μm. This arrangement of the phantom sample allowed us to independently control the aberration and scattering. The composite layer was placed on the top of a resolution target fabricated by focused-ion-beam (FIB) milling of a gold-coated slide glass (gray scale image in Fig. 1b). The separation between the finest lines was 600 nm, which corresponds to the theoretical resolving power given by the numerical aperture of 0.8 at the wavelength of 800 nm. In the absence of the scattering and aberrating layers, the conventional incoherent imaging clearly resolved all the structures (Fig. 1c). On the other hand, fine structures were obscured by the aberrating layer (Fig. 1d), and the addition of the scattering layer completely washed out all the structures (Fig. 1e).

### Experimental recording of a time-resolved reflection matrix

**Fig. 2:**
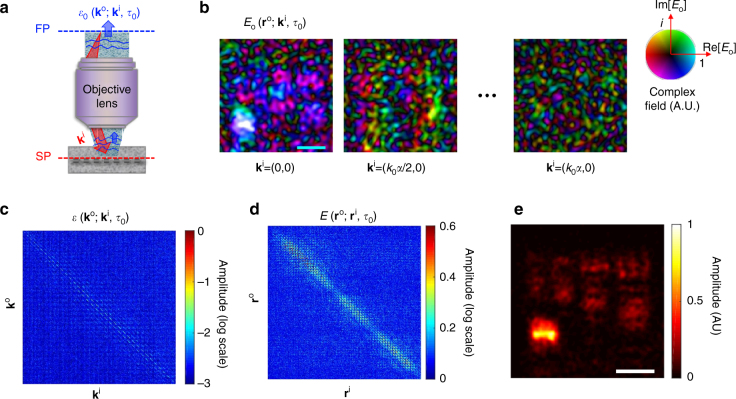
Experimental measurements of a time-resolved reflection matrix. **a** Layout of the experimental recording of the time-resolved reflection matrix. For each incident wavevector ${\vec{\bf k}}^{\mathrm{i}}$, the complex field maps of the backscattered waves were recorded at the flight time of the single-scattered waves. SP sample plane, FP Fourier plane. **b** Complex field maps of the backscattered waves recorded experimentally. Only a few representative images are shown out of the total 2800 images. Scale bar, 4 μm. Images are normalized by their respective maximum amplitudes. The saturation and colour of the colour bar indicates the amplitude and phase of the complex field, respectively, and *i* = ${\sqrt {(-1)}}$. **c** Reconstructed time-resolved reflection matrix ${\cal E}\left( {{\vec{\bf k}}^{\mathrm{o}};{\vec{\bf k}}^{\mathrm{i}},\tau _0} \right)$ in the wavevector space. Column and row indices are ${\vec{\bf k}}^{\mathrm{i}}$ and ${\vec{\bf k}}^{\mathrm{o}}$, respectively. Color bar, log-scaled amplitude. **d** Time-resolved reflection matrix $E\left( {{\vec{\mathbf r}}^{\mathrm{o}};{\vec{\mathbf r}}^{\mathrm{i}},\tau _0} \right)$ in the real space converted from (**c**). Column and row indices are ${\vec{\bf r}}^{\mathrm{i}}$ and ${\vec{\bf r}}^{\mathrm{o}}$, respectively. Color bar, amplitude in arbitrary unit. **e** Reconstructed object image from the diagonal components in (**d**). Color bar, intensity normalized by the maximum intensity. Scale bar, 4 μm

To identify the aberrations in the illumination and imaging paths, we first recorded the amplitude and phase maps of the backscattered waves from the sample for various illumination angles, or, equivalently, the incident wavevectors ${\vec{\mathbf k}}^{\mathrm{i}}$ (Fig. 2a). The output beam from a Ti:Sapphire laser (center wavelength: 800 nm, temporal pulse width: 80 fs, and average output power: 600 mW) was used as a light source. A water-dipping type objective lens (Nikon, CFI-Apo-40XW-NIR) with a numerical aperture of *α* = 0.8 was used for sub-micron resolution imaging. For the illumination, we wrote a set of 2800 random phase patterns on a liquid-crystal spatial light modulator (SLM, Hamamatsu Photonics, X10468) located at the conjugate plane of the sample. By using the basis conversion (Methods section) we retrieved images for 2800 incident wavevectors covering all the orthogonal free modes determined by the illumination area of 30 × 30 μm^2^ and the numerical aperture of the objective lens. The use of random basis was critical in removing uncontrolled phase shifts introduced in interferometric imaging. The time-resolved detection was conducted by the low-coherence interferometry (Supplementary note 3 for detailed experimental setup). The depth gating window given by the bandwidth of the light source was about 12 μm. The total acquisition time for the entire set of images was about 5 min, but it can potentially be reduced below 1 s if a high-speed SLM and camera are used. Figure 2b shows few representative complex-field maps of backscattered waves $E\left( {{\vec{\mathbf r}}^{\mathrm{o}};{\vec{\mathbf k}}^{\mathrm{i}},\tau _0} \right)$ retrieved at the sample plane ${\vec{\mathbf r}}^{\mathrm{o}} = \left( {x_{\mathrm{o}},y_{\mathrm{o}}} \right)$ for the target object shown in Fig. 1b. By taking the Fourier transform of these images, we could obtain the angular spectrum of backscattered waves ${\cal E}\left( {{\vec{\mathbf k}}^{\mathrm{o}};{\vec{\mathbf k}}^{\mathrm{i}},\tau _0} \right)$ for each ${\vec{\mathbf k}}^{\mathrm{i}}$. Figure 2c shows ${\cal E}\left( {{\vec{\mathbf k}}^{\mathrm{o}};{\vec{\mathbf k}}^{\mathrm{i}},\tau _0} \right)$ in the form of matrix with its column and row indices set by ${\vec{\mathbf k}}^{\mathrm{i}}$ and ${\vec{\mathbf k}}^{\mathrm{o}}$, respectively. This matrix is known as time-resolved reflection matrix in the wavevector space^10^.

From ${\cal E}\left( {{\vec{\mathbf k}}^{\mathrm{o}};{\vec{\mathbf k}}^{\mathrm{i}},\tau _0} \right)$, we could reconstruct an object image using CASS microscopy^10^. This can be done by simply converting the wavevector bases, ${\vec{\mathbf k}}^{\mathrm{i}}$ and ${\vec{\mathbf k}}^{\mathrm{o}}$, to the position bases, ${\vec{\mathbf r}}^{\mathrm{i}}$ and ${\vec{\mathbf r}}^{\mathrm{o}}$, respectively. In doing so, the time-resolved reflection matrix in the real space, $E\left( {{\vec{\mathbf r}}^{\mathrm{o}};{\vec{\mathbf r}}^{\mathrm{i}},\tau _0} \right)$, can be obtained (Fig. 2d) (Methods section). In Fig. 2d, we can observe that the single-scattered waves were significantly spread to off-diagonal elements due to the strong aberrations, and the speckled multiple-scattered waves are superimposed on the top of these single-scattered waves. Here, the diagonal elements represent the amplitude of backscattered waves where the point of illumination coincides with that of detection. Therefore, sampling the diagonal elements is equivalent to applying confocal gating, and the image reconstruction by the diagonal elements leads to wide-field OCM imaging (Fig. 2e). Due to the pronounced sample-induced aberrations, the confocal and temporal gating operations did not work properly and the target structures, especially fine structures, were not resolved.

### Simultaneous suppression of aberration and scattering

The confocal gating is susceptible to the aberration as well as scattering. This can clearly be seen in the angular spectrum of CASS microscopy, or equivalently OCM, which is mathematically expressed as

2 $${\cal E}_{{\mathrm{CASS}}}\left( {{\mathrm{\Delta }}{\vec{\mathbf k}}} \right) 	= \mathop {\sum}\limits_{\vec k^i} {\cal E} \left( {{\vec{\mathbf k}}^{\mathrm{i}} + {\mathrm{\Delta }}{\vec{\mathbf k}};{\vec{\mathbf k}}^{\mathrm{i}},\tau _0} \right) \\ 	= \sqrt \gamma {\cal O}\left( {{\mathrm{\Delta }}{\vec{\mathbf k}}} \right) \cdot \mathop {\sum}\limits_{{\vec{\mathbf k}}^{\mathrm{i}}} {P_{\mathrm{i}}^{\mathrm{a}}} \left( {{\vec{\mathbf k}}^{\mathrm{i}}} \right)P_{\mathrm{o}}^{\mathrm{a}}\left( {{\vec{\mathbf k}}^{\mathrm{i}} + {\mathrm{\Delta }}{\vec{\mathbf k}}} \right) \\ 	+ \sqrt \beta \mathop {\sum}\limits_{{\vec{\mathbf k}}^{\mathrm{i}}} {{\cal E}_{\mathrm{o}}^{\mathrm{M}}} \left( {{\vec{\mathbf k}}^{\mathrm{i}} + {\mathrm{\Delta }}{\vec{\mathbf k}},\tau _0} \right).$$

The angular spectrum is determined by the coherent addition of the elements of ${\cal E}\left( {{\vec{\mathbf k}}^{\mathrm{o}};{\vec{\mathbf k}}^{\mathrm{i}},\tau _0} \right)$, whose momentum difference ${\vec{\mathbf k}}^{\mathrm{o}} - {\vec{\mathbf k}}^{\mathrm{i}}$ equals the object spectrum ${\mathrm{\Delta }}{\vec{\mathbf k}}$. This addition process is essential for suppressing the effect of multiple light scattering. The summation at the first term on the right-hand side of Eq. (2), which is the cross-correlation between the complex pupil functions of the illumination and imaging paths, amplifies the object function in proportion to the number of incident wavevectors, *N*_m_. In contrast, the amplitude of the multiple-scattered waves grows in proportion to $\sqrt {N_{\mathrm{m}}}$. Therefore, the signal to noise ratio of the intensity is increased from *γ/β* to (*γ*/*β*)*N*_m_, and the single scattering intensity can outgrow that of multiple scattering when $N_{\mathrm{m}}{\mathrm{  >}}\beta /\gamma$.

However, the existence of aberrations can significantly undermine the accumulation of the single scattering signal because the cross-correlation of the complex-valued pupil functions is always smaller than that in the aberration-free case due to the inequality $\left| {\mathop {\sum }\limits_{{\vec{\mathbf k}}^{\mathrm{i}}} P_{\mathrm{i}}^{\mathrm{a}}\left( {{\vec{\mathbf k}}^{\mathrm{i}}} \right)P_{\mathrm{o}}^{\mathrm{a}}\left( {{\vec{\mathbf k}}^{\mathrm{i}} + {\mathrm{\Delta }}{\vec{\mathbf k}}} \right)} \right| \le \left| {\mathop {\sum }\limits_{{\vec{\mathbf k}}^{\mathrm{i}}} P\left( {{\vec{\mathbf k}}^{\mathrm{i}}} \right)P\left( {{\vec{\mathbf k}}^{\mathrm{i}} + {\mathrm{\Delta }}{\vec{\mathbf k}}} \right)} \right|$. To quantify the effect of aberrations, we define the following parameter *η* that describes the ratio between the total accumulated single-scattering intensity with aberrations and that without aberrations:

3 $$\eta = \frac{{\mathop {\sum}\nolimits_{{\vec{\mathbf k}}^{\mathrm{i}}} {P_{\mathrm{i}}^{\mathrm{a}}} \left( {{\vec{\mathbf k}}^{\mathrm{i}}} \right)P_{\mathrm{o}}^{\mathrm{a}}\left( {{\vec{\mathbf k}}^{\mathrm{i}} + {\mathrm{\Delta }}{\vec{\mathbf k}}} \right)_{{\mathrm{\Delta }}{\vec{\mathbf k}}}^2}}{{\mathop {\sum}\nolimits_{{\vec{\mathbf k}}^{\mathrm{i}}} P \left( {{\vec{\mathbf k}}^{\mathrm{i}}} \right)P\left( {{\vec{\mathbf k}}^{\mathrm{i}} + {\mathrm{\Delta }}{\vec{\mathbf k}}} \right)_{{\mathrm{\Delta }}{\vec{\mathbf k}}}^2}} \le 1.$$

Here, $f\left( {{\mathrm{\Delta }}{\vec{\mathbf k}}} \right)_{{\mathrm{\Delta }}{\vec{\mathbf k}}}^2$ stands for the summation of the absolute square of *f* for all the possible ${\mathrm{\Delta }}{\vec{\mathbf k}}$’s. Due to aberrations, the signal to noise ratio of CASS imaging is reduced from (*γ*/*β*)*N*_m_ to (*ηγ*/*β*)*N*_m_. This suggests that the objects may not be resolvable even for *N*_m_ > *β*/*γ* if strong aberrations exist. As a point of reference, the Strehl ratio *S*, the ratio of the peak intensity of point-spread-function with and without aberration, is often used in adaptive optics for describing weak aberrations^26^. Both *S* and *η* are attenuated with the increase of aberration, but in general *S*<*η* (Supplementary note 2). Typical adaptive optics can deal with aberrations for *S* ≥ 0.1^27^. However, the degree of aberrations that we dealt with in the present study was so severe that *S* is around 1/500 (piston, tilt, and defocus terms were removed in our estimation of Strehl ratio following the convention in adaptive optics), more than an order of magnitude smaller than that the conventional adaptive optics can handle. In addition to the reduction in signal intensity, the cross-correlation adds ${\mathrm{\Delta }}{\vec{\mathbf k}}$-dependent phase retardation to the measured object function, thereby distorting the reconstructed object image.

**Fig. 3:**
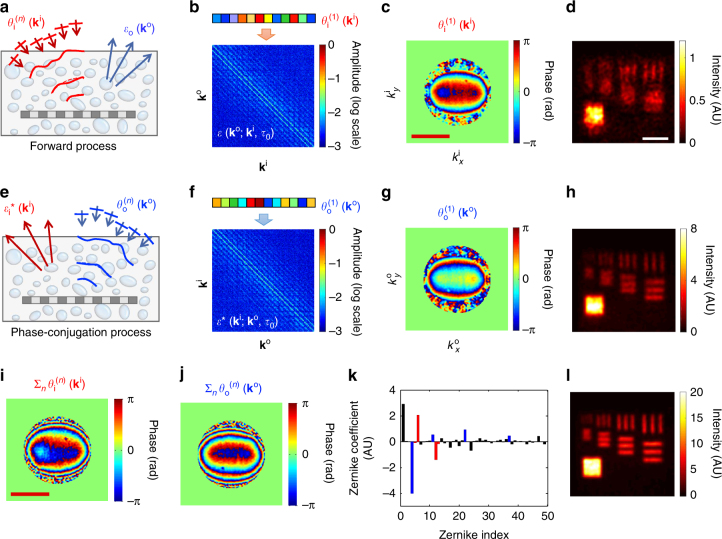
Experimental demonstration of suppressing both scattering and aberration. **a** Diagram of aberration correction for the forward process in which phase correction $\theta _{i}^{(n)}\left( {{\vec{\bf k}}^{\mathrm{i}}} \right)$ is applied to each ${\vec{\bf k}}^{\mathrm{i}}$. Here *n* is the iteration number. **b** The application of phase correction $\theta _{\mathrm{i}}^{(1)}\left( {{\vec{\bf k}}^{\mathrm{i}}} \right)$ to the columns of the time-resolved reflection matrix, ${\cal E}\left( {{\vec{\bf k}}^{\mathrm{o}};{\vec{\bf k}}^{\mathrm{i}},\tau _0} \right)$. **c** Phase map of $\theta _{\mathrm{i}}^{(1)}\left( {{\vec{\bf k}}^{\mathrm{i}}} \right)$ that maximized the total intensity of reconstructed image in the first round of iteration. Color bar, phase in radians. Scale bar, *k*_0_ *α*. **d** Reconstructed image after applying for the phase correction identified in (**c**). Scale bar, 4 μm. **e** Diagram of aberration correction in the phase-conjugation process in which the phase correction $\theta _{\mathrm{o}}^{(n)}\left( {{\vec{\bf k}}^{\mathrm{o}}} \right)$ is applied to each ${\vec{\bf k}}^{\mathrm{o}}$ in the reversal process. **f** The application of phase correction $\theta _{\mathrm{o}}^{(1)}\left( {{\vec{\bf k}}^{\mathrm{o}}} \right)$ to the columns of the phase-conjugated reflection matrix ${\cal E}\,^{*}\left( {{\vec{\bf k}}^{\mathrm{i}};{\vec{\bf k}}^{\mathrm{o}},\tau _0} \right)$. **g** Phase map of $\theta _{\mathrm{o}}^{(1)}\left( {{\vec{\bf k}}^{\mathrm{o}}} \right)$ that maximized the total intensity of the image in the first round of iteration. **h** Reconstructed image after applying for the phase correction identified in (**g**). **i**, **j** Accumulated phase-correction maps for the illumination and imaging paths, respectively, after *n* = 5 rounds of iterations. **k** Decomposition of the map in (**j**) into the first 50 Zernike polynomials *Z*_*j*_ following the convention of Noll’s sequential indices. Blue bars indicate spherical aberrations (*j* *=* 4, 11, 22, 37), and red bars astigmatisms (*j* = 5, 6, 12, 13). **l** Reconstructed image after 5 rounds of iterations. Color bars for the images in (**d**, **h**, **i**) are the intensity normalized by the maximum intensity in Fig. 2e

The method we propose here is to identify the angle-dependent phase corrections $\theta _{\mathrm{i}}\left( {{\vec{\bf k}}^{\mathrm{i}}} \right)$ and $\theta _{\mathrm{o}}\left( {{\vec{\bf k}}^{\mathrm{o}}} \right)$ for the illumination and imaging paths, respectively, that can cancel out the respective angle-dependent aberrations, $\phi _{\mathrm{i}}\left( {{\vec{\bf k}}^{\mathrm{i}}} \right)$ and $\phi _{\mathrm{o}}\left( {{\vec{\bf k}}^{\mathrm{o}}} \right)$ (Supplementary note 1). In doing so, the complex-valued pupil function is converted to the real-valued ones. The main concept is to apply $\theta _{\mathrm{i}}\left( {{\vec{\bf k}}^{\mathrm{i}}} \right)$ for each ${\vec{\bf k}}^{\mathrm{i}}$ in the forward process in such a way to maximize the total intensity, or *η*, of the reconstructed image (Fig. 3a). It turned out that this operation enables $\theta _{\mathrm{i}}\left( {{\vec{\bf k}}^{\mathrm{i}}} \right)$ to preferentially counteract $\phi _{\mathrm{i}}\left( {{\vec{\bf k}}^{\mathrm{i}}} \right)$ (Methods section). As an important additional step, we employed the phase-conjugation operation, that is, the reversal of illumination and imaging paths, and applied $\theta _{\mathrm{o}}\left( {{\vec{\bf k}}^{\mathrm{o}}} \right)$ to maximize the intensity of the reconstructed image in the reciprocal process, which then preferably corrects $\phi _{\mathrm{o}}\left( {{\vec{\bf k}}^{\mathrm{o}}} \right)$ (Fig. 3e). Repeating these operations leads to the independent identification of $\phi _{\mathrm{i}}\left( {{\vec{\bf k}}^{\mathrm{i}}} \right)$ and $\phi _{\mathrm{o}}\left( {{\vec{\bf k}}^{\mathrm{o}}} \right)$.

The detailed procedures for this CLASS algorithm are given in the following. We first multiplied initial arbitrary angle-dependent phase corrections, ${\mathrm{exp}}[ {i\theta _{\mathrm{i}}^{(1)}\left( {{\vec{\mathbf k}}^{\mathrm{i}}} \right)} ]$, to columns of ${\cal E}\left( {{\vec{\mathbf k}}^{\mathrm{o}};{\vec{\mathbf k}}^{\mathrm{i}},\tau _0} \right)$ (Fig. 3b) and identified the set of $\theta _{\mathrm{i}}^{(1)}\left( {{\vec{\mathbf k}}^{\mathrm{i}}} \right)$ that maximizes the total intensity of the reconstructed image (Fig. 3c). This can simply be done by changing the individual $\theta _{\mathrm{i}}^{(1)}\left( {{\vec{\mathbf k}}^{\mathrm{i}}} \right)$ from 0 to 2π and finding the particular value of $\theta _{\mathrm{i}}^{(1)}\left( {{\vec{\mathbf k}}^{\mathrm{i}}} \right)$ at which the total intensity is the maximum. It is important to note that mainly the single-scattered waves take part in this process and the multiple-scattered waves play little role. The maps of multiple-scattered waves taken at different angles of illumination are uncorrelated with respect to one another, and remained so even after multiplying the phase corrections. Therefore, the maximization of total intensity is almost exclusively due to the aberration correction of the single-scattered waves (Supplementary note 2). Figure 3c is the map of $\theta _{\mathrm{i}}^{(1)}\left( {{\vec{\mathbf k}}^{\mathrm{i}}} \right)$ identified by this process and the resulting image is shown in Fig. 3d in which large structures were resolved better than the original image (Fig. 2e).

The maximization operation for the forward process is incomplete and $\theta _{\mathrm{i}}^{(1)}\left( {{\vec{\mathbf k}}^{\mathrm{i}}} \right)$ cannot be the same as $\phi _{\mathrm{i}}\left( {{\vec{\mathbf k}}^{\mathrm{i}}} \right)$ unless the aberration in the imaging paths is addressed. In order to form a closed-loop correction, we developed a phase conjugation process in which the wave is incident from $- {\vec{\mathbf k}}^{\mathrm{o}}$ to $- {\vec{\mathbf k}}^{\mathrm{i}} = - \left( {{\vec{\mathbf k}}^{\mathrm{o}} - {\mathrm{\Delta }}{\vec{\mathbf k}}} \right)$ (Fig. 3e). This reverse process does not require additional data acquisition because it can be computed from the set of originally measured images by the reciprocity of wave propagation. With the correction $\theta _{\mathrm{i}}^{(1)}\left( {{\vec{\mathbf k}}^{\mathrm{i}}} \right)$ in place, we took the conjugate transpose of the original time-resolved reflection matrix ${\cal E}\left( {{\vec{\bf k}}^{\mathrm{o}};{\vec{\bf k}}^{\mathrm{i}},\tau _0} \right)$ to obtain the phase-conjugated time-resolved reflection matrix, ${\cal E}^*\left( {{\vec{\bf k}}^{\mathrm{i}};{\vec{\bf k}}^{\mathrm{o}},\tau _0} \right)$ (Fig. 3f). We then multiplied ${\mathrm{exp}}\left[ {i\theta _{\mathrm{o}}^{(1)}\left( {{\vec{\bf k}}^{\mathrm{o}}} \right)} \right]$ to the columns of ${\cal E}^*\left( {{\vec{\bf k}}^{\mathrm{i}};{\vec{\bf k}}^{\mathrm{o}},\tau _0} \right)$, and maximized the total intensity of the phase-conjugated image. Figure 3g shows the map of $\theta _{\mathrm{o}}^{(1)}\left( {{\vec{\mathbf k}}^{\mathrm{o}}} \right)$ thus identified. Figure 3h shows the image after this operation in which the fine details were better visible than before. Since the identified aberration maps are not yet complete, we iterated the aberration correction steps to improve its accuracy. The accumulated phase corrections for the illumination path, $\mathop {\sum}\nolimits_n {\theta _{\mathrm{i}}^{(n)}\left( {{\vec{\bf k}}^{\mathrm{i}}} \right)}$, and imaging path, $\mathop {\sum}\nolimits_n {\theta _{\mathrm{o}}^{(n)}}$, converged as the number of iterations *n* was increased (Fig. 3i, j, respectively). The reconstructed image could reveal the finest structures in the resolution target at the iteration number of *n* = 5 (Fig. 3l). Moreover, the signal intensity at the target estimated (Fig. 3d, i) was increased in magnitude by more than 20 times, suggesting that the cross-correlation of the aberration-corrected pupil functions had been increased. Taken together, these observations confirmed that the proposed method works extremely well, even in the presence of multiple-scattered waves.

From the acquired angle-dependent phase correction maps in Fig. 3i, j, the initial Strehl ratio (Supplementary note 2) was estimated to be *S* = 1/531. This value is two orders of magnitude smaller than the one conventional adaptive optics typically handles. We also obtained *η* = 1/204 from Eq. (3) and measured aberration maps, which means that the total single scattering intensity was increased by about 200 times after the application of CLASS algorithm. There was a discrepancy between the apparent signal enhancement of about 20 times estimated (Fig. 3d, l) and the increase of single scattering intensity by 200 times. Moreover, this was mainly because multiple-scattered waves as well as single-scattered waves contributed to the signal intensity at the target in Fig. 3d. Further analysis revealed that the initial single-to-multiple scattering intensity ratio of individual angle-dependent images was $\gamma /\beta \simeq 0.007$, and the initial single-to-multiple scattering intensity ratio of the reconstructed image was $\eta \left( {\gamma /\beta } \right)N_{\mathrm{m}} \simeq 0.1$ (Supplementary note 3).

The acquired angle-dependent phase corrections shown in Fig. 3i, j presented asymmetry due to the cylindrical groove in the aberrating layer. The phase corrections along the *k*_y_ direction have much steeper variations than those along the *k*_x_ direction. The input and output aberration maps are largely the same as waves travel back and forth through the same sample physically in the reflection geometry. But, due to the difference in the system aberrations between the illumination beam path from the light source to the beam splitter and the collection beam path from the beam splitter to the camera, they have a slight difference (Supplementary note 3). We decomposed the output phase correction map in Fig. 3j into Zernike polynomials (*Z*_*j*_), which are widely used to explain these types of aberrations. Because the aberration correction of CLASS microscopy was performed at the spatial frequency resolution of 1/30 μm^−1^, we could identify high-order Zernike modes that ordinary adaptive optical microscopy cannot. In Fig. 3k we show only the first 50 coefficients according to Noll’s sequential indices^28^. As expected, the dominant components were the astigmatism (Zernike indices of 5, 6, 12, and 13, red bars) induced by the cylindrical aberrating layer and the spherical aberration (Zernike indices of 4, 11, 22, 37, blue bars) induced by the overall index mismatch between the immersion medium and the scattering layers.

### High resolution imaging of targets in biological tissues

**Fig. 4:**
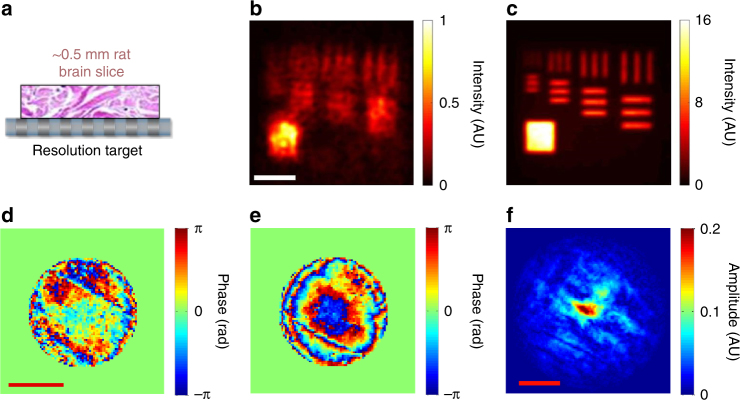
Experimental demonstration of aberration correction for a target underneath a rat brain tissue. **a** Layout of the sample geometry. A thick rat brain tissue slice was placed on the top of the resolution target. **b**, **c** CLASS images of a resolution target under a 500 μm-thick rat brain tissue layer before and after aberration correction, respectively. Scale bar, 4 μm. Color bars, intensity normalized by the maximum intensity in (**b**, **d** and **e**) Input and output phase correction maps, respectively. Scale bar, *k*_0_ *α*. Color bars, phase in radians. **f** Amplitude transfer function calculated by cross-correlation between (**d**, **e**). Scale bar, *k*_0_ *α*

In general, biological tissues exhibit much more complicated aberrations than the phantoms, making high-resolution imaging even more difficult. We demonstrated the performance of CLASS microscopy for targets located under a layer of a biological tissue. A 500 μm-thick slice of a rat brain tissue, whose scattering mean free path was measured to be approximately 100 μm, was placed on the top of the resolution target (Fig. 4a) (Methods section for the preparation of brain tissues). As shown in Fig. 4b, the reconstructed image before the aberration correction could not reveal the fine structures of the resolution target due to the multiple scattering noise and the aberration induced by the tissue. On the other hand, when full aberration correction functionality of CLASS microscopy was used, the targets were clearly visible up to the line spacing of 600 nm (Fig. 4c). Also, the intensity fluctuation in the gold-coated area was remarkably reduced, supporting the conclusion that the aberration correction process properly accumulated the single-scattered waves.

The angle-dependent phase corrections for illumination and reflection identified for the biological tissues are shown in Fig. 4d, e, respectively. Unlike the results shown in Fig. 3i, j, irregular patterns appeared due to the complex internal structures in the brain tissue. In Fig. 4f, we present the amplitude transfer function of the rat brain tissue created by calculating the cross-correlation of input and output aberration maps. The color scale is normalized by the maximum value of the ideal amplitude transfer function. The attenuated value of the amplitude transfer function and reduced bandwidth were responsible for the deterioration of the image in Fig. 4b. The initial Strehl ratio was estimated to be about 1/662 from the acquired aberration maps. In addition, while the phase gradient of aberration is relatively flat at low spatial frequency, it steepens at higher spatial frequencies. This difference underlies the necessity of compensating for specimen-induced aberrations in ultra-high resolution imaging.

### Demonstration of CLASS microscopy for biological specimens

**Fig. 5:**
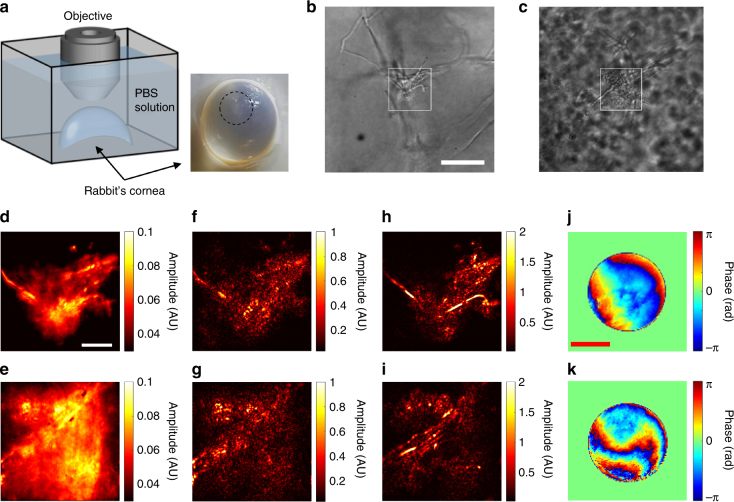
Experimental demonstration of CLASS microscopy for imaging the hyphae of Aspergillus cells in a rabbit’s cornea. **a** Layout of the sample geometry. A rabbit’s cornea infected by *A. fumigatus* was immersed in PBS solution in a Petri dish and placed under the objective lens. The black dashed circle in the photo indicates the infection site. **b**, **c** Transmission images taken by illuminating the cornea from the bottom of the Petri dish in (**a**) using a light emitting diode (*λ* = 780 nm). Scale bar, 40 µm. **d**, **e** Incoherent addition of time-gated and angle-dependent reflection images for the white rectangular boxes are shown in (**b**, **c**) respectively. Scale bar, 10 µm. **f**, **g** Images taken by CASS microscopy in the epi-detection geometry for the white rectangular boxes are shown in (**b**, **c**) respectively. **h**, **i** Same as (**f**, **g**) respectively, but after applying CLASS algorithm. Color bar, intensity in arbitrary unit. **j**, **k** Specimen-induced aberration maps identified during the acquisition of (**h**, **i**) respectively. Scale bar, *k*_0_ *α*. Color bar, phase in radians

Finally, we demonstrated the performance of CLASS microscopy for imaging biological specimens. We prepared an ex vivo fungal keratitis rabbit cornea infected by *A. fumigatus* (Fig. 5a) and performed imaging of individual fungal cells therein (Methods section for the details of sample preparation procedure). *A. fumigatus* is a group of fungi that can be found everywhere. It can cause infection to people with poor immune systems in their brains or other organs including the eye, the heart, the kidneys and the skin, and severely undermine the functionalities of such organs. In the case of eye infection, the penetration of fungi and the disruption of corneal microenvironment by inflammation response make the cornea opaque (black dashed circle in Fig. 5a), which often leads to the complete loss of vision. The fungi act as scattering particles because their size is typically on the order of a few microns and their refractive index is different from the surrounding cornea. Multiple light scattering and aberrations caused by these fungal cells make it extremely difficult to perform imaging inside cornea. As such, the depth at which the infection has progressed can hardly be visualized by conventional imaging modalities such as optical coherence tomography and confocal microscopy. In our experiment, we performed imaging of Aspergillus cells deep inside the infection sites and successfully visualized their fine multicellular filaments called hyphae.

As a point of reference, we first took transmission images of the cornea at two different sites (Fig. 5b, c) by illuminating light emitting diode from the bottom of the specimen. Since imaging in transmission experiences the effect of scattering much less than the epi-detection, the structures can still be visible in cases like Fig. 5b where the depth of the fungal cells was relatively shallow. But even the transmission mode of imaging failed to visualize the fine details of the fungal cells when they are located 300 µm or more from the surface of the cornea (Fig. 5c). The structures were distorted significantly and the background noise due to other fungal cells became strong. In any case, the transmission mode of imaging is not relevant to in vivo imaging as light source cannot be put inside the specimens. For the view fields indicated as rectangular boxes (Fig. 5b, c), we took a set of time-resolved reflection images in the epi-detection geometry for various angles of illumination. The incoherent addition of these images (Fig. 5d, e), which are equivalent to the images of angular compounding optical coherence tomography, could not visualize the detailed structures of the fungi due to the multiple light scattering and aberration caused by the fungi located at the upper layers. From the recorded images, we constructed a time-resolved reflection matrix and acquired CASS images (Fig. 5f, g), but these images failed to visualize the fine filaments, too. Finally, we applied CLASS algorithm and corrected the specimen-induced aberrations (Fig. 5h, i). This has led to the clear visualization of the filaments, which is the objective indication of the infection. The aberration maps identified by the CLASS microscopy in Fig. 5j, k show highly irregular patterns induced by the thick layer of fungi. The Strehl ratio *S*, single-scattering enhancement factor *η*, and initial single-to-multiple scattering ratio in the position basis $\left( {\eta \gamma /\beta } \right)N_{\mathrm{m}}$ were 1/437, 1/44.7 and 0.23, respectively, for the specimen in Fig. 5h, and 1/890, 1/68.2, and 0.04, respectively, for the specimen in Fig. 5i. The implication of these results is that CLASS microscopy will help us to make an accurate diagnosis and proper treatments of such diseases as a fungal infection and a cataract that affect to about 30% of the entire population. Since our measurements were performed in the epi-detection geometry with no use of labeling agents, the proposed method can readily be applicable to the real practices.

## Discussion

We presented an optical coherence imaging method that can perform sub-micron resolution imaging of targets located up to the depth of 7 scattering mean free paths. The proposed method introduced separate angle-dependent phase corrections for the illumination and reflection paths to preferentially optimize the total intensity of single-scattered waves in both forward and phase-conjugation processes. Our method is unique in four major respects. First, the optimization operation of the total intensity inthe momentum difference space acted mainly on the single-scattered waves, rather than the multiple-scattered ones. For this reason, the proposed method could be effective even with multiple scattering backgrounds. Second, due to the use of the closed-loop operations, aberrations for the illumination and reflection paths could be independently identified without the need for a guide star^26^. Unlike fluorescence imaging where the correction of aberrations from the illumination path is the only concern, this capability is especially critical for coherent imaging because aberrations from both paths are responsible for the reduced SNR. The third important aspect is that aberration correction is performed over a view field with finely stepped illumination angles. This enables our method to eliminate the steep angle-dependent phase retardations that thick biological tissues induce at large propagating angles and to outperform conventional adaptive optics microscopy. In addition, due to the aberration correction for all the orthogonal modes within the view field, diffraction-limit spatial resolution can be obtained across the entire view field. Finally, the aberration correction is performed in post-processing after the acquisition of the time-resolved reflection matrix. Given the same number of angular bases to correct, this post processing step is much faster than the experimental feedback iteration required for wavefront control. The speed of the current implementation is largely limited by the speed of the SLM, but the use of a high-speed binary control SLM with a high-speed camera would substantially reduce the detection time.

The ability to perform ultra-high spatial resolution imaging deep within scattering media will open new possibilities for studying important biological reactions in detail. Since our imaging method relies on reflectance, not the fluorescence, it can readily be applicable to in vivo imaging with no need of labeling agents. On the other hand, CLASS microscopy requires target samples to have sufficient reflectivity for its best performance in imaging depth similar to the other coherent imaging methods working in the epi-detection geometry. In fact, the maximum possible imaging depth is determined by the reflectivity of the sample as well as the scattering properties of the scattering medium. If the reflectivity of the sample is reduced by 1/10, then the imaging depth should be reduced by 1 scattering mean free path. In addition to the successful demonstration of imaging deep within a rabbit’s cornea infected by fungi, the applications of this method can be extended to early disease diagnosis, studies of nervous systems and of the activities of stem cells inside bone marrow, and so on. The proposed method can be applicable to other coherent imaging modalities such as second-harmonic generation microscopy and stimulated Raman scattering microscopy to increase their imaging depth limits in a similar way. Moreover, this new CLASS microscopy can offer specimen-induced aberration maps with minimal photo-bleaching to multiphoton fluorescence microscopy techniques^22^. Ultimately, our study will widen the scope of the applications that optical microscopy can explore.

## Methods

### Random basis and basis conversion to wavevector space

In the experiment, we used a set of random patterns composed of multiple plane waves as a basis of illumination rather than individual angular plane waves. Interferometric imaging suffers from uncontrolled phase shifts due to the mechanical fluctuation of the relative path length between the sample and reference waves. When using plane waves, these phase shifts are indistinguishable from the angle-dependent phase retardation caused by the sample, meaning that the sample-induced aberrations cannot be identified. When using random patterns, however, multiple plane waves of known relative phases are simultaneously injected to the sample such that we can exclusively deal with sample-induced phase shifts.

For the basis conversion, we recorded a time-resolved complex field map $u_{\mathrm{i}}({\vec{\bf r}}_{\mathrm{i}};j,\tau _0)$ for the *j* ^th^ random pattern written on the SLM by placing an ideal mirror at the sample plane for the arrival time τ_0_ associated with the depth of the mirror. For the same set of illumination patterns, the complex-field map $u_{\mathrm{o}}({\vec{\bf r}}_{\mathrm{o}};j,\tau _0)$ of the reflected waves was recorded for the sample embedded in the scattering medium (Supplementary note 3 for representative images). By taking the Fourier transform of $u_{\mathrm{i}}({\vec{\bf r}}_{\mathrm{o}};j,\tau _0)$ and $u_{\mathrm{o}}({\vec{\bf r}}_{\mathrm{o}};j,\tau _0)$, we constructed the matrices describing the incident and reflected waves, ${\cal U}_{\mathrm{i}}\left( {{\vec{\bf k}}^{\mathrm{i}};j,\tau _0} \right)$, and ${\cal U}_{\mathrm{o}}\left( {{\vec{\bf k}}^{\mathrm{o}};j,\tau _0} \right)$, respectively. From the product ${\cal U}\left( {{\vec{\bf k}}^{\mathrm{o}};j} \right) \cdot {\cal U}_{\mathrm{i}}^{ - 1}\left( {{\vec{\bf k}}^{\mathrm{i}};j} \right)$, we could construct the time-resolved reflection matrix ${\cal E}\left( {{\vec{\bf k}}^{\mathrm{o}};{\vec{\bf k}}^{\mathrm{i}},\tau _0} \right)$, which is shown in Fig. 2c. This procedure led to the conversion of the initial random speckle basis into the basis of transverse wavevectors. By taking the inverse Fourier transform of the matrix with respect to ${\vec{\bf k}}^{\bf{o}}$, we could obtain $E\left( {{\vec{\bf r}}^{\mathrm{o}};{\vec{\bf k}}^{\mathrm{i}},\tau _0} \right)$, which are the complex field maps at the sample plane for each incident wavevector. Some of the representative images are shown in Fig. 2b.

The time-resolved reflection matrix ${\cal E}\left( {{\vec{\bf k}}^{\mathrm{o}};{\vec{\bf k}}^{\mathrm{i}},\tau _0} \right)$ in the wavevector space can be converted to that in the position space (Fig. 2d), i.e. $E\left( {{\vec{\bf r}}^{\mathrm{o}};{\vec{\bf k}}^{\mathrm{i}},\tau _0} \right)$, by taking the inverse Fourier transforms with respect to ${\vec{\bf k}}^{\mathrm{i}}$ and ${\vec{\bf k}}^{\bf{o}}$. Alternatively, it can be constructed by the matrix multiplication, $u_{\mathrm{o}}({\vec{\bf r}}_{\mathrm{o}};j,\tau _0) \cdot u_{\mathrm{i}}\left( {{\vec{\bf r}}_{\mathrm{o}};j,\tau _0} \right)^{ - 1}$.

### Convergence of CLASS algorithm

The CLASS algorithm described in Fig. 3 is mathematically described in the following. We first applied initial arbitrary angle-dependent phase corrections $\theta _{\mathrm{i}}^{(1)}\left( {{\vec{\bf k}}^{\mathrm{i}}} \right)$ to the spectrum of the CASS image to form a CLASS image, that is ${\cal E}_{{\mathrm{CLASS}}}^{(1)}\left( {{\mathrm{\Delta }}{\vec{\bf k}}} \right) = \mathop {\sum }\limits_{{\vec{\mathbf k}}^{\mathrm{i}}} {\cal E}_{\mathrm{o}}\left( {{\vec{\bf k}}^{\mathrm{i}} + \Delta {\vec{\bf k}}} \right)e^{i\theta _{\mathrm{i}}^{(1)}\left( {{\vec{\mathbf k}}^{\mathrm{i}}} \right)}.$ Then we identified the set of $\theta _{\mathrm{i}}^{(1)}\left( {{\vec{\bf k}}^{\mathrm{i}}} \right)$ that maximizes the total intensity of the CLASS image:

4 $$\mathop {{{\mathrm{max}}}}\limits_{\theta _{\mathrm{i}}^{(1)}\left( {{\vec{\mathbf k}}^{\mathrm{i}}} \right)} \mathop {\sum }\limits_{{\mathrm{\Delta }}{\vec{\mathbf k}}} \left| {{\cal E}_{{\mathrm{CLASS}}}^{(1)}\left( {{\mathrm{\Delta }}{\vec{\bf k}}} \right)} \right|^2.$$

Through this maximization process, $\theta _{\mathrm{i}}^{(1)}\left( {{\vec{\bf k}}^{\mathrm{i}}} \right)$ converges to the input aberration $\theta _{\mathrm{i}}\left( {{\vec{\bf k}}^{\mathrm{i}}} \right)$. This becomes evident when examining the cross-term between two representative incident wavevectors, ${\vec{\bf k}}_1^{\mathrm{i}}$ and ${\vec{\bf k}}_2^{\mathrm{i}}\left( { \ne {\vec{\bf k}}_1^{\mathrm{i}}} \right)$, in Eq. (4):

5 $$\begin{array}{l}{\mathrm{exp}}{\kern 1pt} i\left\{ {\phi _{\mathrm{i}}\left( {{\vec{\bf k}}_2^{\mathrm{i}}} \right) - \phi _{\mathrm{i}}\left( {{\vec{\bf k}}_1^{\mathrm{i}}} \right)} \right\}{\kern 1pt} {\mathrm{exp}}{\kern 1pt} \left[ { - i\left\{ {\theta _{\mathrm{i}}^{(1)}\left( {{\vec{\bf k}}_2^{\mathrm{i}}} \right) - \theta _{\mathrm{i}}^{(1)}\left( {{\vec{\bf k}}_1^{\mathrm{i}}} \right)} \right\}} \right]\\ \quad \cdot \left[ {\mathop {\sum}\limits_{{\mathrm{\Delta }}{\vec{\bf k}}} {\left\{ {{\cal O}\left( {{\mathrm{\Delta }}{\vec{\bf k}}} \right){\kern 1pt} P_{\mathrm{o}}^{\mathrm{a}}\left( {{\mathrm{\Delta }}{\vec{\bf k}} + {\vec{\bf k}}_1^{\mathrm{i}}} \right)} \right\}\,\left\{ {{\cal O}\left( {{\mathrm{\Delta }}{\vec{\bf k}}} \right){\kern 1pt} P_{\mathrm{o}}^{\mathrm{a}}\left( {{\mathrm{\Delta }}{\vec{\bf k}} + {\vec{\bf k}}_2^{\mathrm{i}}} \right)} \right\}^*} } \right].\end{array}$$

If there was no aberration in the reflection beam path, then $P_{\mathrm{o}}^{\mathrm{a}} = P_{\mathrm{o}}$. The phase angle of the term in the square brackets [] in Eq. (5), which we define as ${\it{\Phi }}_{\mathrm{i}}^{(1)}\left( {{\vec{\bf k}}_1^{\mathrm{i}},{\vec{\bf k}}_2^{\mathrm{i}}} \right)$, would be zero such that $\theta _{\mathrm{i}}^{(1)}\left( {{\vec{\bf k}}_2^{\mathrm{i}}} \right) - \theta _{\mathrm{i}}^{\left( 1 \right)}\left( {{\vec{\bf k}}_1^{\mathrm{i}}} \right) = \phi _{\mathrm{i}}\left( {{\vec{\bf k}}_2^{\mathrm{i}}} \right) - \phi _{\mathrm{i}}\left( {{\vec{\bf k}}_1^{\mathrm{i}}} \right)$. Since only relative phase matters, we can set $\theta _{\mathrm{i}}^{(1)}\left( {{\vec{\bf k}}_1^{\mathrm{i}}} \right) = 0$ and $\phi _{\mathrm{i}}\left( {{\vec{\bf k}}_1^{\mathrm{i}}} \right) = 0$ at ${\vec{\bf k}}_{\mathrm{1}}^{\mathrm{i}}{\mathrm{ = 0}}$. Then $\theta _{\mathrm{i}}^{(1)}\left( {{\vec{\bf k}}^{\mathrm{i}}} \right)$ is equal to $\phi _{\mathrm{i}}\left( {{\vec{\bf k}}^{\mathrm{i}}} \right)$ for arbitrary ${\vec{\bf k}}^{\mathrm{i}}$, suggesting that the aberration in the illumination path is perfectly corrected. However, aberration also develops through the reflection process, and ${\it{\Phi }}_{\mathrm{i}}^{(1)}\left( {{\vec{\bf k}}_1^{\mathrm{i}},{\vec{\bf k}}_2^{\mathrm{i}}} \right)$ would be non-zero and consequently act as the error for the aberration correction.

For the aberration correction to be effective, the correction error ${\it{\Phi }}_{\mathrm{i}}^{(1)}\left( {{\vec{\bf k}}_1^{\mathrm{i}},{\vec{\bf k}}_2^{\mathrm{i}}} \right)$ should have a finite width of distribution around zero, not a random and uniform distribution between −π and +π. This becomes possible when $P_{\mathrm{o}}^{\mathrm{a}}\left( {{\mathrm{\Delta }}{\vec{\bf k}} + {\vec{\bf k}}^{\mathrm{i}}} \right)$ is a slowly varying function with respect to ${\mathrm{\Delta }}{\vec{\bf k}}$. If we record individual images over a wide view field, then the spectral resolution of the individual complex field images, which is the reciprocal of the width of view field, can be fine enough to make $P_{\mathrm{o}}^{\mathrm{a}}$ a slowly varying function (Supplementary note 2 for the numerical simulation on the convergence condition). The maximization process in Eq. (5) lead to $\theta _{\mathrm{i}}^{(1)}\left( {{\vec{\bf k}}^{\mathrm{i}}} \right) \approx \phi _{\mathrm{i}}\left( {{\vec{\bf k}}^{\mathrm{i}}} \right)$ up to the accuracy given by the width of the distribution of ${\it{\Phi }}_{\mathrm{i}}^{(1)}\left( {{\vec{\bf k}}_1^{\mathrm{i}},{\vec{\bf k}}_2^{\mathrm{i}}} \right)$. As iteration number increases, the width of the distribution of ${\it{\Phi }}_{\mathrm{i}}^{(1)}\left( {{\vec{\bf k}}_1^{\mathrm{i}},{\vec{\bf k}}_2^{\mathrm{i}}} \right)$ was reduced and so were the aberration correction errors (Supplementary note 2). Mathematical description for the phase-conjugation is similar to that for the forward process, and it can be shown that $\theta _{\mathrm{o}}\left( {{\vec{\bf k}}^{\mathrm{o}}} \right)$ converges to $\phi _{\mathrm{o}}\left( {{\vec{\bf k}}^{\mathrm{o}}} \right)$ (Supplementary note 1).

### Brain tissue preparation

The brain slices were made from 2- to 3-day-old Sprague–Dawley rats. The brains were quickly excised and dropped into ice-cold artificial cerebrospinal fluid (ACSF). Coronal slices were cut at 500 µm using a vibroslicer (World Precision Instruments, Sarasota, FL, USA) and fixed for overnight at 4 °C in 4% paraformaldehyde. After fixation, brain slices were mounted on the resolution target for imaging. All experimental procedures and protocols above were in accordance with the guidelines (approval number KUIACUC-2017-83) established by the Committee of Animal Research Policy of Korea University.

### Preparation of a Rabbit’s cornea infected by fungi

Three New Zealand white rabbits, weighting 1.5–2.5 kg, were used for the *A. fumigatus*-infected cornea model. All animal treatment procedure was approved by Institutional Animal Care and Use Committee (IACUC, approval number 2015-13-096) of Asan Medical Center (AMC). Intrastromal infection method was used to induce fungal corneal keratitis. Rabbits were anesthetized by intramuscular (IM) injection of the mixture of ketamine (40 mg kg^−1^ body weight) and xylazine (10 mg kg^−1^ body weight) prior to the procedure. Central cornea of the rabbit eye was gently scrapped with a 15–0–0 surgical blade until the stromal layer was exposed. After scrapping the epithelium, a surgical needle (30 gauge) containing aspergillus paste was directly inserted into the stroma. After 5–7 days, the rabbit eyes were examined for infection by a slit-lamp microscope. If the rabbit corneas had haze ulcer and corneal scar, then the rabbits were euthanized and the infected rabbit eyeballs were enucleated. The extracted rabbit eyeballs were fixed in 4% formalin solution at 4 °C for at least 24 h. After fixation, the infected corneas were torn off from the whole eye globes, and stored in PBS solution.

### Data availability

All relevant data are available from the authors.

## Electronic supplementary material

Supplementary Information

Peer Review File_NEW
