# Supplementary material for: High-resolution adaptive optical imaging within thick scattering media using closed-loop accumulation of single scattering
Source: Nat Commun. 2017 Dec 18;8:2157. doi: 10.1038/s41467-017-02117-8 (PMC5735168; doi:10.1038/s41467-017-02117-8)
Supplement: Supplementary file 1 — Supplementary Information [file 41467_2017_2117_MOESM1_ESM.pdf]

Supplementary Note 1. Theoretical framework for the CLASS microscopy

As discussed in the main text, the maximization of the total intensity of CLASS image leads to the finding of angle-dependent aberrations. In this section, we provide detailed mathematical description of its working principle and conditions for the convergence.

Mathematical formulation of the aberration correction for the input

Let us begin with the first round of iteration for the aberration correction in the illumination beam path. After applying for initial arbitrary angle-dependent phase corrections $\theta_{\mathrm{i}}^{(1)}(\vec{\mathbf{k}}^{\mathrm{i}})$, the spectrum of the CLASS image is written as

$$\mathcal{E}_{\mathrm{CLASS}}^{(1)}(\Delta\vec{\mathbf{k}}) = \sum_{\vec{\mathbf{k}}^{\mathrm{i}}} \mathcal{E}_{\mathrm{o}}(\vec{\mathbf{k}}^{\mathrm{i}} + \Delta\vec{\mathbf{k}})\, e^{i\theta_{\mathrm{i}}^{(1)}(\vec{\mathbf{k}}^{\mathrm{i}})} = \sqrt{\gamma}\mathcal{O}(\Delta\vec{\mathbf{k}}) \cdot \sum_{\vec{\mathbf{k}}^{\mathrm{i}}} P_{\mathrm{i}}^{\mathrm{a}}(\vec{\mathbf{k}}^{\mathrm{i}}) P_{\mathrm{o}}^{\mathrm{a}}(\vec{\mathbf{k}}^{\mathrm{i}} + \Delta\vec{\mathbf{k}}) e^{i\theta_{\mathrm{i}}^{(1)}(\vec{\mathbf{k}}^{\mathrm{i}})} +$$

$$\sqrt{\beta} \sum_{\vec{\mathbf{k}}^{\mathrm{i}}} \mathcal{E}_{\mathrm{o}}^{\mathrm{M}}(\vec{\mathbf{k}}^{\mathrm{i}} + \Delta\vec{\mathbf{k}}) e^{i\theta_{\mathrm{i}}^{(1)}(\vec{\mathbf{k}}^{\mathrm{i}})}. \quad (1)$$

For each $\vec{\mathbf{k}}^{\mathrm{i}}$, we identified $\theta_{\mathrm{i}}^{(1)}(\vec{\mathbf{k}}^{\mathrm{i}})$ that maximizes the total intensity of the corrected CLASS image:

$$\max_{\theta_{\mathrm{i}}^{(1)}(\vec{\mathbf{k}}^{\mathrm{i}})} \sum_{\Delta\vec{\mathbf{k}}} \left|\mathcal{E}_{\mathrm{CLASS}}^{(1)}(\Delta\vec{\mathbf{k}})\right|^{2}. \quad (2)$$

The maximum of the total intensity occurs when the sum of the cross-terms between single-scattered waves in Eq. (2) are the largest. This is the case when the following cross-term originating from the two arbitrary incident wavevectors, $\vec{\mathbf{k}}_1^{\mathrm{i}}$ and $\vec{\mathbf{k}}_2^{\mathrm{i}}(\neq \vec{\mathbf{k}}_1^{\mathrm{i}})$, are real valued.

$$\exp i\{\phi_{\mathrm{i}}(\vec{\mathbf{k}}_2^{\mathrm{i}}) - \phi_{\mathrm{i}}(\vec{\mathbf{k}}_1^{\mathrm{i}})\} \exp\left[-i\left\{\theta_{\mathrm{i}}^{(1)}(\vec{\mathbf{k}}_2^{\mathrm{i}}) - \theta_{\mathrm{i}}^{(1)}(\vec{\mathbf{k}}_1^{\mathrm{i}})\right\}\right]$$

$$\cdot \left[\sum_{\Delta\vec{\mathbf{k}}} \{\mathcal{O}(\Delta\vec{\mathbf{k}}) P_{\mathrm{o}}^{\mathrm{a}}(\Delta\vec{\mathbf{k}} + \vec{\mathbf{k}}_1^{\mathrm{i}})\}\{\mathcal{O}(\Delta\vec{\mathbf{k}}) P_{\mathrm{o}}^{\mathrm{a}}(\Delta\vec{k} + \vec{\mathbf{k}}_2^{\mathrm{i}})\}^{*}\right]. \quad (3)$$

Because only relative phase matters, we can set $\theta_{\mathrm{i}}^{(1)}(\vec{\mathbf{k}}_1^{\mathrm{i}} = 0) = 0$ and $\phi_{\mathrm{i}}(\vec{\mathbf{k}}_1^{\mathrm{i}} = 0) = 0$ and $\vec{\mathbf{k}}_2^{\mathrm{i}} = \vec{\mathbf{k}}^{\mathrm{i}}$. Then the maximization operation leads to

$$\theta_{\mathrm{i}}^{(1)}(\vec{\mathbf{k}}^{\mathrm{i}}) = \phi_{\mathrm{i}}(\vec{\mathbf{k}}^{\mathrm{i}}) + \Phi_{\mathrm{i}}^{(1)}(\vec{\mathbf{k}}^{\mathrm{i}}), \quad (4)$$

where $\Phi_{\mathrm{i}}^{(1)}(\vec{\mathbf{k}}^{\mathrm{i}}) = \angle f^{(1)}(\vec{\mathbf{k}}^{\mathrm{i}})$ and $f^{(1)}(\vec{\mathbf{k}}^{\mathrm{i}}) = \sum_{\Delta\vec{\mathbf{k}}}\{\mathcal{O}(\Delta\vec{\mathbf{k}})P_{\mathrm{o}}^{\mathrm{a}}(\Delta\vec{\mathbf{k}})\}\{\mathcal{O}(\Delta\vec{\mathbf{k}})P_{\mathrm{o}}^{\mathrm{a}}(\Delta\vec{\mathbf{k}}+\vec{\mathbf{k}}^{\mathrm{i}})\}^{*} = \{(|\mathcal{O}|^{2}P_{\mathrm{o}}^{\mathrm{a}})\star(P_{\mathrm{o}}^{\mathrm{a}})\}(\vec{\mathbf{k}}^{\mathrm{i}})$. Here $\angle$ stands for the phase angle of the associated complex number. Therefore $\Phi_{\mathrm{i}}^{(1)}(\vec{\mathbf{k}}^{\mathrm{i}})$, which is mainly determined by the autocorrelation of the output pupil function, can be considered the error of aberration correction for the illumination.

Conditions for the convergence of iteration

Let us take a close look at the phase correction error $\Phi^{(1)}(\vec{\mathbf{k}}^{\mathrm{i}})$. If there were no output aberration in the reflection process, i.e. $\phi_{\mathrm{o}}(\vec{\mathbf{k}}^{\mathrm{o}}) = 0$ and $P_{\mathrm{o}}^{\mathrm{a}}(\vec{\mathbf{k}}^{\mathrm{o}}) = P(\vec{\mathbf{k}}^{\mathrm{o}})$, then $\Phi^{(1)}(\vec{\mathbf{k}}^{\mathrm{i}})$ is zero. This means that the aberration in the illumination beam path can be perfectly corrected by just the single run of operation in Eq. (2). In reality, the output aberrations always exist and $\Phi_{\mathrm{i}}^{(1)}(\vec{\mathbf{k}}^{\mathrm{i}}) \neq 0$. Therefore, the presence of output aberrations makes the aberration correction of input imperfect in the coherent imaging. In the worst case when $\Phi_{\mathrm{i}}^{(1)}(\vec{\mathbf{k}}^{\mathrm{i}})$ are randomly distributed between $-\pi$ and π, then the maximization process cannot correct the input aberration at all. In order for our aberration correction approach to be effective and the iterative operation explained in the main text to be convergent, $\Phi_{\mathrm{i}}^{(1)}(\vec{\mathbf{k}}^{\mathrm{i}})$ should not be completely random, but have a finite width of distribution near zero.

There are two requirements that set the convergence condition. First, $\mathcal{O}(\Delta\vec{\mathbf{k}})P_{\mathrm{o}}^{\mathrm{a}}(\Delta\vec{\mathbf{k}})$ needs to be a slowly varying function with respect to $\Delta\vec{\mathbf{k}}$. Then the phase angle of its autocorrelation, $\Phi_{\mathrm{i}}^{(1)}(\vec{\mathbf{k}}^{\mathrm{i}})$, has a finite width of distribution around zero. Even with the severe aberrations assumed in Supplementary Figures 1a and 1b, we can make $\mathcal{O}(\Delta\vec{\mathbf{k}})P_{\mathrm{o}}^{\mathrm{a}}(\Delta\vec{\mathbf{k}})$ a slowly varying function by the fine sampling of individual images in the k-space. This can be done by taking images over a wide view field because the sampling resolution in k-space is determined by the reciprocal of the width of view field. And this is reason why the CLASS microscopy needs the wide-field imaging in the first place.

The second condition that determines the convergence of iteration is the relative intensity of multiple light scattering with respect to that of single scattering. In assessing the cross-term of the single-scattered waves shown in Eq. (3), the cross-term of the multiple-scattered waves described in the second term in Eq. (1) serve as noise. If $\beta/\eta\gamma$ is too large, then the phase of this cross-term will not be finite in width, but uniformly distributed between $-\pi$ and π. See the detailed numerical test of the convergence at Supplementary Note 2.

Closed-loop iteration by the phase-conjugation operation

After identifying $\theta_{\mathrm{i}}^{(1)}(\vec{\mathbf{k}}^{\mathrm{i}})$ that maximizes the total intensity of the CLASS image (Eq. (4)), we inserted it into Eq. (1). This modifies the spectrum of CLASS image to

$$\mathcal{E}_{\mathrm{CLASS}}^{\mathrm{c}}(\Delta\vec{\mathbf{k}}) = \sqrt{\gamma}\mathcal{O}(\Delta\vec{\mathbf{k}}) \cdot \sum_{\vec{\mathbf{k}}^{\mathrm{i}}} P_{\mathrm{i}}^{(1)}(\vec{\mathbf{k}}^{\mathrm{i}}) P_{\mathrm{o}}^{\mathrm{a}}(\vec{\mathbf{k}}^{\mathrm{i}} + \Delta\vec{\mathbf{k}}) + \sqrt{\beta} \sum_{\vec{\mathbf{k}}^{\mathrm{i}}} \mathcal{E}_{\mathrm{o}}^{\mathrm{M}}(\vec{\mathbf{k}}^{\mathrm{i}} + \Delta\vec{\mathbf{k}}) \exp\left[i\theta_{\mathrm{i}}^{(1)}(\vec{\mathbf{k}}^{\mathrm{i}})\right]. \quad (5)$$

Here, the input pupil function $P_{\mathrm{i}}^{\mathrm{a}}(\vec{\mathbf{k}}^{\mathrm{i}})$ is replaced by the modified pupil function,

$$P_{\mathrm{i}}^{(1)}(\vec{\mathbf{k}}^{\mathrm{i}}) = P(\vec{\mathbf{k}}^{\mathrm{i}}) \cdot \exp\left[-i\phi_{\mathrm{i}}(\vec{\mathbf{k}}^{\mathrm{i}}) + i\theta_{\mathrm{i}}^{(1)}(\vec{\mathbf{k}}^{\mathrm{i}})\right] = P(\vec{\mathbf{k}}^{\mathrm{i}}) \cdot \exp\left[i\Phi_{\mathrm{i}}^{(1)}(\vec{\mathbf{k}}^{\mathrm{i}})\right]. \quad (6)$$

Therefore, finding $\theta_{\mathrm{i}}(\vec{\mathbf{k}}^{\mathrm{i}})$ that maximizes the total intensity of CLASS image is identical to replacing input aberration $\phi_{\mathrm{i}}(\vec{\mathbf{k}}^{\mathrm{i}})$ with $\Phi_{\mathrm{i}}^{(1)}(\vec{\mathbf{k}}^{\mathrm{i}})$. Under the convergence conditions discussed in 1.2, the uniform random phase distribution of initial aberrations is reduced to the finite width of the distribution of $\Phi^{(1)}(\vec{\mathbf{k}}^{\mathrm{i}})$. However, there will not be a further reduction without additional independent operation to correct the sample-induced aberrations in the collection process. In order to form a closed-loop correction, we considered a phase conjugation process in which the wave is incident from $-\vec{\mathbf{k}}^{\mathrm{o}}$ and reflected to $-\vec{\mathbf{k}}^{\mathrm{i}} = -(\vec{\mathbf{k}}^{\mathrm{o}} - \Delta\vec{\mathbf{k}})$. With this reverse process, the CLASS spectrum after the initial correction in Eq. (5) can be written as

$$\mathcal{E}_{\text{CLASS}}^{\text{pc}}(\Delta\vec{\mathbf{k}}) = \sqrt{\gamma}\mathcal{O}^{-1}(\Delta\vec{\mathbf{k}}) \cdot \sum_{\vec{\mathbf{k}}^{\text{o}}} P_{\text{o}}^{\text{a}}(\vec{\mathbf{k}}^{\text{o}})^{*} P_{\text{i}}^{(1)}(\vec{\mathbf{k}}^{\text{o}} - \Delta\vec{\mathbf{k}})^{*} \exp i\theta_{\text{o}}^{(1)}(\vec{\mathbf{k}}^{\text{o}})$$

$$+ \sqrt{\beta} \sum_{\vec{\mathbf{k}}^{\text{o}}} \mathcal{E}_{\text{o}}^{\text{M}}(\vec{\mathbf{k}}^{\text{o}} - \Delta\vec{\mathbf{k}})^{*} \exp\left[i\theta_{\text{i}}^{(1)}(\vec{\mathbf{k}}^{\text{i}})\right] \exp i\theta_{\text{o}}^{(1)}(\vec{\mathbf{k}}^{\text{o}}), \quad (7)$$

where $\mathcal{O}^{-1}$ is inverse convolution of object transfer function, such that $\mathcal{O}^{-1} * \mathcal{O} = \delta$. Note that the summation operation is now performed over $\vec{\mathbf{k}}^{\text{o}}$. Similar to the correction of illumination, we add angle-dependent phase correction $\theta_{\text{o}}(\vec{\mathbf{k}}^{\text{o}})$, and then maximized the total intensity of the phase-conjugated CLASS image:

$$\max_{\theta_{\text{o}}^{(1)}(\vec{\mathbf{k}}^{\text{o}})} \sum_{\Delta\vec{\mathbf{k}}} \left|\mathcal{E}_{\text{CLASS}}^{\text{pc}}(\Delta\vec{\mathbf{k}})\right|^{2}. \quad (8)$$

Similar to the illumination correction, $\theta_{\text{o}}^{(1)}(\vec{\mathbf{k}}^{\text{o}})$ is determined as

$$\theta_{\text{o}}^{(1)}(\vec{\mathbf{k}}^{\text{o}}) = \phi_{\text{o}}(\vec{\mathbf{k}}^{\text{o}}) + \Phi_{\text{o}}^{(1)}(\vec{\mathbf{k}}^{\text{o}}) , \quad (9)$$

with $\Phi_{\text{i}}^{(1)}(\vec{\mathbf{k}}^{\text{o}}) = \angle g^{(1)}(\vec{\mathbf{k}}^{\text{o}})$ and $g^{(1)}(\vec{\mathbf{k}}^{\text{o}}) = \left\{\left(|\mathcal{O}^{-1}|^{2} P_{\text{i}}^{(1)^{*}}\right) \star \left(P_{\text{i}}^{(1)^{*}}\right)\right\}(\vec{\mathbf{k}}^{\text{o}})$.

And this output correction replaces the output pupil function with

$$P_{\text{o}}^{(1)}(\vec{\mathbf{k}}^{\text{o}}) = P(\vec{\mathbf{k}}^{\text{o}})\exp\left[-i\phi_{\text{o}}(\vec{\mathbf{k}}^{\text{o}}) + i\theta_{\text{o}}^{(1)}(\vec{\mathbf{k}}^{\text{o}})\right] = P(\vec{\mathbf{k}}^{\text{o}})\exp\left[i\Phi_{\text{o}}^{(1)}(\vec{\mathbf{k}}^{\text{o}})\right]. \quad (10)$$

This is equivalent to replacing output aberration $\phi_{\text{o}}(\vec{\mathbf{k}}^{\text{o}})$ with $\Phi_{\text{o}}^{(1)}(\vec{\mathbf{k}}^{\text{o}})$. Note that the $\Phi_{\text{o}}^{(1)}(\vec{\mathbf{k}}^{\text{o}})$ is even more narrowly peaked around zero than $\Phi_{\text{i}}^{(1)}(\vec{\mathbf{k}}^{\text{i}})$ because the input correction $\theta_{\text{i}}(\vec{\mathbf{k}}^{\text{i}})$ has converted P_{i}^{a} to a more slowly varying function $P_{\text{i}}^{(1)}$. As a consequence, the error of the first round of output aberration correction is smaller than that of the illumination correction.

With the first round of input and output corrections in place, the second round of input and output corrections, $\theta_{\text{i}}^{(2)}(\vec{\mathbf{k}}^{\text{i}})$ and $\theta_{\text{o}}^{(2)}(\vec{\mathbf{k}}^{\text{o}})$ respectively, are applied to progressively reduce the residual error of the aberration corrections. And the iteration keeps going until the residual error is made

below certain level, which is typically set as 0.1 radians. We can find the recursion relation for both input and output pupil functions after n iterations.

$$P_{\mathrm{i}}^{(n)} = P \cdot \frac{\left(|\mathcal{O}|^2 \cdot P_{\mathrm{o}}^{(n-1)}\right) \star P_{\mathrm{o}}^{(n-1)}}{\left|\left(|\mathcal{O}|^2 \cdot P_{\mathrm{o}}^{(n-1)}\right) \star P_{\mathrm{o}}^{(n-1)}\right|}. \qquad (11)$$

$$P_{\mathrm{o}}^{(n)} = P \cdot \frac{\left(|\mathcal{O}^{-1}|^2 \cdot P_{\mathrm{i}}^{*(n)}\right) \star P_{\mathrm{i}}^{*(n)}}{\left|\left(|\mathcal{O}^{-1}|^2 \cdot P_{\mathrm{i}}^{*(n)}\right) \star P_{\mathrm{i}}^{*(n)}\right|}. \qquad (12)$$

with initial condition $P_{\mathrm{i}}^{(0)} = P_{\mathrm{i}}^{\mathrm{a}}$, and $P_{\mathrm{o}}^{(0)} = P_{\mathrm{o}}^{\mathrm{a}}$.

As the iteration number n is increased, the phase angles $\Phi_{\mathrm{i}}^{(n)}(\vec{\mathbf{k}}^{\mathrm{i}})$ and $\Phi_{\mathrm{o}}^{(n)}(\vec{\mathbf{k}}^{\mathrm{o}})$ converge to zero on the conditions described in 1.2. Consequently, the pupil functions for the input and output in Eqs. (11) and (12) converge to the ideal pupil function $P(\vec{\mathbf{k}})$.

After all, we can find the initial aberrations by accumulating phase corrections identified in all the iterations:

$$\phi_{\mathrm{i}}(\vec{\mathbf{k}}^{\mathrm{i}}) = \sum_n \theta_{\mathrm{i}}^{(n)}(\vec{\mathbf{k}}^{\mathrm{i}}), \;\; \phi_{\mathrm{o}}(\vec{\mathbf{k}}^{\mathrm{o}}) = \sum_n \theta_{\mathrm{o}}^{(n)}(\vec{\mathbf{k}}^{\mathrm{o}}). \qquad (13)$$

Note that the complex conjugation operation is not critical from the mathematical point of view in the second step of CLASS algorithm. It doesn't make any difference in the result except for the sign of the output phase correction map. What is important is the transpose operation, which enables us to swap input and output, and to correct the specimen-induced aberration from the output side. The reason we take phase conjugation (transpose + complex conjugation) rather than just the transpose is to impose a physical meaning to the operation. In this way, the second step is equivalent to physically sending the waves from the output side and measuring the backscattered waves at the input side.

Supplementary Note 2. Numerical simulation study

For the complete understanding of the effect of CLASS algorithm on correcting the aberrations of single-scattered waves and attenuating the contribution of multiple light scattering, we performed a numerical simulation study by assuming a representative example of sample-induced aberrations.

Applying CLASS algorithm to the numerically prepared aberrations

We set up a numerical simulation at the condition of $\beta = 20\gamma$ and $N_{\mathrm{m}} = 1{,}245$, which corresponds to the number of free modes for a 20×20 μm^2 field of view. Also, we introduced arbitrary aberrations $\phi_{\mathrm{i}}(\vec{\mathbf{k}}^{\mathrm{i}})$ and $\phi_{\mathrm{o}}(\vec{\mathbf{k}}^{\mathrm{o}})$ as depicted in Supplementary Figures 1a and 1b, respectively, which led to $\eta = 1/400$ and $S = 1/3600$. The amplitude of the cross-correlation map of these two complex pupil functions (Supplementary Figure 1c) was well below unity, suggesting that the accumulation of single scattering would be compromised. Supplementary Figures 1d and 1e show CASS images without and with aberrations, respectively, in the absence of multiple scattering. The target objects were a pair of point particles separated by 600 nm, which corresponds to the diffraction-limit resolution for 0.8 NA at the source wavelength λ = 800 nm. As expected, the aberrations made the two particles completely indistinguishable. Supplementary Figures 1f and 1g show CASS images without and with aberrations, respectively, but this time in the presence of multiple scattering. As long as there is no aberration, CASS microscopy works well even if there is strong multiple scattering (Supplementary Figure 1f). The simultaneous presence of scattering and aberration (Supplementary Figure 1g) makes it even more difficult to resolve the two particles than in the aberration-only case because, in addition to being improperly accumulated, the single-scattered waves are concealed by the multiple-scattered waves.

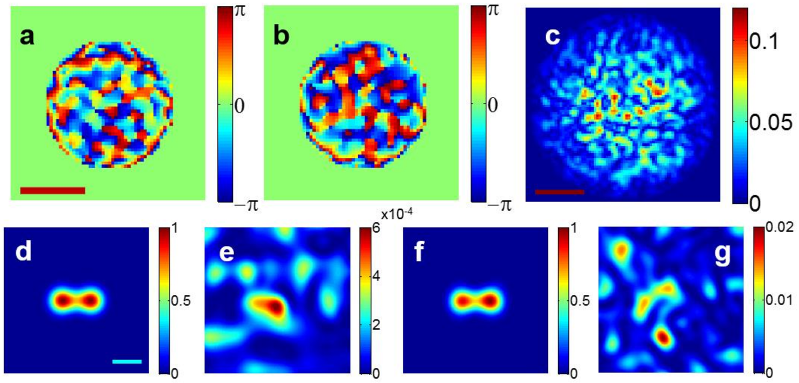

Supplementary Figure 1. The effect of sample-induced aberration in imaging targets inside a thick scattering medium. a and **b**, Angle-dependent phase retardations simulated for incident and reflected waves, respectively. For the illumination and reflection processes, random phase shifts were added to the average spherical aberration induced by a 1 mm-thick tissue (refractive index n~1.37) in water. Circular central region corresponds to the pupil of the objective lens with numerical aperture of 0.8. **c**, Amplitude transfer function of CASS microscopy obtained by cross-correlation between the input and output aberrations shown in **a** and **b**. Color scale indicates amplitude transmittance. Scale bars in **a** and **c** correspond to $k_0\alpha$. **d-g**, CASS images of two point particles separated by 600 nm, the diffraction limit of the model system. **d**: without either aberration or multiple scattering. **e**: in the presence of the aberrations shown in **a** and **b**, but with no multiple scattering. **f**: in the presence of multiple scattering but with no aberration. **g**: in the presence of both aberration and scattering. The mean intensity of the multiple scattering was set 200 times stronger than that of single scattering. Color scales in **d-g** are normalized by the peak value in **d**.

We confirmed the effectiveness of CLASS algorithm for the aberrations considered in Supplementary Figures 1a and 1b. The first image in Supplementary Figure 2a shows that $\theta_{\mathrm{i}} = \theta_{\mathrm{i}}^{(1)}$ after the first round of the maximization process. The $\theta_{\mathrm{i}}^{(1)}$ resembles the input aberration shown in Supplementary Figure 1a with a correlation value of 35 %. The first image in Supplementary Figure 2b shows the CLASS image reconstructed after applying this phase correction. The existence of particles becomes better visualized than before. However, the resolving power has not been sufficiently recovered to distinguish the two particles because the output aberration has not yet been addressed.

This first round of maximization operation is incomplete because only the aberration arising from the incident wave can be dealt with. With the correction $\theta_{\mathrm{i}} = \theta_{\mathrm{i}}^{(1)}$ in place, we apply the phase correction to the output and identified the $\theta_{\mathrm{o}} = \theta_{\mathrm{o}}^{(1)}$ that would maximize the total intensity of the phase-conjugated CLASS image. Similar to the correction for the illumination path, this iteration leads to the convergence of $\theta_{\mathrm{o}}^{(1)}(\vec{\mathbf{k}}^{\mathrm{o}})$ to $\phi_{\mathrm{o}}(\vec{\mathbf{k}}^{\mathrm{o}})$. In fact, this correction for the reflection process converges faster than that of $\theta_{\mathrm{i}}^{(1)}$ to $\phi_{\mathrm{i}}(\vec{\mathbf{k}}^{\mathrm{i}})$. Because of the prior correction $\theta_{\mathrm{i}}^{(1)}$, the width of the phase histogram of $\Phi_{\mathrm{o}}^{(1)}(\vec{\mathbf{k}}^{\mathrm{o}})$ is narrower than that of $\Phi_{\mathrm{i}}^{(1)}(\vec{\mathbf{k}}^{\mathrm{i}})$ (see Supplementary Figure 3).

The second image in Supplementary Figure 2a shows the $\theta_{\mathrm{o}}^{(1)}$ identified through this phase-conjugation process, and it shows good correlation with the original aberration map in Supplementary Figure 1b with a correlation value of about 66 %. After this first round of illumination and reflection corrections, the reconstructed CLASS image shown in the second image of Supplementary Figure 2b now resolves the two particles better than before. Since the identified aberration maps are not yet complete, we iterated the aberration correction to improve its accuracy. We observed that the accumulated phase corrections converge to the system aberrations as the number of iterations *n* is increased, i.e. $\sum_n \theta_{\mathrm{i}}^{(n)}(\vec{\mathbf{k}}^{\mathrm{i}}) \rightarrow \phi_{\mathrm{i}}(\vec{\mathbf{k}}^{\mathrm{i}})$ and $\sum_n \theta_{\mathrm{o}}^{(n)} \rightarrow \phi_{\mathrm{o}}(\vec{\mathbf{k}}^{\mathrm{o}})$ (Supplementary Figure 2a). For this example, 3 rounds of iteration led to the determination of the input and output aberrations to an accuracy of 97 %. The reconstructed image became almost the same as the ideal image shown in Supplementary Figure 1d. The resulting CLASS images show the increase in the signal intensity, suggesting that the cross-correlation of the aberration-corrected pupil functions had been increased in magnitude. Taken

together, these observations confirm that the proposed method works extremely well, even in the presence of strong multiple-scattered waves.

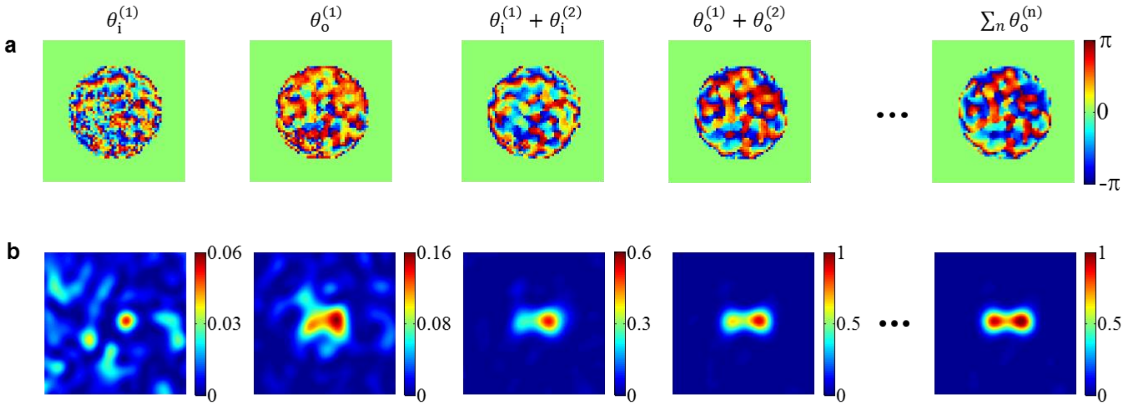

Supplementary Figure 2. Improvement of resolving power with the iterations of CLASS algorithm. Aberrations and the intensity ratio of single- to multiple-scattered waves are the same as those considered in Supplementary Figure 1. **a**, Angle-dependent phase corrections $\theta_i^{(n)}$ and $\theta_o^{(n)}$ as the iteration number n is increased. Scale bar corresponds to $k_0\alpha$. Color bar, phase in radians. **b**, Reconstructed CLASS images when $\theta_i^{(n)}$ and $\theta_o^{(n)}$ in **a** were applied. Color bar, intensity normalized by the peak value of the reconstructed image obtained at the end of iteration. Total number of iterations was 6 for the presented data.

Reduction of phase correction error with the increase of iteration number

In this section, we present the numerical simulation data that visualizes the way the iterative maximization operations (Eqs. (2) and (8)) reduce the phase correction error. In Supplementary Figures 3a and 3c, the phase maps of the modified input and output pupil functions, $\Phi_i^{(n)}$ and $\Phi_o^{(n)}$, were shown respectively for the same numerical data used in Supplementary Figure 1. Initially (n=0), we assumed severe aberrations enough to make the phase of the complex pupil functions, $\Phi_i^{(0)}$ and $\Phi_o^{(0)}$, randomly distributed between $-\pi$ and π (Supplementary Figures 3b and 3d). After the first round of iteration process, the phase maps of input and output pupil functions were replaced by $\Phi_i^{(1)}$ and $\Phi_o^{(1)}$, respectively. As a consequence, the phase maps

shown in Supplementary Figures 3a and 3c ($n = 1$) were smoothened and the width of phase distribution shown in Supplementary Figures 3b and 3d ($n = 1$) were narrowed down. The $\theta_{\mathrm{i}}^{(1)}$ and $\theta_{\mathrm{o}}^{(1)}$ applied to this reconstruction were shown in Supplementary Figure 2a, where they were shown to resemble the aberrations assumed in the beginning.

With the increase of n, the phase distribution of the modified pupil functions became almost constant across the pupils and the widths of the phase distributions were progressively narrowed down. Indeed, the iteration process almost completely counteracted the aberration, thereby converting initial complex pupil functions into aberration-free ones. Our approach is so effective that only a few steps of iterations were good enough to identify the aberrations. At the end of iteration, the sum of phase corrections converged to the initial aberrations (Supplementary Figure 2a).

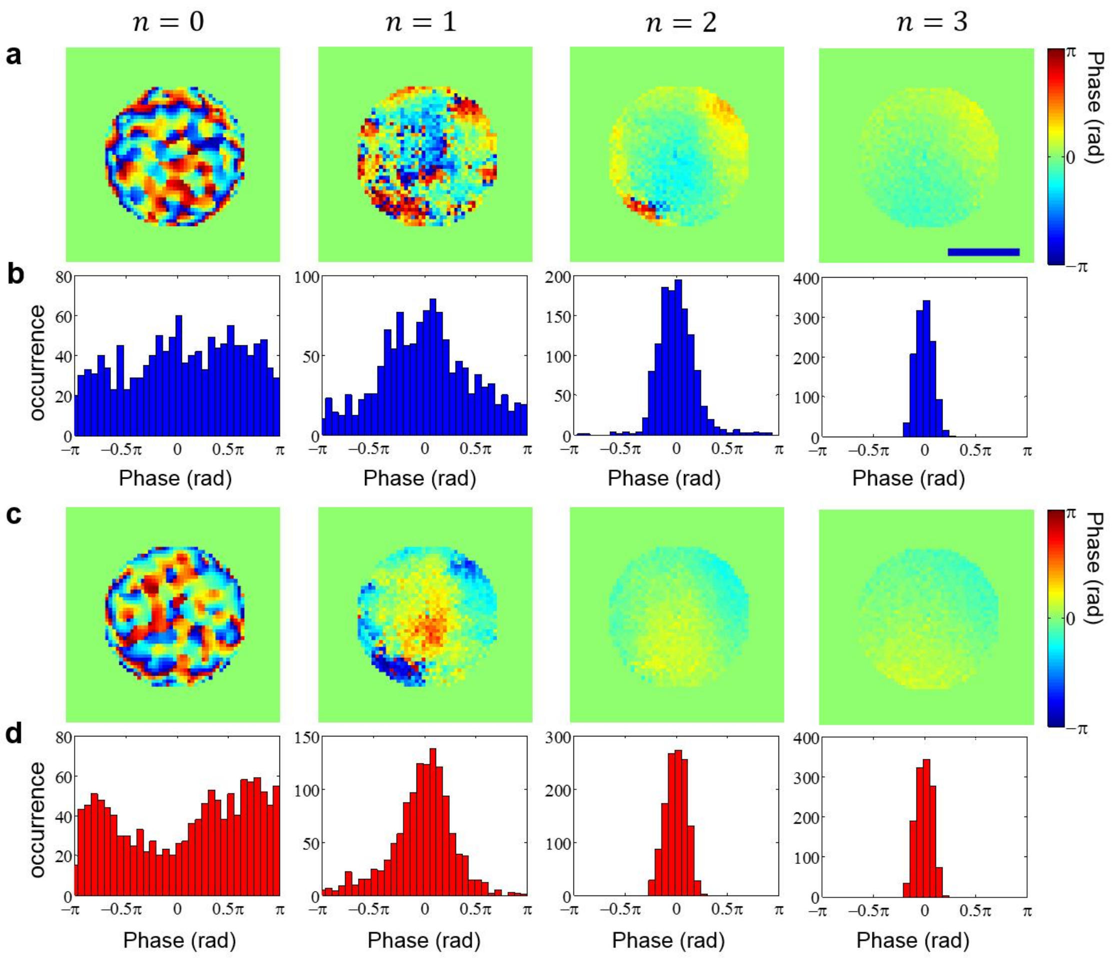

Supplementary Figure 3. Phase distribution of the modified pupil functions with the increase of iteration number. a and **c,** Phase maps of the modified input and output pupil functions, $\Phi_{\mathrm{i}}^{(n)}(\vec{\mathbf{k}}^{\mathrm{i}})$ and $\Phi_{\mathrm{o}}^{(n)}(\vec{\mathbf{k}}^{\mathrm{o}})$, respectively, depending on the iteration number n. Color bar, phase in radians. Scale bar, $k_0\alpha$. **b** and **d,** Histograms of phase angles displayed in **a** and **c**, respectively.

In order to quantitatively assess the effectiveness of convergence, we computed the correlation between the original aberrations and the aberration correction maps identified from CLASS algorithm. To this end, the correlation between the obtained aberration maps and the initial aberration maps in Supplementary Figures 1a and 1b was computed for each iteration number. As shown in Supplementary Figure 4, only 3 iterations were good enough to recover the initial aberration map with the accuracy of more than 97%. Note that the output correlation for the nth

iteration step is always larger than n^{th} input correlation because the nth output correction was performed after n^{th} input correction.

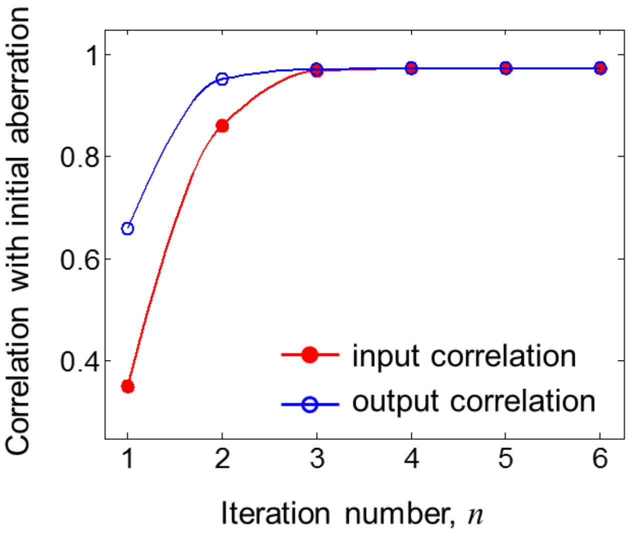

Supplementary Figure 4. Correlation between obtained aberration map and initial aberration for the input (red curve) and output (blue curve) aberrations.

The effect of aberration corrections to the transfer function of CLASS image

By Eqs. (1), (6), and (10), the spectrum of single-scattered waves in CLASS image after n times of input-output phase corrections can be written as

$$\mathcal{E}_{\text{CLASS}}^{(n)}(\Delta\vec{\mathbf{k}}) = \sqrt{\gamma}\mathcal{O}(\Delta\vec{\mathbf{k}}) \cdot \left(P_{\text{i}}^{*(n)} \star P_{\text{o}}^{(n)}\right)(\Delta\vec{\mathbf{k}}). \qquad (14)$$

The amplitude transfer function (ATF) of CLASS image is modified to $\mathcal{A}^{(n)} = P_{\text{i}}^{*(n)} \star P_{\text{o}}^{(n)}$ after n^{th} round of iteration process. The width and height of ATF are related to the spatial resolution and signal strength of single-scattered waves, respectively. In Supplementary Figure 5, we present $\mathcal{A}^{(n)}$ for various n for the numerical simulation data. As expected, $\mathcal{A}^{(n)}$ converged to the ideal ATF without aberration as n was increased. The width of ATF was broadened to that of the ideal ATF, indicating that the diffraction-limited spatial resolution was almost recovered.

More importantly, the average height of ATF was increased by about 20 times after the correction, which corresponds to the increase of the intensity of single-scattered waves by about 400 times. This is critical in recovering single-scattering signals when there exists strong multiple-scattering background. Indeed, our correction method not only recovers spatial resolution, but also enhances the signal strength of single-scattered waves.

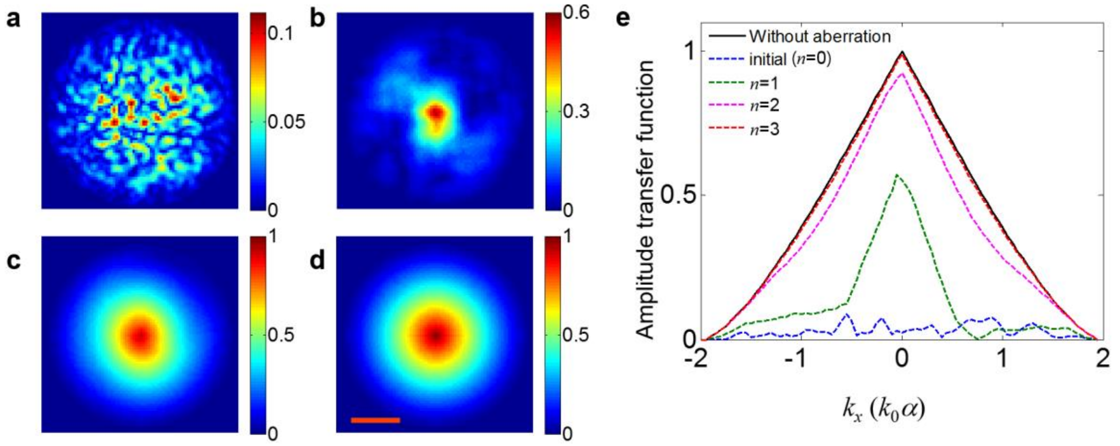

Supplementary Figure 5. Amplitude transfer function of single-scattered waves with the increase of iteration number. a-d, Amplitude transfer functions of CLASS microscopy for n=0, 1, 2, 3, respectively. Scale bar, $k_0\alpha$. **e,** Dashed lines: line profiles of ATFs shown in **a-d** at $k_y = 0$. Solid line: ATF of an ideal pupil function. The plots were normalized by the maximum value of the ideal ATF.

The intensity of single- and multiple-scattered waves with the increase of iteration number

Since our aberration correction method is based on enhancing the coherent summation of single-scattered waves, the total intensity of CLASS image should increase by the iteration process. In the simulation data, we can separate out single- and multiple-scattered waves in the CLASS image. Therefore, we could analyze the way the correction process affects to the intensities of single- and multiple-scattered waves. Supplementary Figure 6 shows the intensities of the single- and multiple-scattered waves as well as the total intensity of the CLASS image as a function of the iteration number. Initially, multiple-scattered waves was about 20 times larger than the single

scattered waves because of the strong aberration. Total intensity of CLASS image was almost equal to that of multiple-scattered waves, and this is the reason the target was initially invisible. When the iteration number was increased, the intensity of single-scattered waves increased significantly by up to about 400 times while that of the multiple-scattered waves didn't change much. As a result, the intensity ratio between single- and multiple-scattered waves was reversed, and the contribution of single-scattered waves became dominant in the CLASS image. The led to the clear identification of targets as shown in the last image of Supplementary Figure 2b.

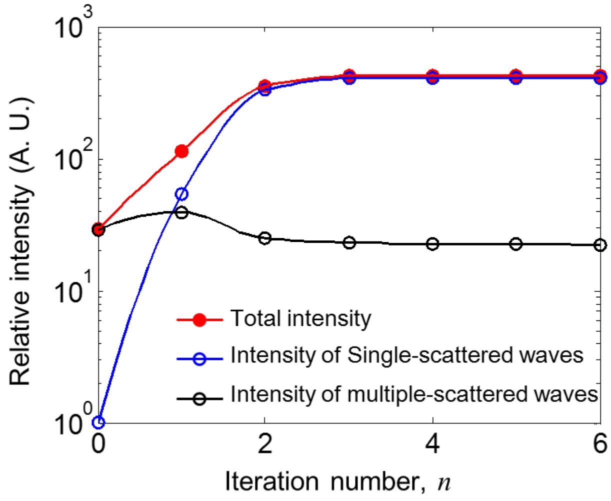

Supplementary Figure 6. The effect of aberration correction to the intensities of single- and multiple-scattered waves in the reconstructed CLASS images. Red, blue, black markers correspond to the total intensity of CLASS image, the intensity of single-scattered waves, and that of the multiple-scattered waves, respectively. Plots were normalized by the initial contribution of single-scattered waves.

Relation between η and Strehl ratio S

In adaptive optics, the degree of aberration is measured by Strehl ratio, which is the peak intensity of the point spread function with aberration relative to that without aberration. For the case when both the input and output aberration matter, this parameter can be described by the amplitude transfer function,

$$S = \left|\langle \mathcal{A}(\vec{\mathbf{k}}) \rangle\right|^2 / \left|\langle \mathcal{A}_0(\vec{\mathbf{k}}) \rangle\right|^2 . \qquad (15)$$

Here $\mathcal{A} = P_{\mathrm{i}}^{\mathrm{a}\,*} \star P_{\mathrm{o}}^{\mathrm{a}}$, and $\mathcal{A}_0(\vec{\mathbf{k}})$ is the ideal amplitude transfer function. For a relatively mild aberration and weak multiple scattering noise, S faithfully describes the effect of aberrations to the signal strength and image contrast. But it's use is limited in case of severe aberration or strong multiple scattering noise. In our study, we introduced the parameter η in Eq. (3), which is the ratio of the total intensity of single-scattered waves in the presence of aberration with respect to that in the absence of aberration. In terms of the amplitude transfer function, η can be simplified to

$$\eta = \langle |\mathcal{A}(\vec{\mathbf{k}})|^2 \rangle / \langle |\mathcal{A}_0(\vec{\mathbf{k}})|^2 \rangle \,. \tag{16}$$

In other words, η is the ratio of the total intensity of the point spread function with aberration to that without aberration. As described in the main text, η is responsible for the degradation of the signal to noise ratio of CASS microscopy from $(\gamma/\beta)N_{\mathrm{m}}$ to $\eta \times (\gamma/\beta)N_{\mathrm{m}}$. And the main aim of CLASS microscopy is to increase η enough to raise the signal to noise ratio above unity. And the initial η will set the limit that the CLASS microscopy can work.

The S and η are related to a certain extent. The increase of aberration accompanies the simultaneous reduction in the peak height and the total intensity of the point-spread-function. In general, the peak height is more susceptible than the total intensity to the aberration. Therefore, S is usually smaller than η. But their relation depends on the type of aberration, making it difficult to find a general relation between them. For a heuristic purpose, here we gave an example for the aberration maps assumed in Supplementary Figure 1. As a function of the standard deviation of the phase in the aberration, we plotted both S and η in Supplementary Figure 7. When the deviation of phase shift is not large enough, S and η have almost the same values. However, as the standard deviation of aberration phase increases, S decreases faster than η as mentioned above.

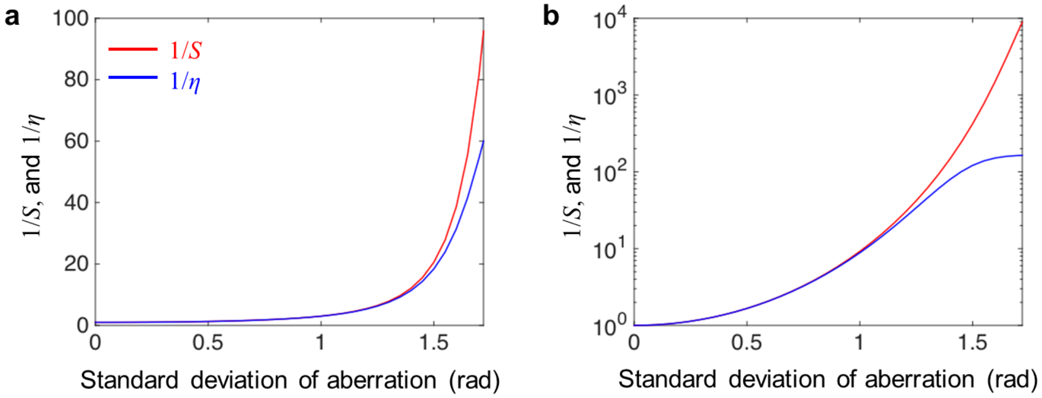

Supplementary Figure 7. Relation between the Strehl ratio S, and η as a function of the standard deviation of phase retardation by the aberration. a, Only the input aberration is accounted for. **b,** Both the input and output aberrations are taken into account. Red line: $1/S$. Blue line: $1/\eta$.

Let us make a further discuss the physical meaning of S and η. S is a parameter used to describe the aberration-induced signal reduction in the confocal and two-photon microscopes. It is a directly relevant parameter to the general adaptive optics audience because it indicates the degree of aberrations of the samples used in our study.

On the other hand, η describes the reduction of single-scattering intensity in the CLASS microscopy where images are acquired over wide area. Unlike confocal microscopy where signal is collected only at the confocal pinhole, the entire PSF contributes to the signal intensity in the wide-field detection. Therefore, the degradation of SNR in the CLASS microscope is determined by η, not by S. Likewise, the main role of CLASS algorithm is to increase single scattering intensity by $1/\eta$, not by $1/S$.

The effect of multiple light scattering to the convergence of CLASS algorithm

As we discussed in section 1, the second condition that determines the convergence of iteration is the relative intensity of multiple light scattering with respect to that of single scattering. In assessing the cross-term of the single-scattered waves shown in Eq. (3), the cross-term of the multiple-scattered waves described in the second term in Eq. (1) serve as noise. If $\beta/\eta\gamma$ is too

large, then the phase of this cross-term will not be finite in width, but uniformly distributed between $-\pi$ and π. This can be seen in Supplementary Figures 8a-d in which the width of distribution was gradually increased as $\beta/\eta\gamma$ was increased. But the convergence of the iteration was guaranteed even when $\beta/\eta\gamma$~16,000.

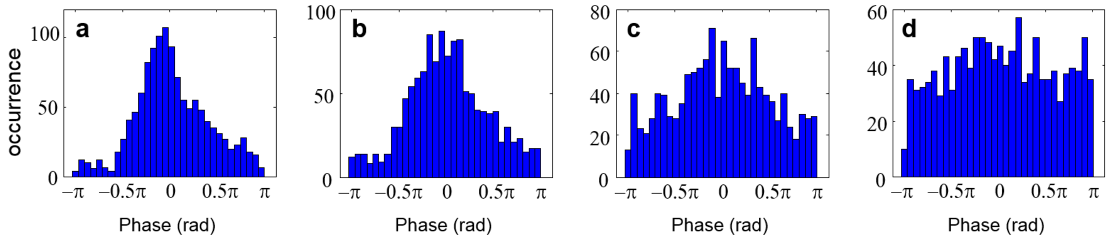

Supplementary Figure 8. Effect of multiple scattering on the phase correction error, $\Phi_{\mathrm{i}}^{(1)}(\vec{\mathbf{k}}^{\mathrm{i}})$**.** The histogram of $\Phi_{\mathrm{i}}^{(1)}(\vec{\mathbf{k}}^{\mathrm{i}})$ when the relative intensity of multiple- to single-scattered waves increases. **a-d**, Intensity ratio between multiple- to single-scattered waves, β/γ are 0, 20, 40, and 45, respectively and $\eta \sim 1/400$. Therefore, the initial intensity ratio of the multiple- to single-scattered waves, $\beta/\eta\gamma$, are 280, 8000, 16,000, and 17,550, respectively. Up to $\beta/\eta\gamma \leq 16{,}000$, the histogram has a finite width such that we could successfully characterize the input and output aberration.

Supplementary Note 3. Detailed experimental setup and additional analysis of experimental data

Experimental setup

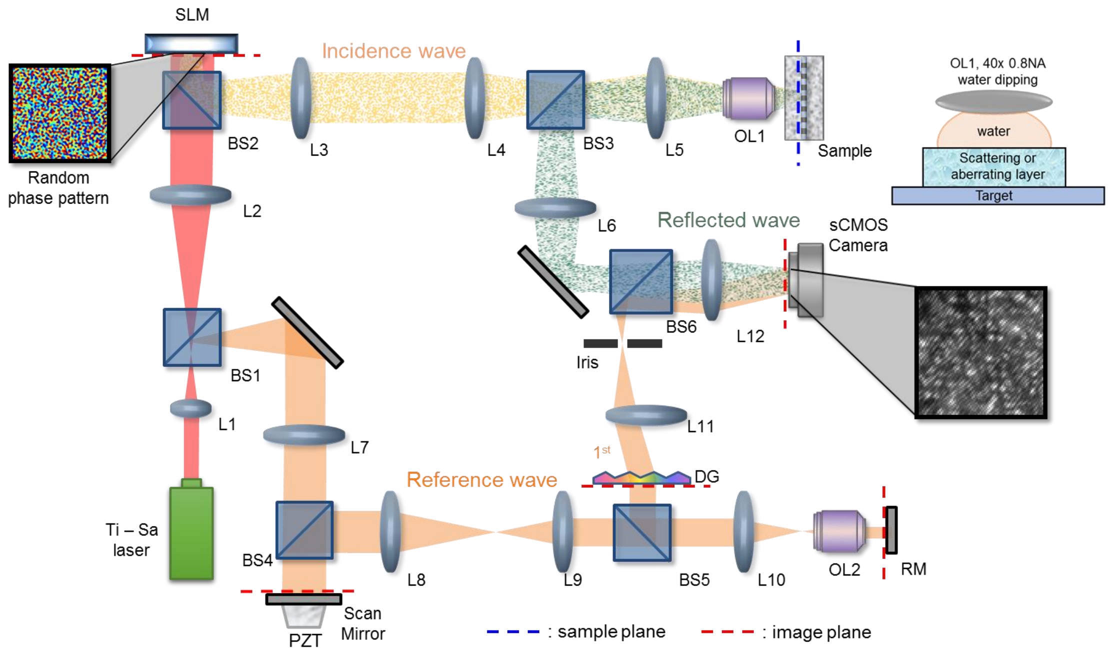

Supplementary Figure 9. Schematic diagram of the experimental setup. L: lens, BS: beam splitter, OL: objective lens, SLM: spatial light modulator, DG: diffraction grating. Inset on the left of SLM shows typical random phase pattern written on the SLM. Inset on the right of sCMOS camera shows the resulting raw data obtained by the camera. The layout of the sample geometry is shown in the upper right.

Detailed experimental setup of CLASS microscopy is shown in Supplementary Figure 9. The basic layout of the setup is identical to the CASS microscopy that we previously reported[1]. However, light source, objective lens and camera were upgraded, and random phase patterns were used for the illumination. The backbone of the setup was a Mach-Zehnder interferometer, and low coherence light source and off-axis geometry were used for the time-resolved and wide-field complex-field imaging.

A femtosecond laser pulse from mode-locked Ti-Sapphire laser (center wavelength 800 nm, bandwidth 30 nm, and pulse width 100 fs) was divided into incidence and reference waves at BS1. The transmitted light from BS1 was delivered to the spatial light modulator (SLM, Hamamatsu X10468-02). By writing random phase patterns on the SLM, we generated random speckle fields

and use them as incidence beams to the sample (inset on the left of the SLM). By two 4-f imaging systems formed by L3, L4, L5, and OL1 (objective lens, Nikon CFI-Apo-40X-W-NIR, 0.8 NA water dipping), we delivered the image of the speckled incidence beam into the sample plane. As shown in the upper right of the figure, we used water as an index matching medium to minimize the refractive index mismatch between the medium and the biological tissue. The reflection from the sample was collected by the same objective lens, and then delivered to the camera (pco.edge 4.2 scientific CMOS camera) placed on the conjugate image plane of the sample plane.

The reference wave reflected at BS1 traveled through similar optics as the sample wave. A scanning mirror was placed in the reference beam path to tune the path length of reference wave. To generate an off-axis interferogram, we selected the 1st order diffracted beam from a diffraction grating (Ronchi ruling, 72 lp/mm, Edmund optics) placed on the conjugate image plane of the camera. At BS6, the reference wave was combined with the reflected wave from the sample. Inset on the right of the camera in Supplementary Figure 9 shows the typical image of the interference pattern. Stripe patterns appearing on the speckled pattern of the sample wave were due to this off-axis interference. By applying Hilbert transform on the measured interferogram, we could obtain the time-gated complex field map of the reflected wave from the sample.

To construct a time-gated reflection matrix, typically 2,800 complex images were taken for the pre-determined set of random patterns of illumination written on the SLM. The number of images was determined in such a way to cover the maximum number of orthogonal channels for $30 \times 30\ \mu\text{m}^2$ view field with the collection angle of 0.8 NA.

Measurement of CLASS image using random basis.

As discussed in the online Methods, we recorded a time-resolved complex field map $u_\text{i}(\vec{\mathbf{r}}_\text{i}; j, \tau_0)$ for the jth random pattern written on the SLM by placing an ideal mirror at the sample plane for the arrival time τ_0 associated with the depth of the mirror. The representative complex-field

maps shown in Supplementary Figure 10a exhibited speckle patterns due to the use of random patterns of illumination. For the same set of illumination patterns, the complex-field map $u_{\mathrm{o}}(\vec{\mathbf{r}}_{\mathrm{o}}; j, \tau_0)$ of the reflected waves was recorded by placing the scattering and the aberrating medium on the test target as shown in Fig. 1b.

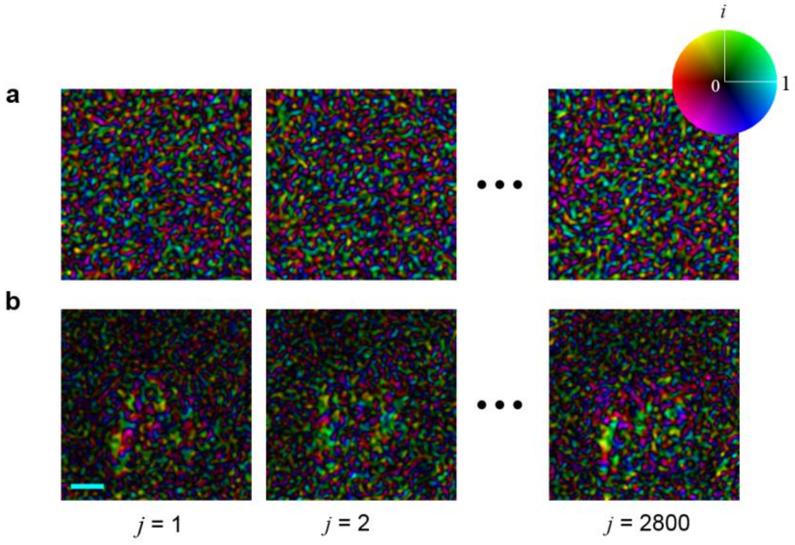

Supplementary Figure 10. Recording of complex field maps for random basis of illumination. a, Complex field maps of the incident waves generated by writing random phase patterns on the SLM. Images were acquired by placing a clean mirror at the sample stage. Only a few representative images from 2,800 are shown. **b,** Complex field maps taken for the sample shown in Fig. 1b for the same set of illumination patterns used in **a**. Scale bar, 5μm. Images in **a** and **b** are normalized by their respective maximum amplitudes. The color map indicates both the amplitude and phase of the complex field.

The use of random basis enabled us to distinguish the uncontrolled phase shifts of interferometric detection induced by the fluctuation of the relative beam path between sample and reference waves from the angle-dependent aberrations by the sample itself. In this case, we applied for an additional phase correction step to deal with the uncontrolled phase shifts among measurements for different speckled illuminations. To this end, we constructed the time-gated reflection matrix on the momentum difference domain, $\mathcal{E}_{\mathrm{o}}(\vec{\mathbf{k}}^{\mathrm{o}} - \vec{\mathbf{k}}^{\mathrm{i}}; \vec{\mathbf{k}}^{\mathrm{i}})$ as discussed in the method section. By

multiplying the input basis matrix, $\mathcal{U}_{\rm i}(\vec{\mathbf{k}}^{\rm i};j)$, the input plane wave basis was converted into the random speckled illumination basis,

$$\mathcal{E}_{\rm o}(\vec{\mathbf{k}}^{\rm o}-\vec{\mathbf{k}}^{\rm i};j)=\mathcal{E}_{\rm o}(\vec{\mathbf{k}}^{\rm o}-\vec{\mathbf{k}}^{\rm i};\vec{\mathbf{k}}^{\rm i})\times\mathcal{U}_{\rm i}(\vec{\mathbf{k}}^{\rm i};j). \quad (17)$$

The individual columns in $\mathcal{E}_{\rm o}(\vec{\mathbf{k}}^{\rm o}-\vec{\mathbf{k}}^{\rm i};j)$ correspond to the object spectrums in the momentum difference for the illumination of jth speckled pattern. Therefore, the summation of the matrix along row direction (i.e. summation of elements with the same $\Delta\vec{\mathbf{k}}=\vec{\mathbf{k}}^{\rm o}-\vec{\mathbf{k}}^{\rm i}$) will lead to the coherent accumulation of single-scattered waves on the basis of speckled illumination. In order to deal with uncontrolled phase shifts, we added additional phase correction factor, ϕ_j, for each illumination pattern. And the set of ϕ_j's that maximize the total intensity of the coherent summation will correspond to the uncontrolled phase shifts.

$$\max_{\phi_j}\sum_{\Delta\vec{\mathbf{k}}}\left|\sum_j\mathcal{E}_{\rm o}(\Delta\vec{\mathbf{k}};j)e^{i\phi_j}\right|^2. \quad (18)$$

For example, we present the obtained the uncontrolled phase shifts for the measurement in Supplementary Figure 11. Once this process is done, we multiply $e^{i\phi_j}$ to $\mathcal{E}_{\rm o}(\vec{\mathbf{k}}^{\rm o}-\vec{\mathbf{k}}^{\rm i};j)$ to which $\mathcal{U}_{\rm i}(\vec{\mathbf{k}}^{\rm i};j)^{-1}$ was multiplied to the time-gated reflection matrix $\mathcal{E}_{\rm o}(\vec{\mathbf{k}}^{\rm o};\vec{\mathbf{k}}^{\rm i})$ free of uncontrolled phase shifts. The aberration correction method discussed in the main manuscript was performed afterwards.

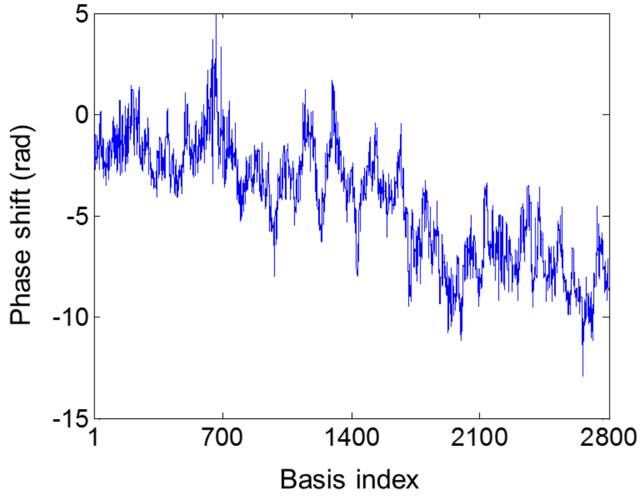

Supplementary Figure 11. The uncontrolled phase shifts for the experimental measurement in Fig. 2.

Detailed analysis of iteration process for the experimental data in Fig. 2

For the experimental data in Fig. 2, CLASS images are shown in Supplementary Figure 12a for different iteration number. The image quality was greatly enhanced only after the 1st round of the iteration. As the number of iterations was further increased, the pattern boundary was sharpened. In addition, the intensity of CLASS image was increased up to 25 times compared with the initial image. The plot in Supplementary Figure 12b shows the quantified increase of total intensity of CLASS image. Due to the proper accumulation of single-scattered waves by means of aberration correction, the signal to background intensity ratio was also enhanced (Supplementary Figure 12c) by about 25 times. The line profiles of images before and after the correction shown in Supplementary Figure 12d confirmed that line spacing of 600 nm, which corresponds to the diffraction limit of the system, was clearly resolved and the contrast of line structures was greatly enhanced.

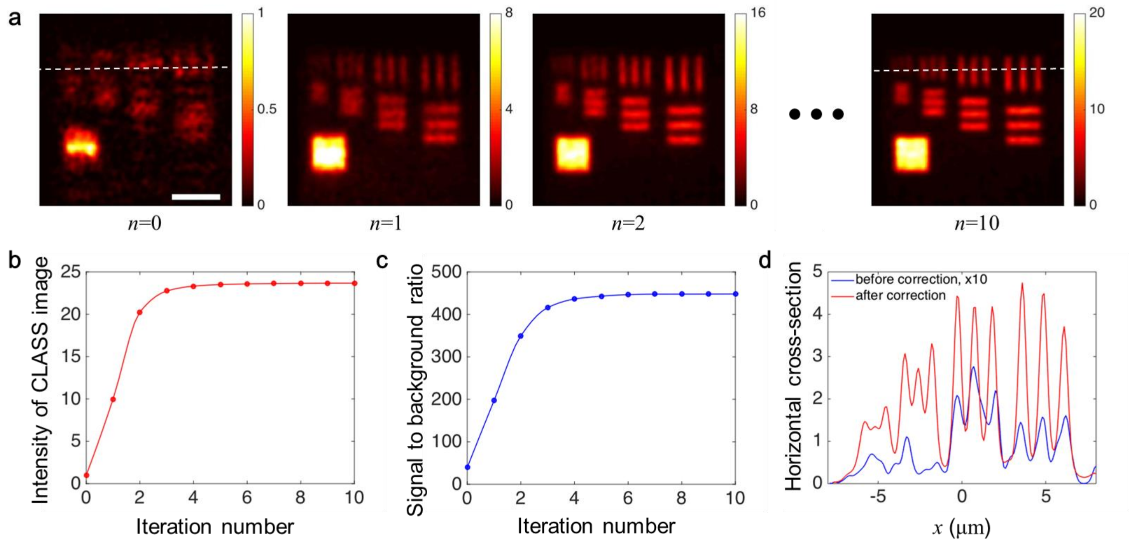

Supplementary Figure 12. Detailed analysis of experimental data in Fig.3. a, CLASS image after n^{th} iteration (n=0, 1, 2, and 10). n=0 corresponds to the initial CLASS image. Color scales indicate normalized intensity by the maximum intensity for n=0. Scale bar, 4 µm. **b**, Total CLASS intensity integrated over the entire view field as a function of the number of iterations. Data was normalized by the initial intensity (n=0). **c**, Signal to background intensity ratio. Signal intensity was the average intensity at the patterned area, and background intensity was the average intensity for the rest. **d**, Line profiles of images before and after aberration correction (dashed lines in **a** for n=0, and n=10). For better comparison, the line profile for n=0 case (blue line) is multiplied by a factor of 10.

Transfer function analysis of experimental data

For the experimental data in Fig. 3, we could perform similar transfer function analysis performed for the simulation data in section 2. If we assume that the initial aberration maps of the target were identical to the obtained aberration maps in Fig. 3i-j, we can estimate the initial amplitude transfer function of single-scattered waves in CASS image by Eq. (14). As shown in Supplementary Figure 13a, the initial ATF is not uniform, and has less than 10% of amplitude compared to the aberration-free case. Also, the effective bandwidth at k-space is reduced about half of ideal case, which indicates the reduction in resolving power. By the application of CLASS algorithm, the ATF converged to the aberration-free case (Supplementary Figure 13b). Indeed,

both the amplitude and effective bandwidth of ATF have increased by the iteration in accordance with the theoretical expectation.

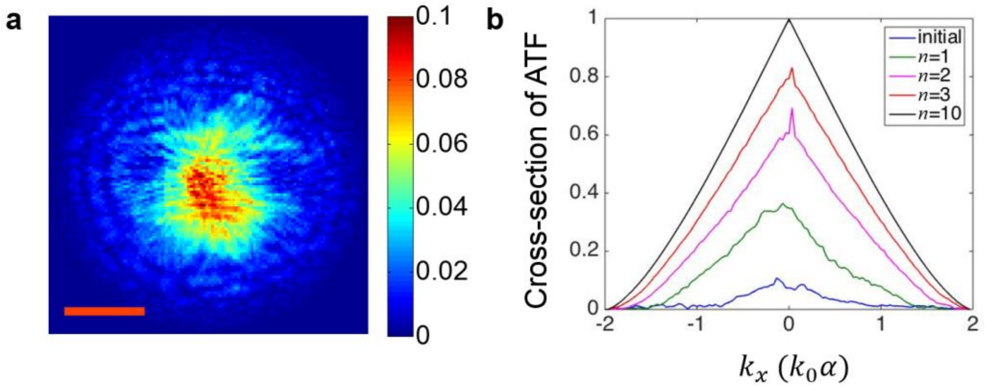

Supplementary Figure 13. Transfer function analysis for the experimental result in Fig. 3. a, Initial amplitude transfer function obtained by the cross-correlation of input and output aberration maps in Figs. 3i-j. Color scale is normalized by the maximum value of ideal transfer function. Scale bar, $k_0\alpha$. **b,** Line profiles of the amplitude transfer function at $k_y = 0$ for *n*=0, 1, 2, 3, and 10.

Comparison with Zernike-based adaptive optics

The major advancement that CLASS microscopy has made is the ability to characterize and eliminate steep variation of angle-dependent phase shifts induced by a thick scattering layer. The conventional adaptive optics experiments usually concern a few low-order Zernike modes, thereby confining their applicability to slowly varying angle-dependent phase shifts caused by mild aberrations of a thin scattering layer. To distinctively visualize the difference between CLASS microscopy and adaptive optics, we applied only the first 15 Zernike modes identified from the input and output aberration maps identified in Figs. 3i and 3j. As shown in Supplementary Figures 14a and 14b, fine details of aberration maps in Figs. 3i and 3j were missing for Zernike modes-based reconstruction. In addition, the coherent summation of single-scattered waves is not as effective as the result shown in Fig. 3l as indicated by the color scale. This suggests that even 15 orders of Zernike modes were not enough to deal with the aberration

of the scattering layer that we used. Indeed, CLASS microscopy can address steep variation of specimen-induced aberrations that conventional adaptive optics cannot handle.

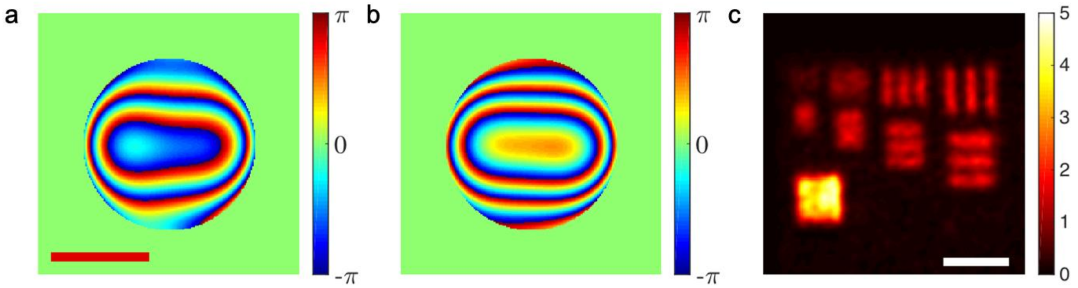

Supplementary Figure 14. Reconstructed image up to 15 Zernike polynomials. a-b, Aberration maps for input and output, respectively, up to the first 15 Zernike modes identified from the data in Figs. 3i and 3j. Color bar, phase angle in radian. Scale bar, $k_0\alpha$. **c**, Reconstructed CLASS image by the application of aberration correction maps in **a** and **b**. Scale bar, 4 μm. Color scale, intensity with the same unit as that in Fig. 3l.

The estimation of η in Fig. 3

There were about 20 times increase in signal intensity at the target in Fig. 3l in comparison with the initial intensity in Fig. 3d. But this doesn't mean that η is 1/20. In fact, the real η that we estimated by applying Eq. (16) to the measured aberration maps shown in Figs. 3i and 3j was $\eta = 1/204$. This discrepancy arises mainly because multiple-scattered waves as well as single-scattered waves contributed to the signal intensity at the target in Fig. 3d. Before the application of CLASS algorithm, the intensity of CLASS image at the target is the sum of single-scattered waves and the multiple-scattered waves that survived the time-gating and spatial coherence gating, i.e. $I_{\text{before}} = I_{\text{single}} + I_{\text{multi}}$ with initial SNR given by $I_{\text{single}}/I_{\text{multi}} = (\eta\gamma/\beta)N_{\text{m}}$. After the aberration correction, the intensity at the target becomes, $I_{\text{after}} = I_{\text{single}}/\eta + I_{\text{multi}}$ with SNR increased to $(\gamma/\beta)N_{\text{m}}$. Since the enhancement of the apparent signal intensity at the target was about 20 times, we can set up the following equation,

$$\frac{I_{\text{after}}}{I_{\text{before}}} = \frac{I_{\text{single}}/\eta + I_{\text{multi}}}{I_{\text{single}} + I_{\text{multi}}} = 20. \quad (19)$$

By inserting the estimated η from the measured aberration maps, we can obtain the initial single-to-multiple scattering ratio in the position basis,

$$\frac{I_{\text{single}}}{I_{\text{multi}}} = (\eta\gamma/\beta)N_{\text{m}} = 19/184 \simeq 0.1. \quad (20)$$

Therefore, multiple scattering was about 10 times stronger than the single scattering before the aberration correction, and this was responsible for the discrepancy mentioned above. Since the initial SNR of $(\eta\gamma/\beta)N_{\text{m}} \simeq 0.1$ was far less than unity, multiple scattering has strongly degraded the resolving power of imaging. By the aberration correction, this SNR was increased to $(\gamma/\beta)N_{\text{m}} \simeq 21.1$ by selectively enhancing the intensity of single-scattered waves.

The difference between input and output aberration maps

In the ideal case, the aberrations for the input and output should be identical in the reflection geometry since waves travel back and forth through physically the same sample. In the real practice, however, the optical system in the illumination beam path from the light source to the beam splitter and that in the collection beam path from the beam splitter to the camera are not perfectly the same. Therefore, input and output paths have different system aberrations. In addition, the slight misalignment of the optical axis or image focus can make the difference even larger. In general, the specimen-induced aberrations are much stronger than these system aberrations such that aberration maps for the input and output looked largely the same. For example, the normalized cross-correlation of input and output pupil functions given by the aberration maps in Figs. 3i and 3j, respectively, was 0.64. But when we looked at the phase difference between the two (Supplementary Figure 15), their difference was clearly visible. We could observe slowly varying phase difference across the pupil due to the difference of the system aberrations between the input and the output.

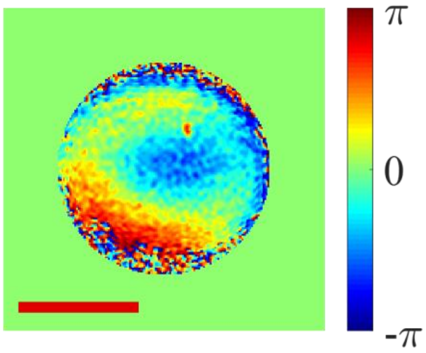

Supplementary Figure 15. Phase difference between input and output aberrations obtained in Fig. 3. Scale bar, $k_0\alpha$. Color bar, phase in radians.

Note that we also tried to correct aberration with the assumption that the input and output aberrations are identical. Supplementary Figure 16 below shows the identified aberration map and resulting CLASS image for the sample in Fig. 4. In comparison with Fig. 4c in the main text, the resolving power and signal intensity were significantly lower. This result shows both the merit and the importance of independently addressing the input and output aberrations.

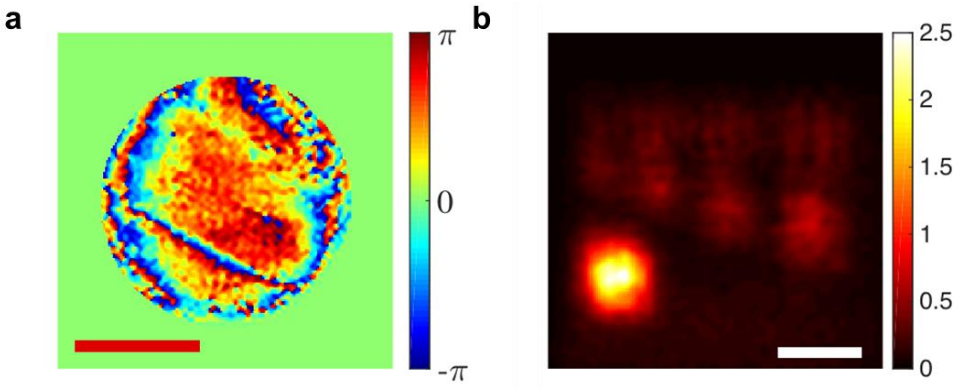

Supplementary Figure 16. Aberration correction of the data in Fig. 4 with the assumption that the input and output aberrations are identical. a, Acquired aberration map under the assumption of identical input-output aberration. Scale bar, $k_0\alpha$. Color bar, phase in radians. **b,** CLASS image after the aberration correction by **a**. Scale bar, 4 microns. Color bar, the intensity normalized by the maximum intensity in Fig. 4b

The measurements of the scattering mean free paths of scattering media

In order to determine the scattering mean free path of the scattering medium used in our study, we measured the intensity of ballistic photons as a function of the thickness of the scattering layer. We placed a stack of phantom scattering layers of known thickness on a flat mirror, and then measured the decay of the intensity of ballistic components depending on the number of phantom layers. Typical thickness of individual layer was 160 μm. Specifically, we illuminated a normally incident plane wave and recorded the complex field map of the backscattered waves. We then extracted the intensity of the normally reflecting plane wave component from the recorded map. By using the exponential curve fitting, we could obtain the decay constant of 51.2 μm for the roundtrip (Supplementary Figure 17). Therefore, the scattering mean free path of the scattering layer was 102.4 μm.

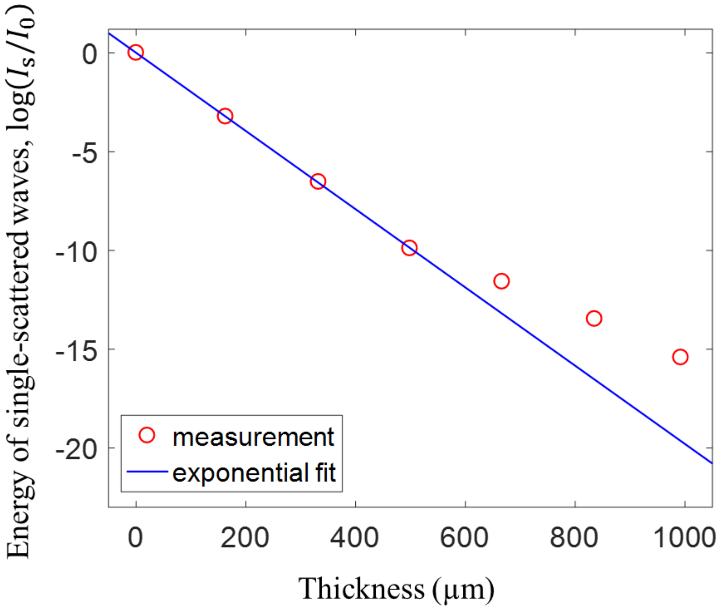

Supplementary Figure 17. Measurement of the scattering mean free path of the scattering layers used in Fig. 1.

Here one can notice that the first four points up to the thickness of 500 μm (1 mm in travel distance including the returning path, which corresponds to about 10 l_s) fit well to the exponential curve while the intensity taken at thicker depth was slightly larger than the fitted curve. In our measurement of the ballistic photons, we used both angular- and time-gating by the time-resolved complex field imaging. Like any other ballistic photon measurement methods, our method is effective up to a certain thickness of the sample. If the scattering medium becomes too thick, some of the multiple-scattered waves can pass through these gating operations, and this is source of discrepancy that we observed at the thicknesses larger than 500 μm. In fact, the conventional method mostly uses the angular gating in the transmission geometry (for instance, see Ref. 12 and the work by Emily J. McDowell et al.[2]), and its effectiveness was shown up to about 12 l_s (supplementary material in Ref. 12), similar range to our method. However, this limitation is not a problem at all in estimating the scattering mean free path of the phantom scattering layer. In preparing the scattering layer of an arbitrary thickness, we stacked multiple 160 μm-thick scattering layers made of the mixture of polystyrene beads and PDMS solution at a uniform concentration. Therefore, the reasonable measurements of ballistic photons up to the thickness of the scattering layer where the contribution of multiple scattering is negligible, which is about 500 μm in our case, will ensure the accurate estimation of the scattering mean free path of individual 160 μm-thick scattering layers. And this estimation will reasonably be applied to the multiple stacked layers.

In the case of rat brain tissues, scattering properties of the tissue slices vary with imaging position. Therefore, we estimated the scattering mean free path *in situ* from the total intensity of the CLASS image, which is close to the intensity of the ballistic photons, and the thickness of the tissue layer measured from the time delay. We took logarithm of this total intensity of the ballistic photons normalized by that measured with the mirror sample for the calculation of the scattering mean free path.

Supplementary Note 4. Additional experiments

In the main text, we used a resolution target having spatial variation of reflectance for demonstrating the CLASS microscopy. In this section, we provided experimental data for other types of targets such as a resolution target with phase contrast and point particles.

CLASS imaging of phase objects

In this section, we present experimental study of applying CLASS microscopy for phase-contrast targets. As a test target, we fabricated a gold layer coated on a slide glass in such a way that the thickness of the coating varies in space (Supplementary Figure 18a). In particular, the part of gold layer exhibiting the pattern of the resolution target was thicker than the rest by about 100 nm. Since the gold layer were coated over entire area, reflectance was almost uniform across the field of view. On the other hand, the reflected waves experience less phase retardation at the pattern than that at the rest. The height difference of about 100 nm corresponds to the phase retardation of 2 radians. Therefore, this target acts as a phase-contrast object.

Similar with Fig. 1b, we put both the aberrating layer and $5l_s$-thick scattering layer on the target as shown in Supplementary Figure 18a. Supplementary Figures 18b and 18c show the amplitude and phase maps, respectively, of CLASS image before aberration correction in which targets were invisible due to the aberration and multiple scattering. After applying for the CLASS algorithm, we could clearly identify target structures both in amplitude and phase maps (Supplementary Figures 18d and 18e). In the amplitude image (Supplementary Figure 18d), mainly the edges of the pattern were visible as the phase gradient there caused diffraction. On the other hand, the phase retardation occurred inside the pattern in the phase image (Supplementary Figure 18e). In Supplementary Figure 18f, we calculated the height of the pattern along the dashed line in Supplementary Figure 18e from the measured phase and confirmed that the calculated value was indeed very close to 100 nm. The aberration maps identified by the CLASS microscopy were

shown in Supplementary Figure 18g and 18h. Similar to the results in the main text, the asymmetric shape of aberration caused by the cylindrical dent was well visible.

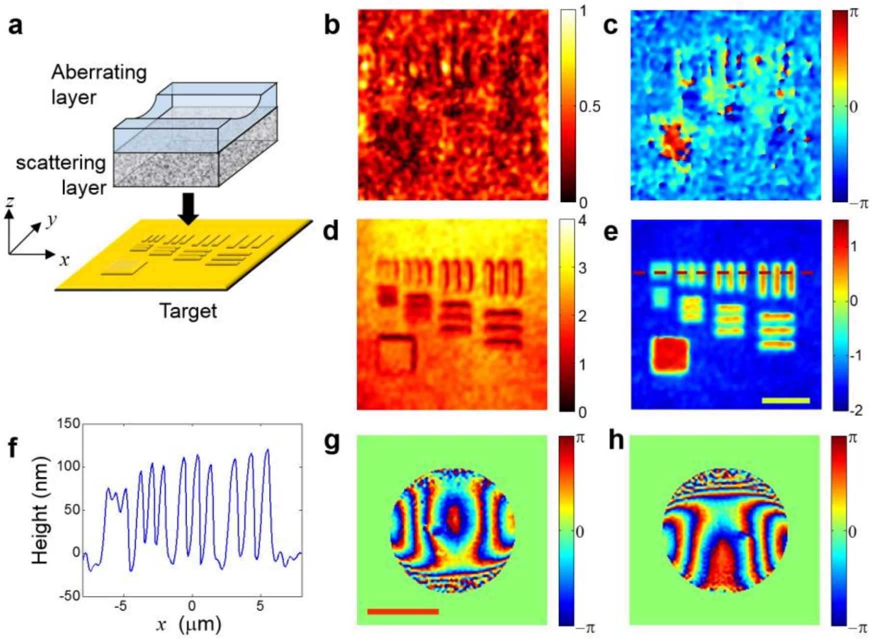

Supplementary Figure 18. Demonstration of CLASS microscopy with phase object. a, Layout of sample geometry. A patterned gold layer was placed underneath a set of aberrating layer and scattering layer. The thickness of the scattering layer corresponds to $5l_s$. **b-c**, Amplitude and phase maps of CLASS image, respectively, before the aberration correction. **d-e**, Amplitude and phase maps of CLASS image after aberration correction. Scale bar, 4 μm. Color scales in **b** and **d** were normalized by the maximum amplitude in **b**. Color scales in **c** and **e** are phase in radians. **f**, Height of the target along the dashed line in **e** estimated by the phase retardation. **g-h**, Aberration maps for input and output, respectively, identified by the CLASS microscopy. Scale bar, $k_0\alpha$. Color scales, phase in radian.

CLASS imaging of point particles

In this section, we demonstrated the CLASS microscopy for point-like objects. Gold nano-particles with the diameter of 400 nm were placed underneath the same set of aberrating and scattering layers used in section 4. In this particular experiment, the cylindrical groove in the aberrating layer was rotated by about 60 degrees with respect to horizontal axis. As shown in Supplementary Figure 19a, the image of particles were elongated along the orthogonal direction

to that of the groove because of the astigmatic aberration. After the aberration correction (Supplementary Figure 19b), individual particles were clearly visible. The input and output aberrations maps were shown in c and d, respectively. The average FWHM of individual particles in b was about 470±15 nm, which ensures the diffraction-limited spatial resolution of 600 nm.

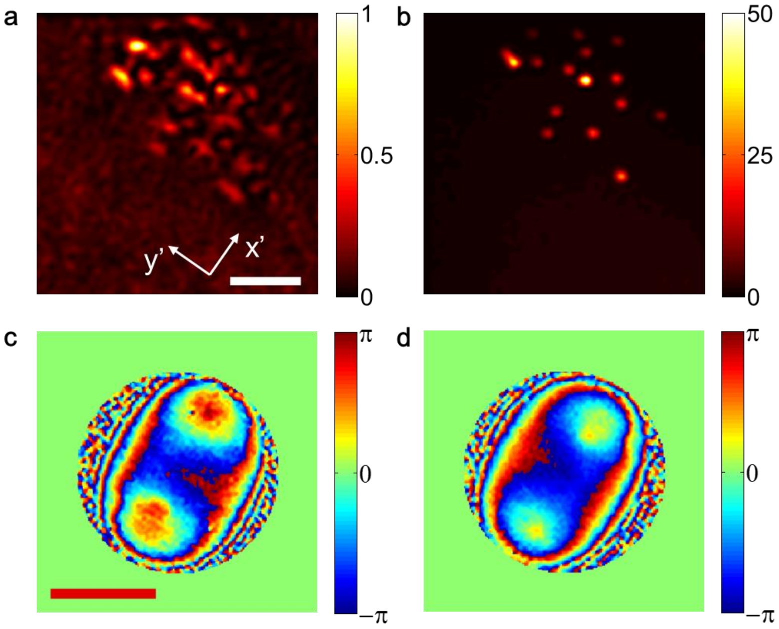

Supplementary Figure 19. Demonstration of CLASS microscopy for gold nanoparticles. a-b, CLASS images before and after the aberration correction. Scale bar, 4 μm, Color scale, normalized intensity. Cylindrical groove of aberrating layer was laid along *x'* direction. **c-d**, Measured aberration maps for the input and output. Scale bar, $k_0\alpha$. Color scales, phase in radian.

Supplementary References

1 Kang, S. *et al.* Imaging deep within a scattering medium using collective accumulation of single-scattered waves. *Nature Photonics* **9**, 253-258 (2015).

2 McDowell, E. J. *et al.* Turbidity suppression from the ballistic to the diffusive regime in biological tissues using optical phase conjugation. *Journal of biomedical optics* **15**, 025004-025004-025011 (2010).
