# Supplementary material for: High-resolution adaptive optical imaging within thick scattering media using closed-loop accumulation of single scattering
Source: Nat Commun. 2017 Dec 18;8:2157. doi: 10.1038/s41467-017-02117-8 (PMC5735168; doi:10.1038/s41467-017-02117-8)
Supplement: Supplementary file 2 — Peer Review File_NEW [file 41467_2017_2117_MOESM2_ESM.pdf]

Dear Editor

This study is devoted to optical imaging through thick scattering media. Conventional reflection imaging techniques, such as confocal microscopy or optical coherence tomography, suffer from the aberrations induced by the medium itself and from the multiple scattering events. Their approach is based on the experimental measurement of a time gated reflection matrix from the medium of interest. In a recent study published in Nature Photonics, the authors showed how to discriminate single scattering from the multiple scattering background from this reflection matrix [10]. In this paper, the authors extend this work by dealing with the aberration correction. To do so, they perform a closed-loop optimization of singly-scattered waves for both forward and phase-conjugation processes. They claim that they demonstrate "an enhancement of Strehl ratio by more than 1,000 times, two orders of magnitude improvement over conventional adaptive optics, and achieved the spatial resolution of 600 nm up to the unprecedented imaging depth of 7 scattering mean free paths. »

Although the topic is of interest for applications to medical imaging and that the paper contains interesting results, I have several strong objections for publication in Nature Communications that are listed below. As a general comment, I would say that the authors tend to oversell the results of their work. To my opinion, the originality of this work lies in applying conventional adaptive optics tools to the reflection matrix approach of optical imaging that the authors and others have introduced in Refs.10 and 12. Although the experimental results they show are interesting, I am very skeptical about the claim they make about the multiplication of the Strehl ratio by a factor >1000 (I will explain why below – see in particular my points 6 and 7). Their claim about the "unprecedented imaging depth of 7 scattering mean paths" with a diffraction limited resolution is in contradiction with the claim in Ref.10 that a diffraction-limited resolution was obtained through 11.5 scattering mean free paths.

I now explain my objections in more details

1/ Several times in the paper, the authors say that their method works in a "strong multiple scattering regime". To my opinion, this claim is exaggerated and in contradiction with the results they present. Their claim is based on the weak signal-to-noise ratio (or rather the single-to-multiple scattering ratio) they observe in the wavevector basis (pupil plane). However, such a ratio should be investigated in the real basis because this is in this basis that the image of the sample is built.

Typically, even at shallow depth (1 scattering mean free path), the single-to-multiple scattering ratio might be quite low in the wave vector basis but becomes very high in the imaging plane (Ref.12). Whereas single scattering signal spreads over all the elements of the reflection matrix in the wave vector basis, they only emerge along the close-diagonal elements of the reflection matrix in the real space, thus increasing the signal-to-noise ratio by a factor N_m, as stated in the manuscript. Fig.2d of the current ms shows the reflection matrix in the real basis. It is almost diagonal meaning that most of the recorded signal corresponds to single scattering. In a strong multiple scattering regime, this matrix would be, on the contrary, fully random (see for instance Ref.12). Hence, the authors cannot say that they are in a strong multiple scattering regime.

To be more quantitative: In the Supplementary material, the authors say that their approach is valid for a ratio $\beta/(\eta\gamma)$ <16000. Given the fact that $\eta = 1/400$ in their simulation, this means that this is valid for a multiple-to-single scattering ratio $\beta/\gamma < 40$ in the wave vector basis. In the imaging plane, the single-to-multiple scattering limit ratio scales as $N_m \gamma/\beta$, with $N_m = 2800$. Hence the single-to-multiple scattering ratio is of 70 in the imaging plane. We far from a strong multiple scattering regime where this ratio should be much smaller than 1…

2/ Related to my previous point, I think the title of this article is misleading. There is no suppression of scattering in this article. There is no multiple scattering contribution to remove as its contribution is already very weak in the original time gated confocal image. The CLASS microscopy enhances the single scattering contribution by correcting aberration but, by no means, removes multiple scattering.

3/ With regards to the imaging depth limit, the authors claim their approach allows to reach the "unprecedented imaging depth limit of 7 scattering mean paths". Besides the fact that this penetration depth may not be unprecedented (see above), the manuscript do not say how they measured the scattering mean free path in their samples. In the first experiment (Figs.1,2,3), they say that they place a $7l_s$ thick scattering layer underneath the aberrating layer. But they do not provide any detail on this scattering layer and do not say how the scattering mean free path was measured. In the second experiment (Fig.4 – rat brain tissue), they say that the scattering mean free path was approximately of 100 µm. How did they measure it?

4/ With regards to their CLASS algorithm, the authors say that they perform "a closed-loop optimization of signal waves for both forward and phase-conjugation processes. " I do not understand the interest of the phase conjugation step in their algorithm. I'm ok with the first step where they apply a correction on the reflection matrix at the input based on a conventional maximization of the single scattering intensity of the CLASS image. Then, the authors use a second step where they take the transpose conjugate of the reflection matrix (phase conjugation operation) and then apply a correction at the output still based on a maximization of the single scattering intensity. And finally iterate this process. I do not understand here the interest of the phase conjugation operation. Without the phase conjugation operation, it should be possible to correct for aberrations, first at the input, then at the output (by just transposing the reflection matrix), and iterate the process to determine the input and output phase laws that compensate for the aberrations. Could the authors comment on that aspect? Maybe, they could show how the algorithm would work without the phase conjugation operation.

5/ Given the experimental set up used in their work (FigS7), the input and output arms look identical in terms of optical paths. Hence, by virtue of spatial reciprocity, the aberration phase law should be the same at the input and the output. Could the authors comment on the fact that the aberration phase laws they experimentally determine with their algorithm look quite different at the input and output (see for instance Fig4 d and e)?

6/ I am very skeptical with regards to the claim made by the authors in the abstract that the Strehl ratio is increased by a factor >1000 with the CLASS algorithm.

In the first experiment described in the paper, the authors say that "the signal intensity was increased in magnitude by more than 20 times" (p13, line 295). A bit further, they claim that "The initial Strehl ratio S calculated from the acquired aberration maps was 1/1300" (p14, line 310). This is highly contradictory. After correction of aberrations, the single scattering intensity should be increased by a factor $1/S^2$ (the power 2 is due to the fact that aberrations are corrected both at the input and output, see for instance the Supplementary Material of Ref.12). Hence it means that the initial Strehl ratio should be $S = 1/\sqrt{20} = 0.22$. Hence, there is here a big contradiction with their own estimation of the Strehl ratio. How do they estimate it? Details about the estimation of the Strehl ratio are not provided.

There is the same contradiction for the second experiment. The signal intensity is increased by a factor 16 (Fig.4), which yields an estimation of the Strehl ratio of 0.25 whereas the authors claim a Strehl ratio of 1/740 (p16, line 363).

7/ Related to my last point: In the Supplementary material, the authors discuss the relation between the Strehl ratio S and η, which is the ratio of the total intensity of single-scattered waves in the presence of aberration with respect to that in the absence of aberration. For the authors, S and η should be the same for moderate aberrations. According to me, the scaling should be as $\eta = S^2$ (see my previous point - aberrations at input and output). But, even if we consider this scaling as correct, there is still a contradiction in their experiments. For the first experiment, the increase of the single scattering intensity is of $1/\eta$=20 whereas they estimate $1/S$=1300. According to Fig.S6(b), for $1/\eta$=20, 1/S and $1/\eta$ should be very close. The authors should absolutely clarify that point.

8/ Related to this relation between S and η, I don't understand why they should diverge at high aberration level. The explanation provided by the authors at page 11 of the supplementary material is not clear. Moreover, this in complete contradiction with Eqs.S15 and S16 that show an exactly similar expression for S and η.

9/ Several times in the paper, the authors claim that "Typical adaptive optics can deal with aberrations for S >0.1". I think it would be relevant to provide at least one reference to corroborate that statement.

10/ In the last experiment shown in the paper, the authors do not discuss about the increase of the signal intensity and do not give any estimation of the Strehl ratio. Yet, in Fig.5, it seems that the signal intensity is only increased by a factor 2, which is one order of magnitude smaller than the previous experiments. Is it because there is not too much aberration in their sample or is this because the CLASS approach does not work so well in speckle (the object to image in the two first experiments is a resolution target)? This limited performance should be discussed.

11/ In their conclusion, the authors say that one important aspect is that "aberration correction is performed over a wide field of view with finely stepped illumination angles". A larger field of view also implies the presence of several isoplanatic patches in the field of view. The aberration correction provided by the CLASS algorithm is limited to a field of view that contains one single isoplanatic patch. The authors should mention it in their conclusion. It is important to mention the improvements provided by the CLASS approach but also its limits.

Reviewer #2 (Remarks to the Author):

In the submitted manuscript the authors demonstrate a computational technique to mitigate the effect of sample induced aberrations in the context of their previously developed CASS technique. In their previous work (Kang et. al, Nat. Photon. 2015) the authors have demonstrated that the coherent summation of ballistic reflected light from a sample inside a scattering medium can lead to increased imaging quality in the presence of multiple scattered photons. However, the presence of surface aberrations together with the multiple light scattering would lead to the deterioration of the achieved image quality,

The novelty of the work relies in the addition of the computational step during the reconstruction process that allows for the cancellation of the phase retardations that the incoming and outgoing waves experience at the surface of the sample. Due to the coherent nature of the excitation and the detection the authors are able to post-hoc correct for these phase variations both under the excitation and detection light paths and increase the resulting image quality. They have demonstrated the applicability of their technique in artificial samples as well as ex vivo rabbit retina samples.

The manuscript is generally well written, the mathematical foundation of the technique is sufficiently documented and explained and the experimental results are convincing. Overall I consider the work an interesting enhancement of the previously developed technique with the potential to improve the imaging quality of coherent imaging methods.

- since the technique is a computationally enhancement of the previously developed method termed CASS, it will be limited by the same shortcomings. Mainly the applicability of the method would be rather limited to samples with a strong reflectivity. Could the authors add discussion of those shortcomings?
- In the part of the introduction where the authors communicate their method and place it with respect to the state of the art, I think that previous adaptive optics/ wavefront correction studies could be better presented. Among the cited techniques some have been able to correct for very high order modes (pupil plane aberrations and scattering that far exceed the ones the authors demonstrate in their figures) eg. references 17 and 19. Also, in the same context, other techniques (eg. Ji et. al, 2009, ref 15) could allow the correction of very steep phase gradients, similar to the ones demonstrated by the authors. Therefore the claim that the authors were able to correct aberrations that were previously outside the reach of adaptive optics is not accurate.
- Can the authors comment on how the input and output pupil aberration maps (eg. the ones in Fig. 3) are related to one another? Please show the phase difference.
- in page 4, the authors write "Therefore, it is possible and in fact god to correct the aberration of illumination, especially when nonlinear processes are involved." It can be argued that the correction of aberration only in the illumination path will suffice only when nonlinear process are involved. One-photon wide-field and confocal imaging methods require the correction of the aberration in the detection path to render diffraction-limited images.
- in the abstract, the use of the phrase "extremely swallow" could be avoided. I would suggest just "swallow" instead
- similarly, the phrase "ultra-high resolution imaging" in the title of the subsection is not quantitative. Consider revising.

Reviewer #1

This study is devoted to optical imaging through thick scattering media. Conventional reflection imaging techniques, such as confocal microscopy or optical coherence tomography, suffer from the aberrations induced by the medium itself and from the multiple scattering events. Their approach is based on the experimental measurement of a time gated reflection matrix from the medium of interest. In a recent study published in Nature Photonics, the authors showed how to discriminate single scattering from the multiple scattering background from this reflection matrix [10]. In this paper, the authors extend this work by dealing with the aberration correction. To do so, they perform a closed-loop optimization of singly-scattered waves for both forward and phase-conjugation processes. They claim that they demonstrate "an enhancement of Strehl ratio by more than 1,000 times, two orders of magnitude improvement over conventional adaptive optics, and achieved the spatial resolution of 600 nm up to the unprecedented imaging depth of 7 scattering mean free paths.

Although the topic is of interest for applications to medical imaging and that the paper contains interesting results, I have several strong objections for publication in Nature Communications that are listed below.

We appreciate the reviewer for carefully reading our paper and raising critical concerns. While we think that the reviewer's opinions are highly valuable, we found that some of the concerns were overly stringent and some due to the confusion and misunderstanding. In the following, we addressed all the concerns raised by the reviewer in a point-by-point basis.

As a general comment, I would say that the authors tend to oversell the results of their work. To my opinion, the originality of this work lies in applying conventional adaptive optics tools to the reflection matrix approach of optical imaging that the authors and others have introduced in Refs.10 and 12.

We strongly disagree with the reviewer's opinion that our work is the adaptation of conventional adaptive optics tools to the reflection matrix approach. In our study, we presented a novel adaptive optics method that enables us to independently identify the input and output aberrations from the elastically backscattered waves experiencing aberrations throughout their roundtrip to the target samples. This was done by the unique closed-loop optimization of the total intensity of the CLASS image for the forward and phase-conjugated processes, which has never been presented elsewhere. In the context of coherent imaging, the proposed method has significance in that it eliminates the needs for the guide stars and hardware-based iterative feedback control of the wavefront.

Throughout the remarks, the reviewer compared ref. 12 with our present work. The work in ref. 12 is certainly interesting, but we think that its scope is completely different from ours. It presents an effective method for enhancing the imaging depth, but at the expense of spatial resolution. The researchers in ref. 12 applied a loose-confocal type filter to the reflection matrix through which single-scattered waves and a fraction of multiple-scattered waves are filtered. The effective diameter of the filter was either 5 or 8 μm, which is about 10 times larger than the diffraction-limit spatial resolution. Then they identified eigenchannels maximizing the total reflection intensity of the filtered single- and multiple-scattered waves, and reconstructed object images from these eigenchannels. This approach can be effective in enhancing signal strength

due to the constructive interference of multiple scattering as well as single scattering, which led to the increased imaging depth. However, the same operation results in the reduction of the spatial resolution. Eigenchannels contain multiple-scattering information as well as single-scattering information, but multiple-scattering information was not used in such a way to keep track of the momentum change given by the target structure. Consequently, the spatial resolution presented in ref. 12 was a few microns, which is approximately the same as the effective diameter of the loose-confocal filter, even when the numerical aperture of the objective lens was 0.8.

For achieving the diffraction-limit spatial resolution, the image reconstruction should rely on the single-scattered waves, not the multiple-scattered waves, unless the trajectories of the multiple-scattered waves are deterministically accounted for. In our study, we selectively optimized the diagonal elements of reflection matrix in the position basis for the image reconstruction. And the CLASS algorithm is designed to exclusively increase the intensity of single-scattered waves at the diagonal, not the multiple scattering background (see Fig. S6) for counteracting the specimen-induced aberration of the single-scattered waves. As such, we could achieve spatial resolution of 600 nm for 0.8 NA at the unprecedented depth 7 l_s at such a high resolution. Indeed, our approach is designed to improve the imaging depth at which the diffraction-limit spatial resolution is maintained.

Although the experimental results they show are interesting, I am very skeptical about the claim they make about the multiplication of the Strehl ratio by a factor >1000 (I will explain why below – see in particular my points 6 and 7). Their claim about the "unprecedented imaging depth of 7 scattering mean paths" with a diffraction limited resolution is in contradiction with the claim in Ref.10 that a diffraction-limited resolution was obtained through 11.5 scattering mean free paths.

The reviewer's concern about our claim of Strehl ratio enhancement is mainly due to the confusion about the definitions of Strehl ratio S and the single-scattering enhancement factor η. We clarified them when we address the reviewer's points #6 and #7.

Regarding the concern that our claim of imaging depth is contradictory to our previous paper, we would like to stress that it is meaningless to talk about imaging depth without considering the spatial resolution. For example, the medical ultrasound imaging can see deeper than any other optical imaging methodologies, but its spatial resolution is far worse than optical imaging. In our previous study, we demonstrated imaging depth of 11.5 l_s for the numerical aperture (NA) of 0.4. The corresponding diffraction-limit spatial resolution was about 1.2 μm. On the other hand, we achieved spatial resolution of 600 nm in the present study, which is the diffraction-limit resolution set by 0.8 NA objective lens, to the imaging depth of 7 l_s. Although 7 l_s is shallower than 11.5 l_s, this is an unprecedented imaging depth where the coherent imaging works at such a high resolution. Considering both imaging depth and spatial resolution altogether, our claim is not contradictory at all to the previous publication.

It is not a straightforward task to maintain the diffraction-limit spatial resolution up to high numerical aperture. High spatial frequency components are much more prone to the sample-induced aberrations than low spatial frequency components, and the correction of aberration becomes essential for achieving the diffraction-limit resolution. For a heuristic purpose, we present in Fig. R1 a plot of S and η for different NA values for the case of spherical aberration induced by a 1 mm-thick PDMS layer. The effect of aberration rapidly increases as the NA exceeds 0.5. The Strehl ratio and η were about 0.9 for 0.4 NA, which indicates that the effect of

aberration was negligible at the condition of Ref. 10. But for 0.8 NA, these values were reduced to about 1/50 and 1/20, respectively. Moreover, we introduced astigmatic aberrations (Fig. 1) in addition to the spherical aberration by 7 l_s thick PDMS layer, which further attenuated S to about 1/1300 and η to about 1/200. Consequently, the effect of aberration in our current experiment was much more severe than Ref. 10, and the correction of aberrations has been critical.

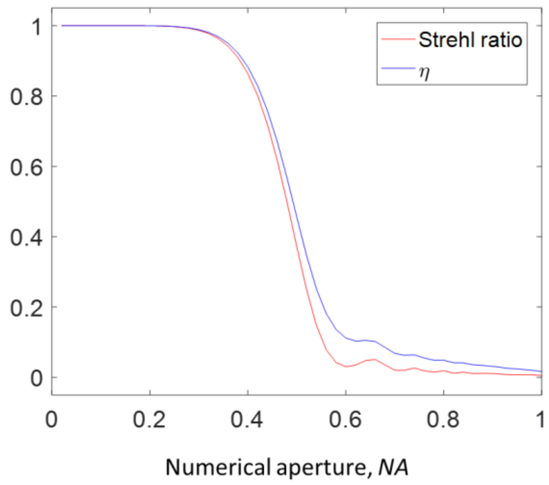

Figure R1. Strehl ratio S and the single-scattering enhancement factor η depending on the numerical aperture for spherical aberration induced by 1mm-thick PDMS layer

1. Several times in the paper, the authors say that their method works in a "strong multiple scattering regime". To my opinion, this claim is exaggerated and in contradiction with the results they present. Their claim is based on the weak signal-to-noise ratio (or rather the single-to-multiple scattering ratio) they observe in the wavevector basis (pupil plane). However, such a ratio should be investigated in the real basis because this is in this basis that the image of the sample is built.

We conducted image acquisition by choosing the diagonal elements of reflection matrix in the real basis. And the initial single-to-multiple scattering ratio was well below unity even in the real basis because sample-induced aberrations significantly attenuated the single scattering intensity. Therefore, it is legitimate to claim that our method works at the strong multiple scattering regime. We quantitatively addressed this reviewer's concern about the expression, 'strong multiple scattering regime,' in response to reviewer's points #2, #7, and #10.

Here, we clarify the reason why we use the wavevector basis as a starting point rather than the real basis. If we consider only the multiple scattering, the choice of basis is not important. As presented in Figs. 2c-d, both bases can be transformed into one another by means of Fourier transform or inverse Fourier transform. In the presence of aberration, however, the wavevector basis has a direct relevance. Aberration is the angle-dependent phase retardation of the single-scattered waves. This means that we should correct aberration in the angular basis (or equivalently the wavevector basis) to counteract the phase retardation at each wavevector. This

is the reason why most of the adaptive optics techniques put wavefront correction device at the pupil plane. Likewise, we applied aberration correction in the wavevector basis in such a way to maximize the total intensity in the diagonal components of the real basis.

Typically, even at shallow depth (1 scattering mean free path), the single-to-multiple scattering ratio might be quite low in the wavevector basis but becomes very high in the imaging plane (Ref.12). Whereas single scattering signal spreads over all the elements of the reflection matrix in the wave vector basis, they only emerge along the close-diagonal elements of the reflection matrix in the real space, thus increasing the signal-to-noise ratio by a factor Nm, as stated in the manuscript. Fig.2d of the current ms shows the reflection matrix in the real basis. It is almost diagonal meaning that most of the recorded signal corresponds to single scattering. In a strong multiple scattering regime, this matrix would be, on the contrary, fully random (see for instance Ref.12). Hence, the authors cannot say that they are in a strong multiple scattering regime.

Even after the application of time-gated detection, the initial single-to-multiple scattering ratio at the diagonal of real basis in Fig. 2d was well below unity. Therefore, it is fair to state that the imaging condition was in strong multiple scattering regime. We are not sure why the strong multiple scattering regime should be defined by the condition that multiple scattering fully cover all the off-diagonal components of reflection matrix in the real basis. We think that what matters most is whether the initial SNR of imaging is well below unity or not.

In Figs. 2c and 2d, we deliberately reduced the spatial frequency bandwidth to $|k| < 0.26k_0$ to better visualize the matrix elements in the wavevector basis. But this led to the dominant display of the close-diagonal elements in Fig. 2d as the reviewer observed. From the reviewer's comment, we realized that it is more important to show the spread of diagonal elements in the real basis. Therefore, we present original matrices with the full spatial frequency bandwidth of $|k| < 0.8k_0$ in Fig. R2 below. As shown in the figure, the single-scattered waves were significantly spread to off-diagonal elements due to the strong aberrations, and strong speckled multiple-scattered waves were superimposed on the top of these single-scattered waves. And the rest of the multiple-scattered waves were distributed across the whole plane.

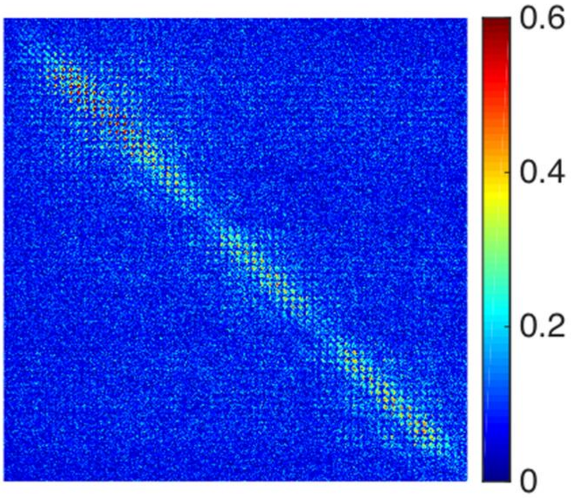

Figure R2. Full data set of time resolved reflection matrix $E(\vec{r}^{o};\vec{r}^{i},\tau_0)$ in Fig. 2. Color bar, amplitude in arbitrary unit.

In response to the reviewer's comment, we replaced Fig. 2c with the reflection matrix in the wavevector basis covering full 0.8 NA and Fig. 2d with Fig. R2. And we added the following sentence to the main text for the description of this new Fig. 2d.

"In Fig. 2d, we can observe that the single-scattered waves were significantly spread to off-diagonal elements due to the strong aberrations, and strong speckled multiple-scattered waves are superimposed on the top of these single-scattered waves."

To be more quantitative: In the Supplementary material, the authors say that their approach is valid for a ratio $\beta/(\eta\gamma)$ <16000. Given the fact that η = 1/400 in their simulation, this means that this is valid for a multiple-to-single scattering ratio β/γ < 40 in the wave vector basis. In the imaging plane, the single-to-multiple scattering limit ratio scales as Nm γ/β, with Nm = 2800. Hence the single-to-multiple scattering ratio is of 70 in the imaging plane. This is far from a strong multiple scattering regime where this ratio should be much smaller than 1.

If there is no aberration, the single-to-multiple scattering ratio should be N_m (γ/β) =70 in the real basis, and target samples must have been readily resolved even without the use of CLASS algorithm. However, this estimation is not valid anymore when there exist aberrations. The single-to-multiple scattering ratio is reduced by η due to the aberration, and the initial ratio is reduced to $(\gamma/\beta)\eta$ =70/400 =0.175<1 even in the real basis. In other words, multiple scattering dominates single scattering in the real basis because the single scattering signal is attenuated by the aberration. This is the reason why the target samples were invisible before the application of CLASS algorithm. Considering this initial condition, it is legitimate to state that CLASS algorithm has dealt with both strong multiple scattering and aberration.

For the experimental data shown in Fig. 3, the initial single-to-multiple scattering intensity ratio was estimated to be $(\eta\gamma/\beta)N_m \simeq 0.1$. Therefore, we added the following sentence to the revised manuscript.

“Further analysis revealed that the initial single-to-multiple scattering intensity ratio was $\eta(\gamma/\beta)N_m \simeq 0.1$ (see detailed analysis for Supplementary section III. 6).”

2. Related to my previous point, I think the title of this article is misleading. There is no suppression of scattering in this article. There is no multiple scattering contribution to remove as its contribution is already very weak in the original time gated confocal image. The CLASS microscopy enhances the single scattering contribution by correcting aberration but, by no means, removes multiple scattering.

Once again, the reviewer’s judgement excluded the effect of aberration. As we responded to the reviewer’s point #1, our experimental condition is such that the combined effect of multiple scattering and aberration attenuated the initial SNR well below unity even in the real basis, i.e. $\eta(\gamma/\beta)N_m$ <1. Our CLASS algorithm resolves both the degradation of SNR due to multiple scattering and reduction of spatial resolution due to aberration by raising η. Therefore, we think that the title of the article is relevant.

3. With regards to the imaging depth limit, the authors claim their approach allows to reach the “unprecedented imaging depth limit of 7 scattering mean paths”. Besides the fact that this penetration depth may not be unprecedented (see above), the manuscript do not say how they measured the scattering mean free path in their samples. In the first experiment (Figs.1,2,3), they say that they place a 7ls thick scattering layer underneath the aberrating layer. But they do not provide any detail on this scattering layer and do not say how the scattering mean free path was measured. In the second experiment (Fig.4 – rat brain tissue), they say that the scattering mean free path was approximately of 100 µm. How did they measure it?

We appreciate the reviewer for pointing this out. We placed a stack of phantom layers of known thickness on a flat mirror, and then measured the decay of the intensity of ballistic components depending on the number of phantom layers. Specifically, we illuminated a normally incident plane wave and recorded the complex field map of the backscattered waves. We then extracted the intensity of the normally reflecting plane wave component from the recorded map. By using the exponential curve fitting, we could obtain the decay constant of 51.2µm for the roundtrip (Fig. R3). Therefore, the scattering mean free path of the scattering layer was 102.4 µm.

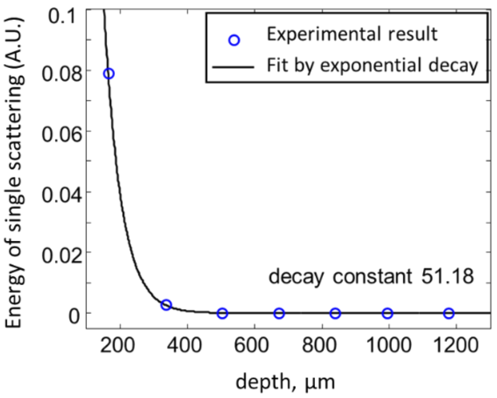

Figure R3. Calibration of scattering mean free path

In the case of rat brain tissues, scattering properties of the tissue slices vary with imaging position. Therefore, we estimated the scattering mean free path *in situ* from the total intensity of the CLASS image, which is close to the intensity of the ballistic photons, and the thickness of the tissue layer measured from the time delay. We added these details about measuring the scattering mean free path to the section III.8 of SI.

4. With regards to their CLASS algorithm, the authors say that they perform "a closed-loop optimization of signal waves for both forward and phase-conjugation processes. " I do not understand the interest of the phase conjugation step in their algorithm. I'm ok with the first step where they apply a correction on the reflection matrix at the input based on a conventional maximization of the single scattering intensity of the CLASS image. Then, the authors use a second step where they take the transpose conjugate of the reflection matrix (phase conjugation operation) and then apply a correction at the output still based on a maximization of the single scattering intensity. And finally iterate this process. I do not understand here the interest of the phase conjugation operation. Without the phase conjugation operation, it should be possible to correct for aberrations, first at the input, then at the output (by just transposing the reflection matrix), and iterate the process to determine the input and output phase laws that compensate for the aberrations. Could the authors comment on that aspect? Maybe, they could show how the algorithm would work without the phase conjugation operation.

As the reviewer conjectured, the complex conjugation operation is not critical from the mathematical point of view in the second step of CLASS algorithm. It doesn't make any difference in the result except for the sign of the output phase correction map. What is important is the transpose operation, which enables us to swap input and output, and to correct the specimen-induced aberration from the output side. The reason we take phase conjugation (transpose + complex conjugation) rather than just the transpose is to impose a physical meaning to the operation. In this way, the second step is equivalent to physically sending the waves from the output side and measuring the backscattered waves at the input side.

5. Given the experimental set up used in their work (FigS7), the input and output arms look identical in terms of optical paths. Hence, by virtue of spatial reciprocity, the aberration phase law should be the same at the input and the output. Could the authors comment on the fact that the aberration phase laws they experimentally determine with their algorithm look quite different at the input and output (see for instance Fig4 d and e)?

As the reviewer mentioned, the aberrations for the input and output should be identical in reflection geometry due to the spatial reciprocity. In the real practice, however, the optical system in the illumination beam path from the light source to the beam splitter and that in the collection beam path from the beam splitter to the camera are not perfectly the same. In addition, there may be slight misalignment of optical axis and image focus between the two beam paths. Therefore, the input and output can have different system aberrations. But these system aberrations are relatively smaller than the specimen-induced aberrations, and the aberration maps for the input and output are largely similar. For example, the normalized cross-correlation of the complex pupil functions of the aberration maps shown in Figs. 3i and 3j was about 0.64. In the case of the correlation between the maps in Fig. 4d and 4e, their correlation was still 0.46, but a bit lower than the phantom case since the magnitude of sample-induced aberrations was

smaller. Note that the coordinate systems are flipped between the input and the output, and this needs to be taken into account when they are compared.

We tried to correct aberration with the assumption that the input and output aberrations are identical. Figure R4 below shows the identified aberration map and resulting CLASS image for the sample in Fig. 4. In comparison with Fig. 4c in the main text, the resolving power and signal intensity were significantly reduced. This result shows the importance of independently addressing the input and output aberrations.

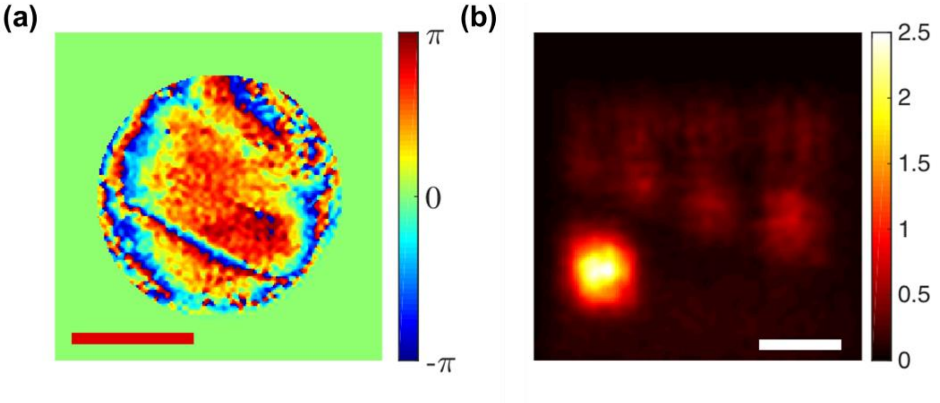

Figure R4. Aberration correction of the data in Fig. 4 with the assumption that the input and output aberrations are identical. **a,** Acquired aberration map under the assumption of identical input-output aberration. Scale bar, $k_0\alpha$. **b,** CLASS image after the aberration correction by **a**. Scale bar, 4 microns. Color bar, the intensity normalized by the maximum intensity in Fig. 4b.

We added the following sentences to the revised manuscript and these new analyses to the Supplementary section III.7.

"The input and output aberration maps are largely the same as waves travel back and forth through physically the same sample in the reflection geometry. But due to the difference in the system aberrations between the illumination beam path from the light source to the beam splitter and the collection beam path from the beam splitter to the camera, they have a slight difference (see Supplementary section III.7)."

6. I am very skeptical with regards to the claim made by the authors in the abstract that the Strehl ratio is increased by a factor >1000 with the CLASS algorithm.

In the first experiment described in the paper, the authors say that "the signal intensity was increased in magnitude by more than 20 times" (p13, line 295). A bit further, they claim that "The initial Strehl ratio S calculated from the acquired aberration maps was 1/1300" (p14, line 310). This is highly contradictory. After correction of aberrations, the single scattering intensity should be increased by a factor $1/S^2$ (the power 2 is due to the fact that aberrations are corrected both at the input and output, see for instance the Supplementary Material of Ref.12). Hence it means that the initial Strehl ratio should be $S = 1/\sqrt{20} = 0.22$. Hence, there is here a big contradiction with their own estimation of the Strehl ratio. How do they estimate it? Details about the estimation of the Strehl ratio are not provided.

There is the same contradiction for the second experiment. The signal intensity is increased by a factor 16 (Fig.4), which yields an estimation of the Strehl ratio of 0.25 whereas the authors claim a Strehl ratio of 1/740 (p16, line 363).

At first, we clarify the definitions of S and η, and then discuss their physical meaning. In fact, the definitions of S and η, and the way they were estimated were described in full detail in the section I.6 of the previous SI (II.5 in the revised SI). In adaptive optics, S is defined by the peak intensity of the point-spread-function (PSF) in the presence of aberration relative to that without aberration. For the case when both the input and output aberrations matter, this parameter is written as

$$S = \left|\langle \mathcal{A}(\vec{k}=0)\rangle\right|^2 / \left|\langle \mathcal{A}_0(\vec{k}=0)\rangle\right|^2. \qquad \text{(R1)}$$

Here $\mathcal{A} = P_i^{a*} \star P_o^a$ is the amplitude transfer function combining both input and output aberrations and $\mathcal{A}_0(\vec{k})$ is the ideal amplitude transfer function.

On the other hand, the factor η in Eq. (3) is defined by the ratio of the 'total' intensity, not the peak intensity, of the PSF in the presence of aberration with respect to that in the absence of aberration. In terms of the amplitude transfer function, η is written as

$$\eta = \langle\left|\mathcal{A}(\vec{k})\right|^2\rangle / \langle\left|\mathcal{A}_0(\vec{k})\right|^2\rangle. \qquad \text{(R2)}$$

Due to these differences in the definition, the relation $S^2 = \eta$ or $S = \eta$ that the reviewer conjectured doesn't exist. Certainly, S and η are related to a certain extent. The increase of aberration accompanies the simultaneous reduction in the peak intensity and the total intensity of the PSF. However, the peak height is more susceptible than the total intensity to the aberration. Therefore, S is usually smaller than η. But their relation depends on the type of aberration, which makes it difficult to find a general relation. For this reason, we only plotted S and η for one representative example of aberration in Fig. S6 (Fig. S7 in the revised SI).

Let us discuss the physical meaning of S and η. S is a parameter used to describe the aberration-induced signal reduction in the confocal and two-photon microscopes. It is a directly relevant parameter to the general adaptive optics audience because it indicates the degree of aberrations of the samples used in our study.

On the other hand, η describes the reduction of single-scattering intensity in the CLASS microscopy where images are acquired over wide area. Unlike confocal microscopy where signal is collected only at the confocal pinhole, the entire PSF contributes to the signal intensity in the wide-field detection. Therefore, the degradation of SNR in the CLASS microscope is determined by η, not by S. Likewise, the main role of CLASS algorithm is to increase single scattering intensity by $1/\eta$, not by $1/S$.

Now let's go back to the reviewer's comment. When we described the enhancement of signal intensity by either 20 times or 16 times, it is related to the increase of η, not S (the exact relation between 20 times and η is given below in response to reviewer's point #7). Therefore, the reviewer's estimation to deduce S from the signal enhancement is not relevant. Once again, both S and η are determined by the aberrations, but they are not equal.

7. Related to my last point: In the Supplementary material, the authors discuss the relation between the Strehl ratio S and η, which is the ratio of the total intensity of single-scattered waves in the presence of aberration with respect to that in the absence of aberration. For the authors, S and η should be the same for moderate aberrations. According to me, the scaling should be as $\eta = S^2$ (see my previous point - aberrations at input and output). But, even if we consider this scaling as correct, there is still a contradiction in their experiments. For the first experiment, the increase of the single scattering intensity is of 1/ η=20 whereas they estimate 1/ S=1300. According to Fig.S6(b), for 1/ η=20, 1/S and 1/ η should be very close. The authors should absolutely clarify that point.

As we clarified in the comment #6, the relation, $S^2 = \eta$ or $S = \eta$ that the reviewer conjectured doesn't exist.

Now let us clarify η in Fig. 3. Indeed, we wrote in the main text that "Moreover, the signal intensity was increased in magnitude by more than 20 times, suggesting that the cross-correlation of the aberration-corrected pupil functions had been increased". Here the 20 times increase in signal intensity was estimated by comparing the intensity at the target in Fig. 3d and that in Fig. 3l. But this doesn't mean that η was 1/20. In fact, the real η that we estimated by applying Eq. (R2) to the measured aberration maps shown in Figs. 3i and 3j was $\eta = 1/204$ $(S = 1/1300)$.

There is a discrepancy between the apparent signal enhancement of about 20 times estimated from Figs. 3d and 3l and the increase of single scattering intensity by $1/\eta$~200. This was mainly because multiple-scattered waves as well as single-scattered waves contributed to the signal intensity at the target in Fig. 3d. Before the application of CLASS algorithm, the intensity of CLASS image at the target is the sum of single-scattered waves and the multiple-scattered waves that survived the time-gating as well as spatial coherence gating, i.e. $I_{before} = I_{single} + I_{multi}$ with initial SNR given by $I_{single}/I_{multi} = (\eta\gamma/\beta)N_m$. After the aberration correction, the intensity at the target becomes, $I_{after} = I_{single}/\eta + I_{multi}$ with SNR increased to $(\gamma/\beta)N_m$.

Since the enhancement of the apparent signal intensity at the target was about 20 times, we can set up the following equation,

$$\frac{I_{after}}{I_{before}} = \frac{I_{single}/\eta + I_{multi}}{I_{single} + I_{multi}} = 20. \quad \text{(R3)}$$

By inserting the estimated η from the measured aberration maps, we can obtain the initial single-to-multiple scattering ratio in the position basis,

$$\frac{I_{single}}{I_{multi}} = (\eta\gamma/\beta)N_m = \frac{19}{184} \simeq 0.1.$$

Therefore, multiple scattering was about 10 times stronger than the single scattering before the aberration correction, and this was responsible for the discrepancy mentioned above. Since the initial SNR of $(\eta\gamma/\beta)N_m \simeq 0.1$ was far less than unity, multiple scattering has strongly degraded the resolving power of imaging. By the aberration correction, this SNR was increased to $(\gamma/\beta)N_m \simeq 21.1$ by selectively enhancing the intensity of single-scattered waves.

Regarding the discussion on Fig. S6(b) (Fig. S7(b) in the revised SI), S and η were acquired for the numerically assumed aberrations shown in Figs. S1a and S1b in the revised SI. For these

aberrations, $S = 1/3600$ at $\eta = 1/400$. In the case of experimental result shown in Fig. 3, $S = 1/1300$, and $\eta = 1/204$. Although the exact relation between S and η varies depending on the type of aberrations, the relative values of S to η in simulations and experiments are reasonably similar.

We added the following sentences to the revised manuscript to respond to all the issues raised by the reviewer. And the detailed discussion given above is added to the section III.6 of the revised SI.

"Moreover, the signal intensity at the target estimated from Fig. 3d and 3l was increased in magnitude by more than 20 times, suggesting that the cross-correlation of the aberration-corrected pupil functions had been increased."

"From the acquired angle-dependent phase correction maps in Figs. 3i and 3j, the initial Strehl ratio (see Supplementary section II.5 for the definition) was estimated to be S = 1/1300. This is two orders of magnitude smaller than the conventional adaptive optics typically handles. We also obtained $\eta = 1/204$ from Eq. (3) and measured aberration maps, which means that the total single scattering intensity was increased by about 200 times after the application of CLASS algorithm. There was a discrepancy between the apparent signal enhancement of about 20 times estimated from Figs. 3d and 3l and the increase of single scattering intensity by 200 times. And this was mainly because multiple-scattered waves as well as single-scattered waves contributed to the signal intensity at the target in Fig. 3d. Further analysis revealed that the initial single-to-multiple scattering intensity ratio was $\eta(\gamma/\beta)N_m \simeq 0.1$ (see detailed analysis for Supplementary section III. 6)."

8. Related to this relation between S and η, I don't understand why they should diverge at high aberration level. The explanation provided by the authors at page 11 of the supplementary material is not clear. Moreover, this in complete contradiction with Eqs.S15 and S16 that show an exactly similar expression for S and η.

As we clarified in the response to the reviewer's point #6, $S \neq \eta$ from the definitions shown in Eqs. S15 and S16. The discrepancy between S and η increases with the increase of the degree of aberration as the peak height of the PSF is more susceptible than its total intensity to the aberrations.

9. Several times in the paper, the authors claim that "Typical adaptive optics can deal with aberrations for S >0.1". I think it would be relevant to provide at least one reference to corroborate that statement.

We appreciate the reviewer's suggestion, and added the following reference to the revised manuscript.

X. Tao et al., Adaptive optics confocal microscopy using direct wavefront sensing, Optics Letters **36,** 1062 (2011).

10. In the last experiment shown in the paper, the authors do not discuss about the increase of the signal intensity and do not give any estimation of the Strehl ratio. Yet, in Fig.5, it seems that the signal intensity is only increased by a factor 2, which is one order of magnitude smaller than the previous experiments. Is it because there is not too much aberration in their sample or is this because the CLASS approach does not work so well in speckle (the object to image in the two first experiments is a resolution target)? This limited performance should be discussed.

The apparent signal increase at the fungal filament was about 9.05 times, not 2 times in Fig. 5h. In fact, the images were a bit saturated for better visualization of the fungal structures across the entire view field. Signal enhancement was smaller than the phantom case, but not by an order of magnitude.

As discussed above, this apparent signal increase is not equal to the enhancement of single scattering intensity η. From the obtained aberration maps of the output (Fig. 5j) and input (not shown in the figure), we obtained S=1/437 and η=1/44.7. The initial single-to-multiple scattering ratio was $(\eta\gamma/\beta)N_m \sim 0.23$ from the same analysis given to the reviewer's point #7.

For the case of the specimen in Fig. 5i, the enhancement of intensity was about 3.49 with S=1/890, η=1/68.2, respectively. With these parameters, the initial single-to-multiple scattering ratio was estimated to be about 0.04, which indicates that the biological specimens were under the influence of strong multiple scattering.

In imaging biological tissues, the single scattering intensity is inevitably smaller than the resolution target case. Therefore, the degree of multiple scattering and aberrations that can be overcome tends to be smaller. But what is important in assessing the performance of the imaging method is the initial single-to-multiple scattering ratio that the method can deal with. In this way, the performance can be characterized independent of the types of samples. In this respect, our CLASS microscope works equally well for biological tissues since we could handle initial SNR as small as 0.04 even for biological specimens.

In response to the reviewer's comment, we added S, η and initial SNR of the biological data to the revised manuscript. Also, the following discussion on the limited performance of CLASS microscope (in fact, all the coherent imaging method) for biological specimens was added to the conclusion section.

"On the other hand, CLASS microscopy requires target samples to have sufficient reflectivity for its best performance in imaging depth similar to the other coherent imaging methods working in the epi-detection geometry. In fact, the maximum possible imaging depth is determined by the reflectivity of the sample as well as the scattering properties of the scattering medium. If the reflectivity of the sample is reduced by 1/10, then the imaging depth should be reduced by 1 scattering mean free path."

11. In their conclusion, the authors say that one important aspect is that "aberration correction is performed over a wide field of view with finely stepped illumination angles". A larger field of view also implies the presence of several isoplanatic patches in the field of view. The aberration correction provided by the CLASS algorithm is limited to a field of view that contains one single isoplanatic patch. The authors should mention it in their conclusion. It is important to mention the improvements provided by the CLASS approach but also its limits.

In our experiment, we could obtain the spatial resolution of 600 nm across the entire view field of $30 \times 30\ \mu m^2$ even at the severe aberrations shown in Fig. 3. This is mainly because we corrected aberrations for 2,800 wavevectors covering all the orthogonal modes given by the view field and numerical aperture. In fact, this is the maximum degree of correction that can be made for the given view field. As a consequence, the entire view field was seen as a single isoplanatic patch. Therefore, the CLASS algorithm is not limited to the field of view that contains one single isoplanatic patch, but enables us to treat the view field as a single isoplanatic patch by employing the maximum available degree of aberration corrections.

If the number of wavevectors is reduced by increasing the angular stepping, then the reviewer's reasoning is correct. In such case, the view field should be split into multiple isoplanatic patches, and corrections should be made to individual patches. In fact, this is the case for conventional adaptive optics. The number of modes that deformable mirrors can control is much smaller than the number of orthogonal modes set by the view field and the numerical aperture in ordinary adaptive optics. Because of this deficiency in the number of control, the area where the diffraction-limit resolution is maintained is smaller than the view field. Even in this case of reduced sampling, our CLASS microscopy has a strong benefit. After the completion of image acquisition, we can select a subimage whose area is set by the number of measured orthogonal modes, and then apply CLASS algorithm for the selected subimage. By performing this subimage correction for multiple isoplanatic patches in the original wide area image, we can ensure diffraction-limit spatial resolution over the entire view field. Indeed, the post-processing capability of CLASS microscopy works as a merit in this case.

In response to the reviewer's comment, we added the following sentence to the discussion section of the revised manuscript.

"In addition, due to the aberration correction for all the orthogonal modes within the view field, diffraction-limit spatial resolution can be obtained across the entire view field."

Reviewer #2

In the submitted manuscript the authors demonstrate a computational technique to mitigate the effect of sample induced aberrations in the context of their previously developed CASS technique. In their previous work (Kang et. al, Nat. Photon. 2015) the authors have demonstrated that the coherent summation of ballistic reflected light from a sample inside a scattering medium can lead to increased imaging quality in the presence of multiple scattered photons. However, the presence of surface aberrations together with the multiple light scattering would lead to the deterioration of the achieved image quality,

The novelty of the work relies in the addition of the computational step during the reconstruction process that allows for the cancellation of the phase retardations that the incoming and outgoing waves experience at the surface of the sample. Due to the coherent nature of the excitation and the detection the authors are able to post-hoc correct for these phase variations both under the excitation and detection light paths and increase the resulting image quality. They have demonstrated the applicability of their technique in artificial samples as well as ex vivo rabbit retina samples.

The manuscript is generally well written, the mathematical foundation of the technique is sufficiently documented and explained and the experimental results are convincing. Overall I consider the work an interesting enhancement of the previously developed technique with the potential to improve the imaging quality of coherent imaging methods.

We greatly appreciate the reviewer for acknowledging the novelty and importance of our work.

- Since the technique is a computationally enhancement of the previously developed method termed CASS, it will be limited by the same shortcomings. Mainly the applicability of the method would be rather limited to samples with a strong reflectivity. Could the authors add discussion of those shortcomings?

Like other coherent imaging methods working in the epi-detection geometry, the reflectivity of the sample is a source of contrast for CASS and CLASS microscopes. While this exempts the necessity of the labeling agents, which is critical for *in vivo* medical imaging, the CLASS microscope requires target samples to have sufficient reflectivity for its best performance in imaging depth. In fact, the maximum possible imaging depth is determined by the reflectivity of the sample as well as the scattering properties of the scattering medium. If the reflectivity of the sample is reduced by 1/10, then the imaging depth should be reduced by 1 scattering mean free path. We added the following sentences to the conclusion section of the revised manuscript.

"On the other hand, CLASS microscopy requires target samples to have sufficient reflectivity for its best performance in imaging depth similar to the other coherent imaging methods working in the epi-detection geometry. In fact, the maximum possible imaging depth is determined by the reflectivity of the sample as well as the scattering properties of the scattering medium. If the reflectivity of the sample is reduced by 1/10, then the imaging depth should be reduced by 1 scattering mean free path."

- In the part of the introduction where the authors communicate their method and place it with respect to the state of the art, I think that previous adaptive optics/wavefront correction studies could be better presented. Among the cited techniques some have been able to correct for very high order modes (pupil plane aberrations and scattering that far exceed the ones the

authors demonstrate in their figures) eg. references 17 and 19. Also, in the same context, other techniques (eg. Ji et. al, 2009, ref 15) could allow the correction of very steep phase gradients, similar to the ones demonstrated by the authors. Therefore the claim that the authors were able to correct aberrations that were previously outside the reach of adaptive optics is not accurate.

We agree with the reviewer, and added the following sentences to the revised manuscript to better introduce the performance of the previous adaptive optics and wavefront correction studies.

"In most cases, control of orthogonal modes such as Zernike Polynomials was used to deal with slowly varying aberrations in the pupil plane (Ref. 20). These so-called sensorless approaches usually require a large number of measurements for the experimental feedback control, and fluorescent molecules can be bleached during the process. But some of the approaches employed pixel-based wavefront shaping devices to compensate very steep phase gradient and high order modes of the aberrations (Refs. 15, 17) and introduced parallelization schemes to minimize the number of measurements (Ref. 19)."

- Can the authors comment on how the input and output pupil aberration maps (eg. the ones in Fig. 3) are related to one another? Please show the phase difference.

In the ideal case, the aberrations for the input and output should be identical in the reflection geometry since waves travel back and forth through physically the same sample. In the real practice, however, the optical system in the illumination beam path from the light source to the beam splitter and that in the collection beam path from the beam splitter to the camera are not perfectly the same. Therefore, input and output paths have different system aberrations. In addition, the slight misalignment of the optical axis or image focus can make the difference even larger. In general, the specimen-induced aberrations are much stronger than these system aberrations such that aberration maps for the input and output looked largely the same. For instance, the normalized cross-correlation of input and output pupil functions given by the aberration maps in Figs. 3i and 3j, respectively, was 0.64. But when we looked at the phase difference (Fig. R5) suggested by the reviewer, their difference was clearly visible. We could observe slowly varying phase difference across the pupil due to the difference of the system aberrations between the input and the output.

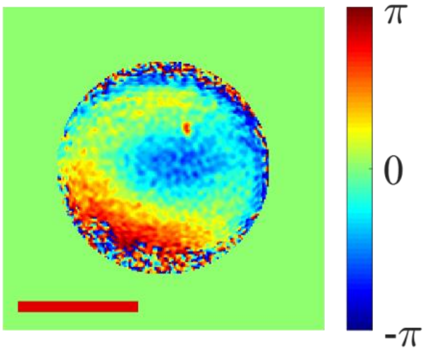

Figure R5. Phase difference between input and output aberrations obtained in Fig. 3. Scale bar, $k_0 \alpha$. Color bar, phase in radians.

Note that we also tried to correct aberration with the assumption that the input and output aberrations are identical. Figure R4 shows the identified aberration map and resulting CLASS image for the sample in Fig. 4. In comparison with Fig. 4c in the main text, the resolving power and signal intensity were significantly reduced. This result shows both the merit and the importance of independently addressing the input and output aberrations.

We added the following sentences to the revised manuscript and these new analyses to the Supplementary section III.7.

"The input and output aberration maps are largely the same as waves travel back and forth through physically the same sample in the reflection geometry. But due to the difference in the system aberrations of the illumination beam path from the light source to the beam splitter and the collection beam path from the beam splitter to the camera, they have a slight difference (see Supplementary section III.7)."

- In page 4, the authors write "Therefore, it is possible and in fact good enough to correct the aberration of illumination, especially when nonlinear processes are involved." It can be argued that the correction of aberration only in the illumination path will suffice only when nonlinear process are involved. One-photon wide-field and confocal imaging methods require the correction of the aberration in the detection path to render diffraction-limited images.

We appreciate the reviewer for elaborating the sentence. We revised the text in such a way to clarify the distinction between linear and nonlinear imaging modalities in adaptive optics.

"Therefore, it is possible to separately address the aberrations in the illumination and imaging paths, which is critical for one-photon wide-field and confocal imaging methods to render the diffraction-limited images. Imaging with nonlinear processes are even simpler since only the correction of aberration in the illumination beam path is sufficient."

- In the abstract, the use of the phrase "extremely shallow" could be avoided. I would suggest just "shallow" instead. Similarly, the phrase "ultra-high resolution imaging" in the title of the subsection is not quantitative. Consider revising.

We revised the text following the reviewer's suggestion.

Dear Editor

I'm in overall convinced by the response of the authors to my initial objections and the changes they made to the manuscript. Thus, I would favor publication in Nature Communications provided they address the comments I made in my reponse to their rebuttal letter. In particular, the definition of the Strehl ratio should be clarified (response to point 6) and more details should be provided about the experimental measurement of the scattering mean free path (response to comment 3).

Please find below my point-by-point reply to their response (in blue):

Reviewer #1

This study is devoted to optical imaging through thick scattering media. Conventional reflection imaging techniques, such as confocal microscopy or optical coherence tomography, suffer from the aberrations induced by the medium itself and from the multiple scattering events. Their approach is based on the experimental measurement of a time gated reflection matrix from the medium of interest. In a recent study published in Nature Photonics, the authors showed how to discriminate single scattering from the multiple scattering background from this reflection matrix [10]. In this paper, the authors extend this ds to be taken into account when they are compared.
ith the assumption that the input and output aberrations are identicajugation processes. They claim that they demonstrate "an enhancement of Strehl ratio by more than 1,000 times, two orders of magnitude improvement over conventional adaptive optics, and achieved the spatial resolution of 600 nm up to the unprecedented imaging depth of 7 scattering mean free paths.

Although the topic is of interest for applications to medical imaging and that the paper contains interesting results, I have several strong objections for publication in Nature Communications that are listed below.

We appreciate the reviewer for carefully reading our paper and raising critical concerns. While we think that the reviewer's opinions are highly valuable, we found that some of the concerns were overly stringent and some due to the confusion and misunderstanding. In the following, we addressed all the concerns raised by the reviewer in a point-by-point basis.

As a general comment, I would say that the authors tend to oversell the results of their work. To my opinion, the originality of this work lies in applying conventional adaptive optics tools to the reflection matrix approach of optical imaging that the authors and others have introduced in Refs.10 and 12.

We strongly disagree with the reviewer's opinion that our work is the adaptation of conventional adaptive optics tools to the reflection matrix approach. In our study, we

presented a novel adaptive optics method that enables us to independently identify the input and output aberrations from the elastically backscattered waves experiencing aberrations throughout their roundtrip to the target samples. This was done by the unique closed-loop optimization of the total intensity of the CLASS image for the forward and phase-conjugated processes, which has never been presented elsewhere. In the context of coherent imaging, the proposed method has significance in that it eliminates the needs for the guide stars and hardware-based iterative feedback control of the wavefront.

My criticism was not about the method (I said that it was original), but mainly about the claim made about the "unprecedented imaging of 7 scattering mean free paths".

Throughout the remarks, the reviewer compared ref. 12 with our present work. The work in ref. 12 is certainly interesting, but we think that its scope is completely different from ours. It presents an effective method for enhancing the imaging depth, but at the expense of spatial resolution. The researchers in ref. 12 applied a loose-confocal type filter to the reflection matrix through which single-scattered waves and a fraction of multiple-scattered waves are filtered. The effective diameter of the filter was either 5 or 8 μm, which is about 10 times larger than the diffraction-limit spatial resolution. Then they identified eigenchannels maximizing the total reflection intensity of the filtered single- and multiple-scattered waves, and reconstructed object images from these eigenchannels. This approach can be effective in enhancing signal strength due to the constructive interference of multiple scattering as well as single scattering, which led to the increased imaging depth. However, the same operation results in the reduction of the spatial resolution. Eigenchannels contain multiple-scattering information as well as singlescattering information, but multiple-scattering information was not used in such a way to keep track of the momentum change given by the target structure. Consequently, the spatial resolution presented in ref. 12 was a few microns, which is approximately the same as the effective diameter of the loose-confocal filter, even when the numerical aperture of the objective lens was 0.8.

Nowhere, I compared the results of the current paper with Ref.12. Ref.12 only tackles with the multiple scattering issue whereas the current paper mainly deals with the correction of aberrations. I just mentioned the supplementary of Ref.12 with regards to the Strehl ratio definition and the scaling of the SNR with this parameter.

For achieving the diffraction-limit spatial resolution, the image reconstruction should rely on the single-scattered waves, not the multiple-scattered waves, unless the trajectories of the multiplescattered waves are deterministically accounted for. In our study, we selectively optimized the diagonal elements of reflection matrix in the position basis for the image reconstruction. And the CLASS algorithm is designed to exclusively increase the intensity of single-scattered waves at the diagonal, not the multiple scattering background (see Fig. S6) for counteracting the specimen-induced aberration of the single-scattered waves. As such, we could achieve spatial resolution of 600 nm for 0.8 NA at the unprecedented depth 7 l_s *at such a high resolution. Indeed, our approach is designed to improve the imaging depth at which the diffraction-limit spatial resolution is maintained.*

See my response further

Although the experimental results they show are interesting, I am very skeptical about the claim they make about the multiplication of the Strehl ratio by a factor >1000 (I will explain

why below – see in particular my points 6 and 7). Their claim about the "unprecedented imaging depth of 7 scattering mean paths" with a diffraction limited resolution is in contradiction with the claim in Ref.10 that a diffraction-limited resolution was obtained through 11.5 scattering mean free paths.

The reviewer's concern about our claim of Strehl ratio enhancement is mainly due to the confusion about the definitions of Strehl ratio S and the single-scattering enhancement factor η. We clarified them when we address the reviewer's points #6 and #7.

Regarding the concern that our claim of imaging depth is contradictory to our previous paper, we would like to stress that it is meaningless to talk about imaging depth without considering the spatial resolution. For example, the medical ultrasound imaging can see deeper than any other optical imaging methodologies, but its spatial resolution is far worse than optical imaging. In our previous study, we demonstrated imaging depth of 11.5 l_s *for the numerical aperture (NA) of 0.4. The corresponding diffraction-limit spatial resolution was about 1.2 μm. On the other hand, we achieved spatial resolution of 600 nm in the present study, which is the diffractionlimit resolution set by 0.8 NA objective lens, to the imaging depth of 7* l_s*. Although 7* l_s *is shallower than 11.5* l_s*, this is an unprecedented imaging depth where the coherent imaging works at such a high resolution. Considering both imaging depth and spatial resolution altogether, our claim is not contradictory at all to the previous publication.*

It is not a straightforward task to maintain the diffraction-limit spatial resolution up to high numerical aperture. High spatial frequency components are much more prone to the sample induced aberrations than low spatial frequency components, and the correction of aberration becomes essential for achieving the diffraction-limit resolution. For a heuristic purpose, we present in Fig. R1 a plot of S and η for different NA values for the case of spherical aberration induced by a 1 mm-thick PDMS layer. The effect of aberration rapidly increases as the NA exceeds 0.5. The Strehl ratio and η were about 0.9 for 0.4 NA, which indicates that the effect of aberration was negligible at the condition of Ref. 10. But for 0.8 NA, these values were reduced to about 1/50 and 1/20, respectively. Moreover, we introduced astigmatic aberrations (Fig. 1) in addition to the spherical aberration by 7 l_s *thick PDMS layer, which further attenuated S to about 1/1300 and η to about 1/200. Consequently, the effect of aberration in our current experiment was much more severe than Ref. 10, and the correction of aberrations has been critical.*

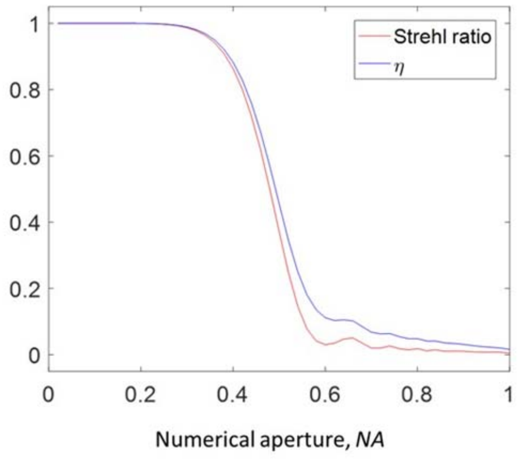

Figure R1. *Strehl ratio S and the single-scattering enhancement factor η depending on the numerical aperture for spherical aberration induced by 1mm-thick PDMS layer*

I fully agree with the authors that the aberration level is more important at high NA. However, my point was about the fact that the optical thickness (ratio between the thickness and the scattering mean free path) is an indicator of the multiple scattering level and not of the aberration level. The single-to-multiple scattering ratio is relatively constant with the NA (at least between NA=0.4 and 0.8, see Supplement of Ref.12 – sorry to refer again to it!). Hence, I don't think the optical thickness (unlike the Strehl ratio) is a relevant parameter for the current paper since the aberration level can be completely different according to the medium under investigation and imaging system even if the imaging depth is the same.

1. Several times in the paper, the authors say that their method works in a "strong multiple scattering regime". To my opinion, this claim is exaggerated and in contradiction with the results they present. Their claim is based on the weak signal-to-noise ratio (or rather the single-to-multiple scattering ratio) they observe in the wavevector basis (pupil plane). However, such a ratio should be investigated in the real basis because this is in this basis that the image of the sample is built.

We conducted image acquisition by choosing the diagonal elements of reflection matrix in the real basis. And the initial single-to-multiple scattering ratio was well below unity even in the real basis because sample-induced aberrations significantly attenuated the single scattering intensity. Therefore, it is legitimate to claim that our method works at the strong multiple scattering regime. We quantitatively addressed this reviewer's concern about the expression, 'strong multiple scattering regime,' in response to reviewer's points #2, #7, and #10.

Here, we clarify the reason why we use the wavevector basis as a starting point rather than the real basis. If we consider only the multiple scattering, the choice of basis is not important. As presented in Figs. 2c-d, both bases can be transformed into one another by means of Fourier transform or inverse Fourier transform. In the presence of aberration, however, the wavevector basis has a direct relevance. Aberration is the angle-dependent phase retardation

of the singlescattered waves. This means that we should correct aberration in the angular basis (or equivalently the wavevector basis) to counteract the phase retardation at each wavevector. This is the reason why most of the adaptive optics techniques put wavefront correction device at the pupil plane. Likewise, we applied aberration correction in the wavevector basis in such a way to maximize the total intensity in the diagonal components of the real basis.

I agree with the authors that they should also provide this SNR in the wavevector basis because it is where their correction takes place. However, I still think it is important to provide it also in the imaging plane for the reasons mentioned in my last review. I suggest to provide the SNR in each basis.

Typically, even at shallow depth (1 scattering mean free path), the single-to-multiple scattering ratio might be quite low in the wavevector basis but becomes very high in the imaging plane (Ref.12). Whereas single scattering signal spreads over all the elements of the reflection matrix in the wave vector basis, they only emerge along the close-diagonal elements of the reflection matrix in the real space, thus increasing the signal-to-noise ratio by a factor N_m, as stated in the manuscript. Fig.2d of the current ms shows the reflection matrix in the real basis. It is almost diagonal meaning that most of the recorded signal corresponds to single scattering. In a strong multiple scattering regime, this matrix would be, on the contrary, fully random (see for instance Ref.12). Hence, the authors cannot say that they are in a strong multiple scattering regime.

Even after the application of time-gated detection, the initial single-to-multiple scattering ratio at the diagonal of real basis in Fig. 2d was well below unity. Therefore, it is fair to state that the imaging condition was in strong multiple scattering regime. We are not sure why the strong multiple scattering regime should be defined by the condition that multiple scattering fully cover all the off-diagonal components of reflection matrix in the real basis. We think that what matters most is whether the initial SNR of imaging is well below unity or not.

In Figs. 2c and 2d, we deliberately reduced the spatial frequency bandwidth to $|k| < 0.26k_0$ to better visualize the matrix elements in the wavevector basis. But this led to the dominant display of the close-diagonal elements in Fig. 2d as the reviewer observed. From the reviewer's comment, we realized that it is more important to show the spread of diagonal elements in the real basis. Therefore, we present original matrices with the full spatial frequency bandwidth of $0.8k_0$ in Fig. R2 below. As shown in the figure, the single-scattered waves were significantly spread to off-diagonal elements due to the strong aberrations, and strong speckled multiple-scattered waves were superimposed on the top of these single-scattered waves. And the rest of the multiple-scattered waves were distributed across the whole plane.

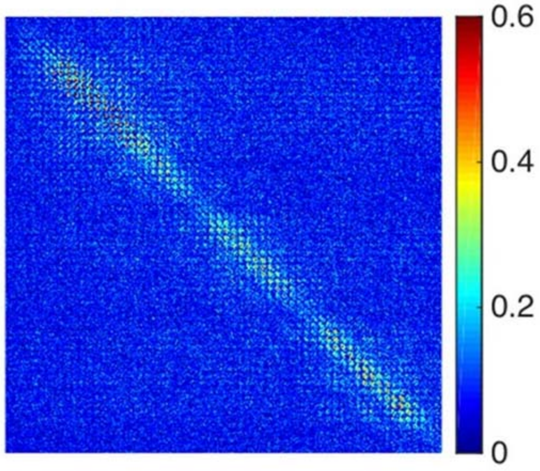

Figure R2. *Full data set of time resolved reflection matrix ; , in Fig. 2. Color bar, amplitude in arbitrary unit.*

In response to the reviewer's comment, we replaced Fig. 2c with the reflection matrix in the wavevector basis covering full 0.8 NA and Fig. 2d with Fig. R2. And we added the following sentence to the main text for the description of this new Fig. 2d.

"In Fig. 2d, we can observe that the single-scattered waves were significantly spread to off-diagonal elements due to the strong aberrations, and strong speckled multiple-scattered waves are superimposed on the top of these single-scattered waves."

The changes in Fig.2 are welcome to illustrate the effect of aberrations on the reflection matrix (spreading over off-diagonal elements). The misunderstanding with the authors may come from the discrimination between aberration and multiple scattering. Multiple scattering can arise from photons that are backscattered from the target layer (snake photons) and from the bulk tissue (see for instance G. Yao, L. V. Wang, Phys. Med. Biol. 44, 2307-2320, 1999). The first contribution can be seen as a sample-induced aberration and its energy will be centered around the diagonal of the reflection matrix in the real basis. The second contribution fully randomizes the direction of the incident light propagation and thus manifests itself as a homogeneous background on the reflection matrix. A strong multiple scattering regime corresponds to the case when the second contribution is larger than the first one: most of the collected photons are reflected by the bulk tissue and does not reach the target layer. In the case of Fig.2, this is not the case. That's why I claim that this experiment is not made in a strong multiple scattering regime and that I would remove the adjective "strong" before "multiple scattering".

To be more quantitative: In the Supplementary material, the authors say that their approach is valid for a ratio $\beta/(\eta\gamma)$ <16000. Given the fact that $\eta = 1/400$ in their simulation, this means that this is valid for a multiple-to-single scattering ratio $\beta/\gamma < 40$ in the wave vector basis. In the imaging plane, the single-to-multiple scattering limit ratio scales as $N_m \gamma/\beta$, with $N_m = 2800$. Hence the single-to-multiple scattering ratio is of 70 in the imaging

plane. We far from a strong multiple scattering regime where this ratio should be much smaller than 1…

If there is no aberration, the single-to-multiple scattering ratio should be $N_m\,\gamma/\beta$ *=70 in the real basis, and target samples must have been readily resolved even without the use of CLASS algorithm. However, this estimation is not valid anymore when there exist aberrations. The single-to-multiple scattering ratio is reduced by* η *due to the aberration, and the initial ratio is reduced to* $(\eta\gamma)/\beta$ *=70/400 =0.175<1 even in the real basis. In other words, multiple scattering dominates single scattering in the real basis because the single scattering signal is attenuated by the aberration. This is the reason why the target samples were invisible before the application of CLASS algorithm. Considering this initial condition, it is legitimate to state that CLASS algorithm has dealt with both strong multiple scattering and aberration.*

For the experimental data shown in Fig. 3, the initial single-to-multiple scattering intensity ratio was estimated to be $(\eta\gamma/\beta)N \simeq 0.1$*. Therefore, we added the following sentence to the revised manuscript.*

"Further analysis revealed that the initial single-to-multiple scattering intensity ratio was $\eta(\gamma/\beta)N_m \simeq 0.1$ *(see detailed analysis for Supplementary section III. 6)."*

I'm OK with the added sentence and the added supplementary section, however note that $(\eta\gamma)/\beta$ =0.175 should be rounded to 0.2, not to 0.1.

We agree on the fact that "multiple scattering dominates single scattering in the real basis because the single scattering signal is attenuated by aberration". However, this does not mean that we are in a strong multiple scattering regime (cf my previous point)

2. Related to my previous point, I think the title of this article is misleading. There is no suppression of scattering in this article. There is no multiple scattering contribution to remove as its contribution is already very weak in the original time gated confocal image. The CLASS microscopy enhances the single scattering contribution by correcting aberration but, by no means, removes multiple scattering.

Once again, the reviewer's judgement excluded the effect of aberration. As we responded to the reviewer's point #1, our experimental condition is such that the combined effect of multiple scattering and aberration attenuated the initial SNR well below unity even in the real basis, i.e. $\eta(\ \ /\ \ )$ *<1. Our CLASS algorithm resolves both the degradation of SNR due to multiple scattering and reduction of spatial resolution due to aberration by raising . Therefore, we think that the title of the article is relevant.*

I acknowledge that my initial remark was a bit stringent (of course, there is multiple scattering in the initial image). However, I still think that the "scattering suppression" claim made in the title of the article is not correct. The method proposed by the authors enables to correct for aberrations. Admittedly, this leads to an increase of the single-to-

multiple scattering intensity ratio but, strictly speaking, there is no multiple scattering removal here. The single scattering intensity is enhanced relatively to the multiple scattering background. I suggest to change the title into: "Label-free and high-resolution optical imaging within thick scattering media by suppression of aberration" or something equivalent. This change would not detract from the quality of the paper but would provide a more accurate description of the paper.

3. With regards to the imaging depth limit, the authors claim their approach allows to reach the "unprecedented imaging depth limit of 7 scattering mean paths". Besides the fact that this penetration depth may not be unprecedented (see above), the manuscript do not say how they measured the scattering mean free path in their samples. In the first experiment (Figs.1,2,3), they say that they place a 7ls thick scattering layer underneath the aberrating layer. But they do not provide any detail on this scattering layer and do not say how the scattering mean free path was measured. In the second experiment (Fig.4 – rat brain tissue), they say that the scattering mean free path was approximately of 100 µm. How did they measure it?

We appreciate the reviewer for pointing this out. We placed a stack of phantom layers of known thickness on a flat mirror, and then measured the decay of the intensity of ballistic components depending on the number of phantom layers. Specifically, we illuminated a normally incident plane wave and recorded the complex field map of the backscattered waves. We then extracted the intensity of the normally reflecting plane wave component from the recorded map. By using the exponential curve fitting, we could obtain the decay constant of 51.2µm for the roundtrip (Fig. R3). Therefore, the scattering mean free path of the scattering layer was 102.4 µm.

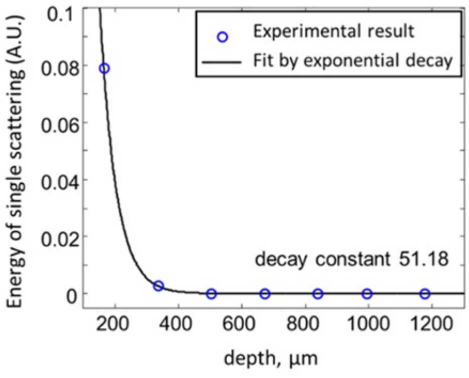

***Figure R3**. Calibration of scattering mean free path*

In the case of rat brain tissues, scattering properties of the tissue slices vary with imaging position. Therefore, we estimated the scattering mean free path in situ from the total intensity of the CLASS image, which is close to the intensity of the ballistic photons, and the thickness of the tissue layer measured from the time delay. We added these details about measuring the scattering mean free path to the section III.8 of SI.

Thanks for adding this new figure in the Supplementary. Nevertheless, it would be more convenient to have Fig.R3/S17 in log-scale as an exponential fit is done here. It would show the agreement between the experimental data and the fit at large probing depths. Moreover, I have one question about this way of measuring the scattering mean free path: The backscattered intensity should also contain a multiple scattering contribution (particularly at large depths)? How do the authors discriminate single and multiple scattering in that case? The presence of multiple scattering in their data could lead to a wrong estimation of the multiple scattering intensity. I think it is important to clarify that point before publication.

With regards to the brain tissues, I don't understand exactly how the authors measure the scattering mean free path from one intensity measurement. Do they normalize this measurement by a reference intensity backscattered by a mirror in free space?

4. With regards to their CLASS algorithm, the authors say that they perform "a closed-loop optimization of signal waves for both forward and phase-conjugation processes. " I do not understand the interest of the phase conjugation step in their algorithm. I'm ok with the first step where they apply a correction on the reflection matrix at the input based on a conventional maximization of the single scattering intensity of the CLASS image. Then, the authors use a second step where they take the transpose conjugate of the reflection matrix (phase conjugation operation) and then apply a correction at the output still based on a maximization of the single scattering intensity. And finally iterate this process. I do not understand here the interest of the phase conjugation operation. Without the phase conjugation operation, it should be possible to correct for aberrations, first at the input, then at the output (by just transposing the reflection matrix), and iterate the process to determine the input and output phase laws that compensate for the aberrations. Could the authors comment on that aspect? Maybe, they could show how the algorithm would work without the phase conjugation operation.

As the reviewer conjectured, the complex conjugation operation is not critical from the mathematical point of view in the second step of CLASS algorithm. It doesn't make any difference in the result except for the sign of the output phase correction map. What is important is the transpose operation, which enables us to swap input and output, and to correct the specimen-induced aberration from the output side. The reason we take phase conjugation (transpose + complex conjugation) rather than just the transpose is to impose a physical meaning to the operation. In this way, the second step is equivalent to physically sending the waves from the output side and measuring the backscattered waves at the input side.

We agree. It would be great to mention it in the paper.

5. Given the experimental set up used in their work (FigS7), the input and output arms look identical in terms of optical paths. Hence, by virtue of spatial reciprocity, the aberration phase law should be the same at the input and the output. Could the authors comment on

the fact that the aberration phase laws they experimentally determine with their algorithm look quite different at the input and output (see for instance Fig4 d and e)?

As the reviewer mentioned, the aberrations for the input and output should be identical in reflection geometry due to the spatial reciprocity. In the real practice, however, the optical system in the illumination beam path from the light source to the beam splitter and that in the collection beam path from the beam splitter to the camera are not perfectly the same. In addition, there may be slight misalignment of optical axis and image focus between the two beam paths. Therefore, the input and output can have different system aberrations. But these system aberrations are relatively smaller than the specimen-induced aberrations, and the aberration maps for the input and output are largely similar. For example, the normalized cross-correlation of the complex pupil functions of the aberration maps shown in Figs. 3i and 3j was about 0.64. In the case of the correlation between the maps in Fig. 4d and 4e, their correlation was still 0.46, but a bit lower than the phantom case since the magnitude of sample-induced aberrations was smaller. Note that the coordinate systems are flipped between the input and the output, and this needs to be taken into account when they are compared.

We tried to correct aberration with the assumption that the input and output aberrations are identical. Figure R4 below shows the identified aberration map and resulting CLASS image for the sample in Fig. 4. In comparison with Fig. 4c in the main text, the resolving power and signal intensity were significantly reduced. This result shows the importance of independently addressing the input and output aberrations.

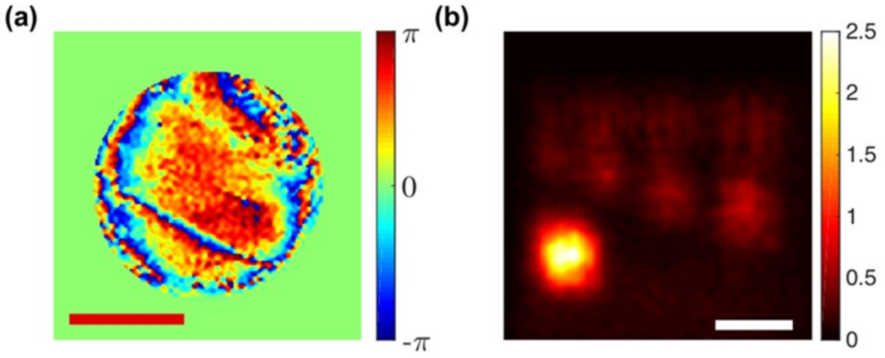

Figure R4. *Aberration correction of the data in Fig. 4 with the assumption that the input and output aberrations are identical.* ***a,*** *Acquired aberration map under the assumption of identical input-output aberration. Scale bar, .* ***b,*** *CLASS image after the aberration correction by* ***a****. Scale bar, 4 microns. Color bar, the intensity normalized by the maximum intensity in Fig. 4b.*

We added the following sentences to the revised manuscript and these new analyses to the Supplementary section III.7.

"The input and output aberration maps are largely the same as waves travel back and forth through physically the same sample in the reflection geometry. But due to the difference in the system aberrations between the illumination beam path from the light source to the beam splitter and the collection beam path from the beam splitter to the camera, they have a slight difference (see Supplementary section III.7)."

I'm OK with the authors' explanations and changes made to the manuscript.

6. I am very skeptical with regards to the claim made by the authors in the abstract that the Strehl ratio is increased by a factor >1000 with the CLASS algorithm.

In the first experiment described in the paper, the authors say that "the signal intensity was increased in magnitude by more than 20 times" (p13, line 295). A bit further, they claim that "The initial Strehl ratio S calculated from the acquired aberration maps was 1/1300" (p14, line 310). This is highly contradictory. After correction of aberrations, the single scattering intensity should be increased by a factor $1/S^2$ ***(the power 2 is due to the fact that aberrations are corrected both at the input and output, see for instance the Supplementary Material of Ref.12). Hence it means that the initial Strehl ratio should be*** $S = 1/\sqrt{20} = 0.22$. ***Hence, there is here a big contradiction with their own estimation of the Strehl ratio. How do they estimate it? Details about the estimation of the Strehl ratio are not provided.***

There is the same contradiction for the second experiment. The signal intensity is increased by a factor 16 (Fig.4), which yields an estimation of the Strehl ratio of 0.25 whereas the authors claim a Strehl ratio of 1/740 (p16, line 363).

At first, we clarify the definitions of S and η, and then discuss their physical meaning. In fact, the definitions of S and η, and the way they were estimated were described in full detail in the section I.6 of the previous SI (II.5 in the revised SI). In adaptive optics, S is defined by the peak intensity of the point-spread-function (PSF) in the presence of aberration relative to that without aberration. For the case when both the input and output aberrations matter, this parameter is written as

$$S = \left|\langle \mathcal{A}(\vec{k} = 0)\rangle\right|^2 / \left|\langle \mathcal{A}_0(\vec{k} = 0)\rangle\right|^2. \qquad \text{(R1)}$$

Here $\mathcal{A} = P_i^{a^*} \star P_o^a$ is the amplitude transfer function combining both input and output aberrations and $\mathcal{A}_0(\vec{k})$ is the ideal amplitude transfer function.

On the other hand, the factor η in Eq. (3) is defined by the ratio of the 'total' intensity, not the peak intensity, of the PSF in the presence of aberration with respect to that in the absence of aberration. In terms of the amplitude transfer function, η is written as

$$\eta = \langle |\mathcal{A}(\vec{k})|^2\rangle / \langle |\mathcal{A}_0(\vec{k})|^2\rangle. \qquad \text{(R2)}$$

Due to these differences in the definition, the relation $S^2 = \eta$ or $S = \eta$ that the reviewer conjectured doesn't exist. Certainly, S and η are related to a certain extent. The increase of aberration accompanies the simultaneous reduction in the peak intensity and the total intensity of the PSF. However, the peak height is more susceptible than the total intensity to the aberration. Therefore, S is usually smaller than η. But their relation depends on the type of aberration, which makes it difficult to find a general relation. For this reason, we only plotted S and η for one representative example of aberration in Fig. S6 (Fig. S7 in the revised SI).

Let us discuss the physical meaning of S and η. S is a parameter used to describe the aberration-induced signal reduction in the confocal and two-photon microscopes. It is a directly relevant parameter to the general adaptive optics audience because it indicates the degree of aberrations of the samples used in our study.

On the other hand, η describes the reduction of single-scattering intensity in the CLASS microscopy where images are acquired over wide area. Unlike confocal microscopy where signal is collected only at the confocal pinhole, the entire PSF contributes to the signal intensity in the wide-field detection. Therefore, the degradation of SNR in the CLASS microscope is determined by η, not by S. Likewise, the main role of CLASS algorithm is to increase single scattering intensity by $1/\eta$, not by $1/S$.

Now let's go back to the reviewer's comment. When we described the enhancement of signal intensity by either 20 times or 16 times, it is related to the increase of η, not S (the exact relation between 20 times and η is given below in response to reviewer's point #7). Therefore, the reviewer's estimation to deduce S from the signal enhancement is not relevant. Once again, both S and η are determined by the aberrations, but they are not equal.

First the expression of S (Eq.S15) has changed since the last version of the manuscript. It used to be:

$$S = \left|\langle \mathcal{A}(\vec{k})\rangle\right|^2 / \left|\langle \mathcal{A}_0(\vec{k})\rangle\right|^2. \qquad \text{(T1)}$$

And now it is

$$S = |\langle \mathcal{A}(\vec{k} = 0)\rangle|^2 / |\langle \mathcal{A}_0(\vec{k} = 0)\rangle|^2 . \quad \text{(T2)}$$

According to me, there is a problem with this expression of S. The PSF and the amplitude transfer function $\mathcal{A}(\vec{k})$ are related by a Fourier transform. The peak height of the PSF (i.e the PSF at the origin) is thus proportional to $\langle \mathcal{A}(\vec{k})\rangle$. As said in the manuscript, the Strehl ratio is defined by the peak intensity of the point-spread-function (PSF) in the presence of aberration relative to that without aberration. Hence, according to me, S is given by Eq.T1 and not by Eq.T2. The authors should clarify that point. Which equation do the authors use to estimate the Strehl ratio (Eq.T1 or T2?)

Otherwise, I find the physical picture the authors provide for the relation/difference between S and η in their rebuttal letter enlightening. I think this should be included in the Supplementary Section II.5.

7. Related to my last point: In the Supplementary material, the authors discuss the relation between the Strehl ratio S and η, which is the ratio of the total intensity of single-scattered waves in the presence of aberration with respect to that in the absence of aberration. For the authors, S and η should be the same for moderate aberrations. According to me, the scaling should be as $\eta = S^2$ (see my previous point - aberrations at input and output). But, even if we consider this scaling as correct, there is still a contradiction in their experiments. For the first experiment, the increase of the single scattering intensity is of 1/η=20 whereas they estimate 1/ S=1300. According to Fig.S6(b), for 1/η=20, 1/S and 1/η should be very close. The authors should absolutely clarify that point.

As we clarified in the comment #6, the relation, $S^2 = \eta$ or $S = \eta$ that the reviewer conjectured doesn't exist.

Now let us clarify η in Fig. 3. Indeed, we wrote in the main text that "Moreover, the signal intensity was increased in magnitude by more than 20 times, suggesting that the cross-correlation of the aberration-corrected pupil functions had been increased". Here the 20 times increase in signal intensity was estimated by comparing the intensity at the target in Fig. 3d and that in Fig. 3l. But this doesn't mean that η was 1/20. In fact, the real η that we estimated by applying Eq. (R2) to the measured aberration maps shown in Figs. 3i and 3j was $\eta = 1/204$ ($S = 1/1300$).

There is a discrepancy between the apparent signal enhancement of about 20 times estimated from Figs. 3d and 3l and the increase of single scattering intensity by $1/\eta$~200. This was mainly because multiple-scattered waves as well as single-scattered waves contributed to the signal intensity at the target in Fig. 3d. Before the application of CLASS algorithm, the intensity of CLASS image at the target is the sum of single-scattered waves and the multiple-scattered waves that survived the time-gating as well as spatial coherence gating, i.e. $I_{before} = I_{single} + I_{multi}$ with initial SNR given by $I_{single}/I_{multi} = (\eta\gamma/\beta)N_m$. After the aberration correction, the intensity at the target becomes, $I_{after} = I_{single}/\eta + I_{multi}$ with SNR increased to $(\gamma/\beta)N_m$.

Since the enhancement of the apparent signal intensity at the target was about 20 times, we can set up the following equation,

$$\frac{I_{after}}{I_{before}} = \frac{I_{single}/\eta + I_{multi}}{I_{single} + I_{multi}} = 20. \quad \text{(R3)}$$

By inserting the estimated η from the measured aberration maps, we can obtain the initial single-to-multiple scattering ratio in the position basis,

$$\frac{I_{single}}{I_{multi}} = (\eta\gamma/\beta)N_m = \frac{19}{184} \simeq 0.1.$$

Therefore, multiple scattering was about 10 times stronger than the single scattering before the aberration correction, and this was responsible for the discrepancy mentioned above. Since the initial SNR of $(\eta\gamma/\beta)N_m \simeq 0.1$ was far less than unity, multiple scattering has strongly degraded the resolving power of imaging. By the aberration correction, this SNR was increased to $(\gamma/\beta)N_m \simeq 21.1$ by selectively enhancing the intensity of single-scattered waves.

Regarding the discussion on Fig. S6(b) (Fig. S7(b) in the revised SI), S and η were acquired for the numerically assumed aberrations shown in Figs. S1a and S1b in the revised SI. For these

aberrations, $S = 1/3600$ at $\eta = 1/400$. In the case of experimental result shown in Fig. 3, $S = 1/1300$, and $\eta = 1/204$. Although the exact relation between S and η varies depending on the type of aberrations, the relative values of S to η in simulations and experiments are reasonably similar.

We added the following sentences to the revised manuscript to respond to all the issues raised by the reviewer. And the detailed discussion given above is added to the section III.6 of the revised SI.

"Moreover, the signal intensity at the target estimated from Fig. 3d and 3l was increased in magnitude by more than 20 times, suggesting that the cross-correlation of the aberration-corrected pupil functions had been increased."

"From the acquired angle-dependent phase correction maps in Figs. 3i and 3j, the initial Strehl ratio (see Supplementary section II.5 for the definition) was estimated to be $S = 1/1300$. This is two orders of magnitude smaller than the conventional adaptive optics typically handles. We also obtained $\eta = 1/204$ from Eq. (3) and measured aberration maps, which means that the total single scattering intensity was increased by about 200 times after the application of CLASS algorithm. There was a discrepancy between the apparent signal enhancement of about 20 times estimated from Figs. 3d and 3l and the increase of single scattering intensity by 200 times. And this was mainly because multiple-scattered waves as well as single-scattered waves contributed to the signal intensity at the target in Fig. 3d. Further analysis revealed that the initial single-to-multiple scattering intensity ratio was $\eta(\gamma/\beta)N_m \simeq 0.1$ (see detailed analysis for Supplementary section III. 6)."

I am convinced by the authors' reply and changes made in the manuscript and supplementary.

Related to this relation between S and , I don't understand why they should diverge at high aberration level. The explanation provided by the authors at page 11 of the supplementary material is not clear. Moreover, this in complete contradiction with Eqs.S15 and S16 that show an exactly similar expression for S and .

As we clarified in the response to the reviewer's point #6, ≠ from the definitions shown in Eqs. S15 and S16. The discrepancy between S and increases with the increase of the degree of aberration as the peak height of the PSF is more susceptible than its total intensity to the aberrations.

OK

8. Several times in the paper, the authors claim that "Typical adaptive optics can deal with aberrations for S >0.1". I think it would be relevant to provide at least one reference to corroborate that statement.

We appreciate the reviewer's suggestion, and added the following reference to the revised manuscript.

*X. Tao et al., Adaptive optics confocal microscopy using direct wavefront sensing, Optics Letters **36,** 1062 (2011).*

OK

10. In the last experiment shown in the paper, the authors do not discuss about the increase of the signal intensity and do not give any estimation of the Strehl ratio. Yet, in Fig.5, it seems that the signal intensity is only increased by a factor 2, which is one order of magnitude smaller than the previous experiments. Is it because there is not too much aberration in their sample or is this because the CLASS approach does not work so well in speckle (the object to image in the two first experiments is a resolution target)? This limited performance should be discussed.

The apparent signal increase at the fungal filament was about 9.05 times, not 2 times in Fig. 5h. In fact, the images were a bit saturated for better visualization of the fungal structures across the entire view field. Signal enhancement was smaller than the phantom case, but not by an order of magnitude.

As discussed above, this apparent signal increase is not equal to the enhancement of single scattering intensity. From the obtained aberration maps of the output (Fig. 5j) and input (not shown in the figure), we obtained S=1/437 and η=1/44.7. The initial single-to-multiple scattering ratio was $(\eta\gamma/\beta)N \sim 0.23$ *from the same analysis given to the reviewer's point #7.*

For the case of the specimen in Fig. 5i, the enhancement of intensity was about 3.49 with S=1/890, η=1/68.2, respectively. With these parameters, the initial single-to-multiple scattering ratio was estimated to be about 0.04, which indicates that the biological specimens were under the influence of strong multiple scattering.

In imaging biological tissues, the single scattering intensity is inevitably smaller than the resolution target case. Therefore, the degree of multiple scattering and aberrations that can be overcome tends to be smaller. But what is important in assessing the performance of the imaging method is the initial single-to-multiple scattering ratio that the method can deal with. In this way, the performance can be characterized independent of the types of samples. In this respect, our CLASS microscope works equally well for biological tissues since we could handle initial SNR as small as 0.04 even for biological specimens.

In response to the reviewer's comment, we added S, η and initial SNR of the biological data to the revised manuscript. Also, the following discussion on the limited performance of CLASS microscope (in fact, all the coherent imaging method) for biological specimens was added to the conclusion section.

"On the other hand, CLASS microscopy requires target samples to have sufficient reflectivity for its best performance in imaging depth similar to the other coherent imaging methods working in the epi-detection geometry. In fact, the maximum possible imaging depth is determined by the reflectivity of the sample as well as the scattering properties of the scattering medium. If the reflectivity of the sample is reduced by 1/10, then the imaging depth should be reduced by 1 sc attering mean free path."

I am convinced by the authors' reply and changes made to the manuscript and supplementary.

11. In their conclusion, the authors say that one important aspect is that "aberration correction is performed over a wide field of view with finely stepped illumination angles". A larger field of view also implies the presence of several isoplanatic patches in the field of view. The aberration correction provided by the CLASS algorithm is limited to a field of

view that contains one single isoplanatic patch. The authors should mention it in their conclusion. It is important to mention the improvements provided by the CLASS approach but also its limits.

In our experiment, we could obtain the spatial resolution of 600 nm across the entire view field of $30 \times 30\ \mu m^2$ even at the severe aberrations shown in Fig. 3. This is mainly because we corrected aberrations for 2,800 wavevectors covering all the orthogonal modes given by the view field and numerical aperture. In fact, this is the maximum degree of correction that can be made for the given view field. As a consequence, the entire view field was seen as a single isoplanatic patch. Therefore, the CLASS algorithm is not limited to the field of view that contains one single isoplanatic patch, but enables us to treat the view field as a single isoplanatic patch by employing the maximum available degree of aberration corrections.

If the number of wavevectors is reduced by increasing the angular stepping, then the reviewer's reasoning is correct. In such case, the view field should be split into multiple isoplanatic patches, and corrections should be made to individual patches. In fact, this is the case for conventional adaptive optics. The number of modes that deformable mirrors can control is much smaller than the number of orthogonal modes set by the view field and the numerical aperture in ordinary adaptive optics. Because of this deficiency in the number of control, the area where the diffraction-limit resolution is maintained is smaller than the view field. Even in this case of reduced sampling, our CLASS microscopy has a strong benefit. After the completion of image acquisition, we can select a subimage whose area is set by the number of measured orthogonal modes, and then apply CLASS algorithm for the selected subimage. By performing this subimage correction for multiple isoplanatic patches in the original wide area image, we can ensure diffraction-limit spatial resolution over the entire view field. Indeed, the post-processing capability of CLASS microscopy works as a merit in this case.

In response to the reviewer's comment, we added the following sentence to the discussion section of the revised manuscript.

"In addition, due to the aberration correction for all the orthogonal modes within the view field, diffraction-limit spatial resolution can be obtained across the entire view field."

I'm not sure to fully agree with the authors: the high spatial frequencies of an aberrating layer are associated with a smaller isoplanatic patch. See for instance J. Mertz, H. Paudel, and T. G. Bifano, Appl. opt. 54, 3498, 2015 where it is shown that the size of one isoplanatic patch scales with the characteristic length scale of the phase variations induced by the aberrating layer. Reducing the angular stepping thus increases the size of the isoplanatic patches. Anyway, the field of view considered in that paper is relatively reduced, so this problem is probably out-of-the-scope of that paper. I was just curious about the way they could face this issue.

Reviewer #2 (Remarks to the Author):

The authors addressed most of the points that I raised during my first response. However, some additional issues raised by Reviewer #1 are valid and the authors' responses to those have created concerns on which I also comment below:

Regarding my question about the difference between the phase maps of the input and output aberrations (as also raised by Reviewer #1 in point 5) and Figure R5, it seems that the total difference between the two phase maps is dominated by spherical system aberrations. In that case, this isn't the best example to demonstrate the technique, unless the authors only want to claim the ability to correct system aberrations.

I think that there is some confusion introduced by the use of the term “single scattered waves” vs “multiple scattered waves” as used by the authors. By the term “single scattered waves” the authors mean the ballistic back-scattered waves from the target and not the waves that have been involved in any scattering events inside the medium. In the CASS technique, the authors accumulate this ballistic light and suppress the contribution of sample induced scattered light. Therefore, for the authors, any light that is not ballistic back-scattered by the object is “multiply scattered light” which I believe is misleading. Reviewer #1 raised an interesting point about the extent of the diagonal elements in real space. From that one can see that scattering seems to be dominated by “sample inhomogeneity single/few scattering events” and not by multiple scattering of light (elements close to the main diagonal), since multiple scattered light will be outside the time gate (due to a longer optical path inside the medium). Furthermore, the single to multiple scattered light ratio would be more correctly called ballistic to scattered light ratio and should not be confused with multiple scattering events.

The response of the authors to point #11 of Reviewer #1 is not clear and also raises a number of issues. My thoughts on the issue:
a. The number of wave vectors used to create the CASS image defines the maximum number of independent modes at the pupil plane that can be measured and is not related to the isoplanatic patch.
b. Based on the experimental setup and the way that the problem is formulated, the sample induced aberrations are treated as a single surface phase aberration layer that affects the incoming and outgoing waves all at the same time. As already discussed,
those should be the complex transpose of one another apart from system aberrations that might be different of the input and output arms. Therefore, the sample aberrations that are corrected form indeed a single isoplanatic patch.
c. The treatment of different isoplanatic patches would require the independent measurement of smaller spatially separated fields of view and the corresponding generation of different correction phase maps.

Very importantly, motivated by the points raised by Reviewer #1 regarding the Strehl ratio, my opinion is that the use of the Strehl ratio for the quantification of the aberrations in the current context is problematic and could easily lead to confusions. The authors have estimated the Strehl ratio from Equation R1 (also present in the Supplementary Material), which defines it as the ratio between the squared amplitude of the summed crosscorrelation between the input and output aberrations over the equivalent measure for a uniform pupil. Such a metric is equivalent to comparing the expected intensity at the center of the aberrated PSF against the intensity at the center of an ideal diffraction-limited PSF. Although this definition can be found in the literature, I think it could lead to an overestimation of the actual aberrations. Conventionally, when the ground-truth is not known, the Strehl ratio should be calculated by removing piston, tilt and defocus terms, since those are trivially corrected by the optical setup and do not lead to image degradation relative to the ideal diffraction

limited PSF. Even further, the Strehl ratio was conceived to quantify the deterioration of the image quality of an imaging system under the presence of aberrations. Since this metric is indeed what matters, I think that only the image intensity enhancement factors are the ones that reflect important information and are not misleading.

We greatly appreciate the reviewers for their detailed follow-up comments to our previous discussions and analyses. We have thoroughly addressed all the remaining concerns raised by the reviewers. In particular, we resolved the issues related to terminology by removing the adjective ‘strong’ in the strong multiple-scattered waves and changing the title of the manuscript in accordance with the reviewer’s suggestion. We also clarified the definition of the Strehl ratio, and estimated it again by applying the general practice used in the field of adaptive optics. Our response to the reviewers’ comments were written in purple in this response letter, and all the changes were highlighted in red in the revised manuscript. Overall, the reviewers’ comments were highly valuable, and our efforts to addressing them led to a scientifically more rigorous paper than before. With all these revisions, we now believe that our paper is ready for publication.

Reviewer #1

I’m in overall convinced by the response of the authors to my initial objections and the changes they made to the manuscript. Thus, I would favor publication in Nature Communications provided they address the comments I made in my response to their rebuttal letter. In particular, the definition of the Strehl ratio should be clarified (response to point 6) and more details should be provided about the experimental measurement of the scattering mean free path (response to comment 3).

Please find below my point-by-point reply to their response (in blue):

We greatly appreciate the reviewer’s favorable consideration of our explanations and analyses. In fact, the reviewer’s comments were all very helpful and constructive, and our paper has become scientifically more rigorous than before by addressing the issues raised by the reviewer. In this response letter, we clarified the definition of the Strehl ratio, the measurements of the scattering mean free path, and other miscellaneous issues.

This study is devoted to optical imaging through thick scattering media. Conventional reflection imaging techniques, such as confocal microscopy or optical coherence tomography, suffer from the aberrations induced by the medium itself and from the multiple scattering events. Their approach is based on the experimental measurement of a time gated reflection matrix from the medium of interest. In a recent study published in Nature Photonics, the authors showed how to discriminate single scattering from the multiple scattering background from this reflection matrix [10]. In this paper, the authors extend this work by dealing with the aberration correction. To do so, they perform a closed-loop optimization of singly-scattered waves for both forward and phase-conjugation processes. They claim that they demonstrate “an enhancement of Strehl ratio by more than 1,000 times, two orders of magnitude improvement over conventional adaptive optics, and achieved the spatial resolution of 600 nm up to the unprecedented imaging depth of 7 scattering mean free paths.

Although the topic is of interest for applications to medical imaging and that the paper contains interesting results, I have several strong objections for publication in Nature Communications that are listed below.

We appreciate the reviewer for carefully reading our paper and raising critical concerns. While we think that the reviewer’s opinions are highly valuable, we found that some of the concerns were overly stringent and some due to the confusion and misunderstanding. In the following, we addressed all the concerns raised by the reviewer in a point-by-point basis.

As a general comment, I would say that the authors tend to oversell the results of their work. To my opinion, the originality of this work lies in applying conventional adaptive optics tools to the reflection matrix approach of optical imaging that the authors and others have introduced in Refs.10 and 12.

We strongly disagree with the reviewer's opinion that our work is the adaptation of conventional adaptive optics tools to the reflection matrix approach. In our study, we presented a novel adaptive optics method that enables us to independently identify the input and output aberrations from the elastically backscattered waves experiencing aberrations throughout their roundtrip to the target samples. This was done by the unique closed-loop optimization of the total intensity of the CLASS image for the forward and phase-conjugated processes, which has never been presented elsewhere. In the context of coherent imaging, the proposed method has significance in that it eliminates the needs for the guide stars and hardware-based iterative feedback control of the wavefront.

My criticism was not about the method (I said that it was original), but mainly about the claim made about the "unprecedented imaging of 7 scattering mean free paths".

We appreciate the reviewer's acknowledgement of the originality of our work. We now fully understood the reviewer's original intention.

Throughout the remarks, the reviewer compared ref. 12 with our present work. The work in ref. 12 is certainly interesting, but we think that its scope is completely different from ours. It presents an effective method for enhancing the imaging depth, but at the expense of spatial resolution. The researchers in ref. 12 applied a loose-confocal type filter to the reflection matrix through which single-scattered waves and a fraction of multiple-scattered waves are filtered. The effective diameter of the filter was either 5 or 8 μm, which is about 10 times larger than the diffraction-limit spatial resolution. Then they identified eigenchannels maximizing the total reflection intensity of the filtered single- and multiple-scattered waves, and reconstructed object images from these eigenchannels. This approach can be effective in enhancing signal strength due to the constructive interference of multiple scattering as well as single scattering, which led to the increased imaging depth. However, the same operation results in the reduction of the spatial resolution. Eigenchannels contain multiple-scattering information as well as single-scattering information, but multiple-scattering information was not used in such a way to keep track of the momentum change given by the target structure. Consequently, the spatial resolution presented in ref. 12 was a few microns, which is approximately the same as the effective diameter of the loose-confocal filter, even when the numerical aperture of the objective lens was 0.8.

Nowhere, I compared the results of the current paper with Ref.12. Ref.12 only tackles with the multiple scattering issue whereas the current paper mainly deals with the correction of aberrations. I just mentioned the supplementary of Ref.12 with regards to the Strehl ratio definition and the scaling of the SNR with this parameter.

Once again, we think that Ref. 12 is an interesting and unique work that efficiently dealt with multiple scattering issue, and the discussion of Strehl ratio therein was helpful in analyzing our data. Together with the work we presented here, these studies will all together help advancing

the field of life science and biomedicine where deep-tissue and high-resolution imaging techniques are most needed.

For achieving the diffraction-limit spatial resolution, the image reconstruction should rely on the single-scattered waves, not the multiple-scattered waves, unless the trajectories of the multiple-scattered waves are deterministically accounted for. In our study, we selectively optimized the diagonal elements of reflection matrix in the position basis for the image reconstruction. And the CLASS algorithm is designed to exclusively increase the intensity of single-scattered waves at the diagonal, not the multiple scattering background (see Fig. S6) for counteracting the specimen-induced aberration of the single-scattered waves. As such, we could achieve spatial resolution of 600 nm for 0.8 NA at the unprecedented depth 7 l_s at such a high resolution. Indeed, our approach is designed to improve the imaging depth at which the diffraction-limit spatial resolution is maintained.

See my response further

Although the experimental results they show are interesting, I am very skeptical about the claim they make about the multiplication of the Strehl ratio by a factor >1000 (I will explain why below – see in particular my points 6 and 7). Their claim about the "unprecedented imaging depth of 7 scattering mean paths" with a diffraction limited resolution is in contradiction with the claim in Ref.10 that a diffraction-limited resolution was obtained through 11.5 scattering mean free paths.

The reviewer's concern about our claim of Strehl ratio enhancement is mainly due to the confusion about the definitions of Strehl ratio S and the single-scattering enhancement factor η. We clarified them when we address the reviewer's points #6 and #7.

Regarding the concern that our claim of imaging depth is contradictory to our previous paper, we would like to stress that it is meaningless to talk about imaging depth without considering the spatial resolution. For example, the medical ultrasound imaging can see deeper than any other optical imaging methodologies, but its spatial resolution is far worse than optical imaging. In our previous study, we demonstrated imaging depth of 11.5 l_s for the numerical aperture (NA) of 0.4. The corresponding diffraction-limit spatial resolution was about 1.2 µm. On the other hand, we achieved spatial resolution of 600 nm in the present study, which is the diffraction-limit resolution set by 0.8 NA objective lens, to the imaging depth of 7 l_s. Although 7 l_s is shallower than 11.5 l_s, this is an unprecedented imaging depth where the coherent imaging works at such a high resolution. Considering both imaging depth and spatial resolution altogether, our claim is not contradictory at all to the previous publication.

It is not a straightforward task to maintain the diffraction-limit spatial resolution up to high numerical aperture. High spatial frequency components are much more prone to the sample-induced aberrations than low spatial frequency components, and the correction of aberration becomes essential for achieving the diffraction-limit resolution. For a heuristic purpose, we present in Fig. R1 a plot of S and η for different NA values for the case of spherical aberration induced by a 1 mm-thick PDMS layer. The effect of aberration rapidly increases as the NA exceeds 0.5. The Strehl ratio and η were about 0.9 for 0.4 NA, which indicates that the effect of aberration was negligible at the condition of Ref. 10. But for 0.8 NA, these values were reduced to about 1/50 and 1/20, respectively. Moreover, we introduced astigmatic aberrations (Fig. 1) in

addition to the spherical aberration by 7 l_s thick PDMS layer, which further attenuated S to about 1/1300 and η to about 1/200. Consequently, the effect of aberration in our current experiment was much more severe than Ref. 10, and the correction of aberrations has been critical.

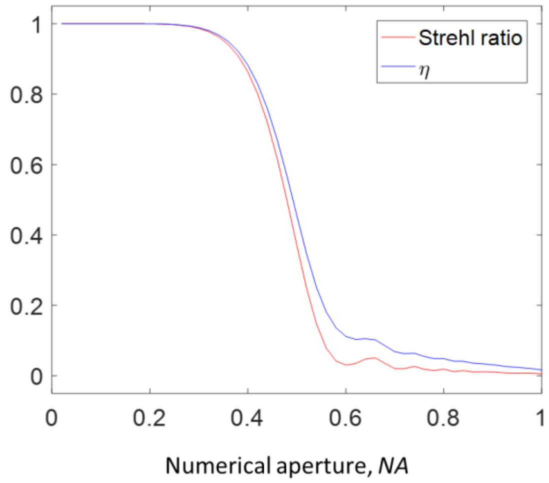

Figure R1. Strehl ratio S and the single-scattering enhancement factor η depending on the numerical aperture for spherical aberration induced by 1mm-thick PDMS layer

I fully agree with the authors that the aberration level is more important at high NA. However, my point was about the fact that the optical thickness (ratio between the thickness and the scattering mean free path) is an indicator of the multiple scattering level and not of the aberration level. The single-to-multiple scattering ratio is relatively constant with the NA (at least between NA=0.4 and 0.8, see Supplement of Ref.12 – sorry to refer again to it!). Hence, I don't think the optical thickness (unlike the Strehl ratio) is a relevant parameter for the current paper since the aberration level can be completely different according to the medium under investigation and imaging system even if the imaging depth is the same.

We agree with reviewer in that the key parameter in our study is η (or S) and the optical thickness of the scattering media may be a secondary parameter. But still, the optical thickness plays an important role as it affects to γ/β in the initial SNR, $(\gamma/\beta)\eta$. Therefore, we think it desirable to provide all these parameters for the precise description of the technical capability of CLASS microscope.

1. Several times in the paper, the authors say that their method works in a "strong multiple scattering regime". To my opinion, this claim is exaggerated and in contradiction with the results they present. Their claim is based on the weak signal-to-noise ratio (or rather the single-to-multiple scattering ratio) they observe in the wavevector basis (pupil plane). However, such a ratio should be investigated in the real basis because this is in this basis that the image of the sample is built.

We conducted image acquisition by choosing the diagonal elements of reflection matrix in the real basis. And the initial single-to-multiple scattering ratio was well below unity even in the real basis because sample-induced aberrations significantly attenuated the single scattering intensity. Therefore, it is legitimate to claim that our method works at the strong multiple scattering regime. We quantitatively addressed this reviewer's concern about the expression, 'strong multiple scattering regime,' in response to reviewer's points #2, #7, and #10.

Here, we clarify the reason why we use the wavevector basis as a starting point rather than the real basis. If we consider only the multiple scattering, the choice of basis is not important. As presented in Figs. 2c-d, both bases can be transformed into one another by means of Fourier transform or inverse Fourier transform. In the presence of aberration, however, the wavevector basis has a direct relevance. Aberration is the angle-dependent phase retardation of the single-scattered waves. This means that we should correct aberration in the angular basis (or equivalently the wavevector basis) to counteract the phase retardation at each wavevector. This is the reason why most of the adaptive optics techniques put wavefront correction device at the pupil plane. Likewise, we applied aberration correction in the wavevector basis in such a way to maximize the total intensity in the diagonal components of the real basis.

I agree with the authors that they should also provide this SNR in the wavevector basis because it is where their correction takes place. However, I still think it is important to provide it also in the imaging plane for the reasons mentioned in my last review. I suggest to provide the SNR in each basis.

We appreciate the reviewer's suggestion. In response to the reviewer's comments at the first round of review, we provided SNR in the image plane, $(\gamma/\beta)\eta$. From the estimated value of η calculated by the aberration maps, we could determine the SNR in the wavevector space, γ/β. The following sentence was added to the revised manuscript to provide SNR in each basis.

"Further analysis revealed that the initial single-to-multiple scattering intensity ratio of individual angle-dependent images was $\gamma/\beta \simeq 0.007$, and the initial single-to-multiple scattering intensity ratio of the reconstructed image was $\eta(\gamma/\beta)N_m \simeq 0.1$ (see detailed analysis for Supplementary section III. 6)."

Typically, even at shallow depth (1 scattering mean free path), the single-to-multiple scattering ratio might be quite low in the wavevector basis but becomes very high in the imaging plane (Ref.12). Whereas single scattering signal spreads over all the elements of the reflection matrix in the wave vector basis, they only emerge along the close-diagonal elements of the reflection matrix in the real space, thus increasing the signal-to-noise ratio by a factor Nm, as stated in the manuscript. Fig.2d of the current ms shows the reflection matrix in the real basis. It is almost diagonal meaning that most of the recorded signal corresponds to single scattering. In a strong multiple scattering regime, this matrix would be, on the contrary, fully random (see for instance Ref.12). Hence, the authors cannot say that they are in a strong multiple scattering regime.

Even after the application of time-gated detection, the initial single-to-multiple scattering ratio at the diagonal of real basis in Fig. 2d was well below unity. Therefore, it is fair to state that the imaging condition was in strong multiple scattering regime. We are not sure why the strong multiple scattering regime should be defined by the condition that multiple scattering fully cover all the off-diagonal components of reflection matrix in the real basis. We think that what matters most is whether the initial SNR of imaging is well below unity or not.

In Figs. 2c and 2d, we deliberately reduced the spatial frequency bandwidth to $|k| < 0.26k_0$ to better visualize the matrix elements in the wavevector basis. But this led to the dominant display of the close-diagonal elements in Fig. 2d as the reviewer observed. From the reviewer's comment, we realized that it is more important to show the spread of diagonal elements in the real basis. Therefore, we present original matrices with the full spatial frequency bandwidth of $|k| < 0.8k_0$ in Fig. R2 below. As shown in the figure, the single-scattered waves were significantly spread to off-diagonal elements due to the strong aberrations, and strong speckled multiple-scattered waves were superimposed on the top of these single-scattered waves. And the rest of the multiple-scattered waves were distributed across the whole plane.

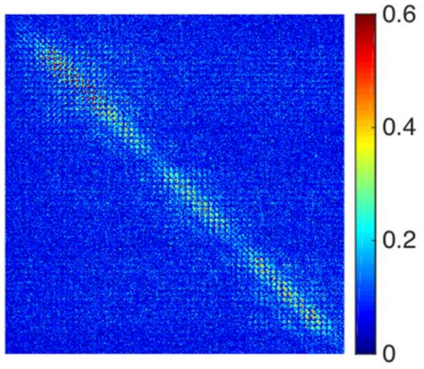

Figure R2. Full data set of time resolved reflection matrix $E\left(\vec{r}^{o};\vec{r}^{i},\tau_0\right)$ in Fig. 2. Color bar, amplitude in arbitrary unit.

In response to the reviewer's comment, we replaced Fig. 2c with the reflection matrix in the wavevector basis covering full 0.8 NA and Fig. 2d with Fig. R2. And we added the following sentence to the main text for the description of this new Fig. 2d.

"In Fig. 2d, we can observe that the single-scattered waves were significantly spread to off-diagonal elements due to the strong aberrations, and strong speckled multiple-scattered waves are superimposed on the top of these single-scattered waves."

The changes in Fig.2 are welcome to illustrate the effect of aberrations on the reflection matrix (spreading over off-diagonal elements). The misunderstanding with the authors may come from the discrimination between aberration and multiple scattering. Multiple scattering can arise from photons that are backscattered from the target layer (snake photons) and from the bulk tissue (see for instance G. Yao, L. V. Wang, Phys. Med. Biol. 44, 2307-2320, 1999). The first contribution can be seen as a sample-induced aberration and its energy will be centered around the diagonal of the reflection matrix in the real basis. The second contribution fully randomizes the direction of the incident light propagation and thus manifests itself as a homogeneous background on the reflection matrix. A strong multiple scattering regime corresponds to the case when the second contribution is larger than the first one: most of the collected photons are reflected by the bulk tissue and does not reach the target layer. In the case of Fig.2, this is not the case. That's why I claim that this experiment is not made in a strong multiple scattering regime and that I would remove the adjective "strong" before "multiple scattering".

We appreciate the reviewer's thoughtful comment. We considered that the thickness of $7l_s$ corresponds to the strong multiple scattering regime for high-resolution imaging aiming at spatial resolution of 600 nm. But we now acknowledge the reviewer's opinion on the convention of the 'strong' multiple scattering regime, and removed the adjective 'strong' in the strong multiple-scattered waves throughout the revised manuscript.

To be more quantitative: In the Supplementary material, the authors say that their approach is valid for a ratio $\beta/(\eta\gamma)$ <16000. Given the fact that η = 1/400 in their simulation, this means that this is valid for a multiple-to-single scattering ratio β/γ < 40 in the wave vector basis. In the imaging plane, the single-to-multiple scattering limit ratio scales as $Nm\ \gamma/\beta$, with Nm = 2800. Hence the single-to-multiple scattering ratio is of 70 in the imaging plane. This is far from a strong multiple scattering regime where this ratio should be much smaller than 1.

If there is no aberration, the single-to-multiple scattering ratio should be $N_m\ (\gamma/\beta)$ =70 in the real basis, and target samples must have been readily resolved even without the use of CLASS algorithm. However, this estimation is not valid anymore when there exist aberrations. The single-to-multiple scattering ratio is reduced by η due to the aberration, and the initial ratio is reduced to $(\gamma/\beta)\eta$ =70/400 =0.175<1 even in the real basis. In other words, multiple scattering dominates single scattering in the real basis because the single scattering signal is attenuated by the aberration. This is the reason why the target samples were invisible before the application of CLASS algorithm. Considering this initial condition, it is legitimate to state that CLASS algorithm has dealt with both strong multiple scattering and aberration.

For the experimental data shown in Fig. 3, the initial single-to-multiple scattering intensity ratio was estimated to be $(\eta\gamma/\beta)N_m \simeq 0.1$. Therefore, we added the following sentence to the revised manuscript.

"Further analysis revealed that the initial single-to-multiple scattering intensity ratio was $\eta(\gamma/\beta)N_m \simeq 0.1$ (see detailed analysis for Supplementary section III. 6)."

I'm OK with the added sentence and the added supplementary section, however note that $N_m(\eta\gamma)/\beta$=0.175 should be rounded to 0.2, not to 0.1.

$N_m(\eta\gamma)/\beta$ for the experimental data in Fig. 3 was indeed about 0.1 (Supplementary section III.6) and that for the numerical simulation was 0.175.

We agree on the fact that "multiple scattering dominates single scattering in the real basis because the single scattering signal is attenuated by aberration". However, this does not mean that we are in a strong multiple scattering regime (cf my previous point)

Following the reviewer's suggestion, we dropped the adjective 'strong' in the strong multiple-scattered waves in the revised manuscript.

2. Related to my previous point, I think the title of this article is misleading. There is no suppression of scattering in this article. There is no multiple scattering contribution to

remove as its contribution is already very weak in the original time gated confocal image. The CLASS microscopy enhances the single scattering contribution by correcting aberration but, by no means, removes multiple scattering.

Once again, the reviewer's judgement excluded the effect of aberration. As we responded to the reviewer's point #1, our experimental condition is such that the combined effect of multiple scattering and aberration attenuated the initial SNR well below unity even in the real basis, i.e. $\eta(\gamma/\beta)N_m$ <1. Our CLASS algorithm resolves both the degradation of SNR due to multiple scattering and reduction of spatial resolution due to aberration by raising η. Therefore, we think that the title of the article is relevant.

I acknowledge that my initial remark was a bit stringent (of course, there is multiple scattering in the initial image). However, I still think that the "scattering suppression" claim made in the title of the article is not correct. The method proposed by the authors enables to correct for aberrations. Admittedly, this leads to an increase of the single-to-multiple scattering intensity ratio but, strictly speaking, there is no multiple scattering removal here. The single scattering intensity is enhanced relatively to the multiple scattering background. I suggest to change the title into: "Label-free and high-resolution optical imaging within thick scattering media by suppression of aberration" or something equivalent. This change would not detract from the quality of the paper but would provide a more accurate description of the paper.

We used the expression, 'suppression of scattering,' in the sense that the enhanced single scattering by the aberration correction reduces the effect of multiple scattering. But we also think that the reviewer's suggestion is reasonable in the strict sense. We therefore modified the title of our paper to

"Label-free and high-resolution adaptive optical imaging within thick scattering media by the closed-loop accumulation of single scattering."

3. With regards to the imaging depth limit, the authors claim their approach allows to reach the "unprecedented imaging depth limit of 7 scattering mean paths". Besides the fact that this penetration depth may not be unprecedented (see above), the manuscript do not say how they measured the scattering mean free path in their samples. In the first experiment (Figs.1,2,3), they say that they place a 7ls thick scattering layer underneath the aberrating layer. But they do not provide any detail on this scattering layer and do not say how the scattering mean free path was measured. In the second experiment (Fig.4 – rat brain tissue), they say that the scattering mean free path was approximately of 100 µm. How did they measure it?

We appreciate the reviewer for pointing this out. We placed a stack of phantom layers of known thickness on a flat mirror, and then measured the decay of the intensity of ballistic components depending on the number of phantom layers. Specifically, we illuminated a normally incident plane wave and recorded the complex field map of the backscattered waves. We then extracted the intensity of the normally reflecting plane wave component from the recorded map. By using the exponential curve fitting, we could obtain the decay constant of 51.2µm for the roundtrip (Fig. R3). Therefore, the scattering mean free path of the scattering layer was 102.4 µm.

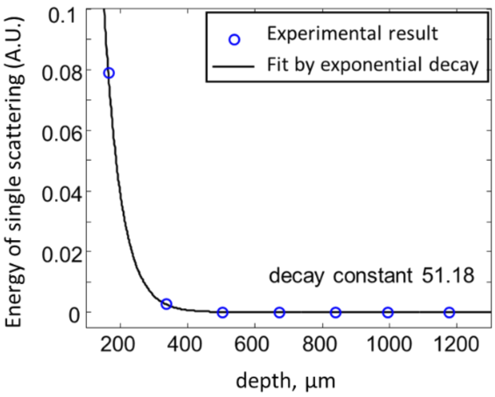

Figure R3. Calibration of scattering mean free path

In the case of rat brain tissues, scattering properties of the tissue slices vary with imaging position. Therefore, we estimated the scattering mean free path *in situ* from the total intensity of the CLASS image, which is close to the intensity of the ballistic photons, and the thickness of the tissue layer measured from the time delay. We added these details about measuring the scattering mean free path to the section III.8 of SI.

Thanks for adding this new figure in the Supplementary. Nevertheless, it would be more convenient to have Fig.R3/S17 in log-scale as an exponential fit is done here. It would show the agreement between the experimental data and the fit at large probing depths. Moreover, I have one question about this way of measuring the scattering mean free path: The backscattered intensity should also contain a multiple scattering contribution (particularly at large depths)? How do the authors discriminate single and multiple scattering in that case? The presence of multiple scattering in their data could lead to a wrong estimation of the multiple scattering intensity. I think it is important to clarify that point before publication.

With regards to the brain tissues, I don't understand exactly how the authors measure the scattering mean free path from one intensity measurement. Do they normalize this measurement by a reference intensity backscattered by a mirror in free space?

In response to the reviewer's suggestion, we presented in Fig. R3-1 the log-scale plot of the intensity of the normally reflecting plane wave as a function of the thickness of the scattering layer. Here one can notice that the first four points up to the thickness of 500 µm (1 mm in travel distance including the returning path, which corresponds to about 10 l_s) fit well to the exponential curve while the intensity taken at thicker depth was slightly higher than the fitted curve. In our measurement of the ballistic photons, we used both angular- and time-gating by the time-resolved complex field imaging. Like any other ballistic photon measurement methods, our method is effective up to a certain thickness of the sample. If the scattering medium becomes too thick, some of the multiple-scattered waves can pass through these gating operations, and this is the source of discrepancy that we observed at the thicknesses larger than 500 µm. In fact, the conventional method mostly uses the angular gating in the transmission geometry (for instance, see Ref. 12 and the work by Emily J. McDowell et al., (Journal of Biomedical Optics **15,** 025004 (2010))), and its effectiveness was shown up to about 12 l_s (supplementary material in Ref. 12), similar range to our method. However, this limitation is not

a problem at all in estimating the scattering mean free path of the phantom scattering layer. In preparing the scattering layer of an arbitrary thickness, we stacked multiple 160μm-thick scattering layers made of the mixture of polystyrene beads and PDMS solution at a uniform concentration. Reasonable measurements of ballistic photons up to the thickness of the scattering layer where the contribution of multiple scattering is negligible, which is about 500 μm in our case, will ensure the accurate estimation of the scattering mean free path of individual 160μm-thick scattering layers. And this estimation will reasonably be applied to the multiple stacked layers.

Regarding the scattering mean free path of the brain tissues, the reviewer's conjecture is correct. We took the logarithm of the intensity of the ballistic photons estimated by the CLASS image by that from the mirror for the estimation of the scattering mean free path.

We added all these discussions to the Supplementary section III.8.

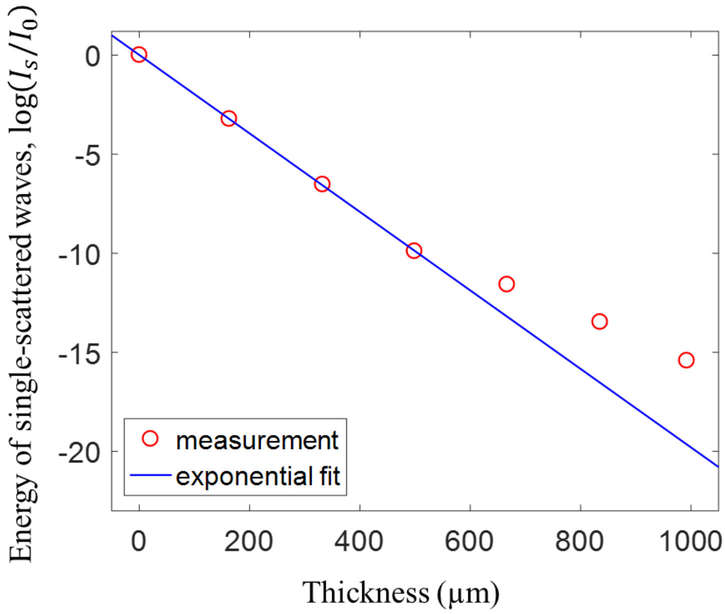

Figure R3-1. Measurement of the scattering mean free path of the scattering layers used in Fig. 1.

4. With regards to their CLASS algorithm, the authors say that they perform "a closed-loop optimization of signal waves for both forward and phase-conjugation processes. "I do not understand the interest of the phase conjugation step in their algorithm. I'm ok with the first step where they apply a correction on the reflection matrix at the input based on a conventional maximization of the single scattering intensity of the CLASS image. Then, the authors use a second step where they take the transpose conjugate of the reflection matrix (phase conjugation operation) and then apply a correction at the output still based on a maximization of the single scattering intensity. And finally iterate this process. I do not

understand here the interest of the phase conjugation operation. Without the phase conjugation operation, it should be possible to correct for aberrations, first at the input, then at the output (by just transposing the reflection matrix), and iterate the process to determine the input and output phase laws that compensate for the aberrations. Could the authors comment on that aspect? Maybe, they could show how the algorithm would work without the phase conjugation operation.

As the reviewer conjectured, the complex conjugation operation is not critical from the mathematical point of view in the second step of CLASS algorithm. It doesn't make any difference in the result except for the sign of the output phase correction map. What is important is the transpose operation, which enables us to swap input and output, and to correct the specimen-induced aberration from the output side. The reason we take phase conjugation (transpose + complex conjugation) rather than just the transpose is to impose a physical meaning to the operation. In this way, the second step is equivalent to physically sending the waves from the output side and measuring the backscattered waves at the input side.

We agree. It would be great to mention it in the paper.

As the reviewer suggested, we added this discussion to Supplementary section I.3.

5. Given the experimental set up used in their work (FigS7), the input and output arms look identical in terms of optical paths. Hence, by virtue of spatial reciprocity, the aberration phase law should be the same at the input and the output. Could the authors comment on the fact that the aberration phase laws they experimentally determine with their algorithm look quite different at the input and output (see for instance Fig4 d and e)?

As the reviewer mentioned, the aberrations for the input and output should be identical in reflection geometry due to the spatial reciprocity. In the real practice, however, the optical system in the illumination beam path from the light source to the beam splitter and that in the collection beam path from the beam splitter to the camera are not perfectly the same. In addition, there may be slight misalignment of optical axis and image focus between the two beam paths. Therefore, the input and output can have different system aberrations. But these system aberrations are relatively smaller than the specimen-induced aberrations, and the aberration maps for the input and output are largely similar. For example, the normalized cross-correlation of the complex pupil functions of the aberration maps shown in Figs. 3i and 3j was about 0.64. In the case of the correlation between the maps in Fig. 4d and 4e, their correlation was still 0.46, but a bit lower than the phantom case since the magnitude of sample-induced aberrations was smaller. Note that the coordinate systems are flipped between the input and the output, and this needs to be taken into account when they are compared.

We tried to correct aberration with the assumption that the input and output aberrations are identical. Figure R4 below shows the identified aberration map and resulting CLASS image for the sample in Fig. 4. In comparison with Fig. 4c in the main text, the resolving power and signal intensity were significantly reduced. This result shows the importance of independently addressing the input and output aberrations.

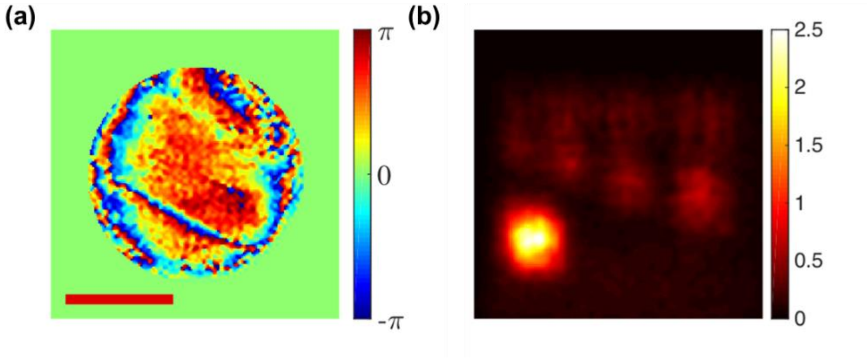

Figure R4. Aberration correction of the data in Fig. 4 with the assumption that the input and output aberrations are identical. **a,** Acquired aberration map under the assumption of identical input-output aberration. Scale bar, $k_0\alpha$. **b,** CLASS image after the aberration correction by **a.** Scale bar, 4 microns. Color bar, the intensity normalized by the maximum intensity in Fig. 4b.

We added the following sentences to the revised manuscript and these new analyses to the Supplementary section III.7.

"The input and output aberration maps are largely the same as waves travel back and forth through physically the same sample in the reflection geometry. But due to the difference in the system aberrations between the illumination beam path from the light source to the beam splitter and the collection beam path from the beam splitter to the camera, they have a slight difference (see Supplementary section III.7)."

I'm OK with the authors' explanations and changes made to the manuscript.

6. I am very skeptical with regards to the claim made by the authors in the abstract that the Strehl ratio is increased by a factor >1000 with the CLASS algorithm.

In the first experiment described in the paper, the authors say that "the signal intensity was increased in magnitude by more than 20 times" (p13, line 295). A bit further, they claim that "The initial Strehl ratio S calculated from the acquired aberration maps was 1/1300" (p14, line 310). This is highly contradictory. After correction of aberrations, the single scattering intensity should be increased by a factor $1/S^2$ (the power 2 is due to the fact that aberrations are corrected both at the input and output, see for instance the Supplementary Material of Ref.12). Hence it means that the initial Strehl ratio should be $S = 1/\sqrt{20} = 0.22$. Hence, there is here a big contradiction with their own estimation of the Strehl ratio. How do they estimate it? Details about the estimation of the Strehl ratio are not provided.

There is the same contradiction for the second experiment. The signal intensity is increased by a factor 16 (Fig.4), which yields an estimation of the Strehl ratio of 0.25 whereas the authors claim a Strehl ratio of 1/740 (p16, line 363).

At first, we clarify the definitions of S and η, and then discuss their physical meaning. In fact, the definitions of S and η, and the way they were estimated were described in full detail in the section I.6 of the previous SI (II.5 in the revised SI). In adaptive optics, S is defined by the peak intensity of the point-spread-function (PSF) in the presence of aberration relative to that without

aberration. For the case when both the input and output aberrations matter, this parameter is written as

$$S = \left|\langle \mathcal{A}(\vec{k} = 0)\rangle\right|^2 / \left|\langle \mathcal{A}_0(\vec{k} = 0)\rangle\right|^2. \qquad \text{(R1)}$$

Here $\mathcal{A} = P_i^{a*} \star P_o^a$ is the amplitude transfer function combining both input and output aberrations and $\mathcal{A}_0(\vec{k})$ is the ideal amplitude transfer function.

On the other hand, the factor η in Eq. (3) is defined by the ratio of the 'total' intensity, not the peak intensity, of the PSF in the presence of aberration with respect to that in the absence of aberration. In terms of the amplitude transfer function, η is written as

$$\eta = \langle\left|\mathcal{A}(\vec{k})\right|^2\rangle / \langle\left|\mathcal{A}_0(\vec{k})\right|^2\rangle. \qquad \text{(R2)}$$

Due to these differences in the definition, the relation $S^2 = \eta$ or $S = \eta$ that the reviewer conjectured doesn't exist. Certainly, S and η are related to a certain extent. The increase of aberration accompanies the simultaneous reduction in the peak intensity and the total intensity of the PSF. However, the peak height is more susceptible than the total intensity to the aberration. Therefore, S is usually smaller than η. But their relation depends on the type of aberration, which makes it difficult to find a general relation. For this reason, we only plotted S and η for one representative example of aberration in Fig. S6 (Fig. S7 in the revised SI).

Let us discuss the physical meaning of S and η. S is a parameter used to describe the aberration-induced signal reduction in the confocal and two-photon microscopes. It is a directly relevant parameter to the general adaptive optics audience because it indicates the degree of aberrations of the samples used in our study.

On the other hand, η describes the reduction of single-scattering intensity in the CLASS microscopy where images are acquired over wide area. Unlike confocal microscopy where signal is collected only at the confocal pinhole, the entire PSF contributes to the signal intensity in the wide-field detection. Therefore, the degradation of SNR in the CLASS microscope is determined by η, not by S. Likewise, the main role of CLASS algorithm is to increase single scattering intensity by $1/\eta$, not by $1/S$.

Now let's go back to the reviewer's comment. When we described the enhancement of signal intensity by either 20 times or 16 times, it is related to the increase of η, not S (the exact relation between 20 times and η is given below in response to reviewer's point #7). Therefore, the reviewer's estimation to deduce S from the signal enhancement is not relevant. Once again, both S and η are determined by the aberrations, but they are not equal.

First the expression of S (Eq.S15) has changed since the last version of the manuscript. It used to be:

$$S = \left|\langle \mathcal{A}(\vec{k})\rangle\right|^2 / \left|\langle \mathcal{A}_0(\vec{k})\rangle\right|^2. \text{ (T1)}$$

And now it is

$$S = \left|\langle \mathcal{A}(\vec{k} = 0)\rangle\right|^2 / \left|\langle \mathcal{A}_0(\vec{k} = 0)\rangle\right|^2. \text{(T2)}$$

According to me, there is a problem with this expression of S. The PSF and the amplitude transfer function $\mathcal{A}(\vec{k})$ are related by a Fourier transform. The peak height of the PSF (i.e the PSF at the origin) is thus proportional to $\langle\mathcal{A}(\vec{k})\rangle$. As said in the manuscript, the Strehl ratio is defined by the peak intensity of the point-spread-function (PSF) in the presence of aberration relative to that without aberration. Hence, according to me, S is given by Eq.T1 and not by Eq.T2. The authors should clarify that point. Which equation do the authors use to estimate the Strehl ratio (Eq.T1 or T2?)

Otherwise, I find the physical picture the authors provide for the relation/difference between S and η in their rebuttal letter enlightening. I think this should be included in the Supplementary Section II.5.

We appreciate the reviewer for pointing this out. There was a mistake in the mathematical expression in the revision process, and the original definition of *S*, i.e. $S = \left|\langle\mathcal{A}(\vec{k})\rangle\right|^2 / \left|\langle\mathcal{A}_0(\vec{k})\rangle\right|^2$, is a correct one. In all our estimations of *S*, this original definition of *S* was used. In the revised manuscript, we made a correction.

We are delighted that the reviewer found our discussion on S and η enlightening. We added these discussions to the revised supplementary section II.5. With the help of reviewer's comment, the descriptions of *S* and η were all made clear.

7. Related to my last point: In the Supplementary material, the authors discuss the relation between the Strehl ratio S and η, which is the ratio of the total intensity of single-scattered waves in the presence of aberration with respect to that in the absence of aberration. For the authors, S and η should be the same for moderate aberrations. According to me, the scaling should be as η = S² (see my previous point - aberrations at input and output). But, even if we consider this scaling as correct, there is still a contradiction in their experiments. For the first experiment, the increase of the single scattering intensity is of 1/ η=20 whereas they estimate 1/ S=1300. According to Fig.S6(b), for 1/ η=20, 1/S and 1/ η should be very close. The authors should absolutely clarify that point.

As we clarified in the comment #6, the relation, $S^2 = \eta$ or $S = \eta$ that the reviewer conjectured doesn't exist.

Now let us clarify η in Fig. 3. Indeed, we wrote in the main text that "Moreover, the signal intensity was increased in magnitude by more than 20 times, suggesting that the cross-correlation of the aberration-corrected pupil functions had been increased". Here the 20 times increase in signal intensity was estimated by comparing the intensity at the target in Fig. 3d and that in Fig. 3l. But this doesn't mean that η was 1/20. In fact, the real η that we estimated by applying Eq. (R2) to the measured aberration maps shown in Figs. 3i and 3j was $\eta = 1/204$ ($S = 1/1300$).

There is a discrepancy between the apparent signal enhancement of about 20 times estimated from Figs. 3d and 3l and the increase of single scattering intensity by 1/η~200. This was mainly because multiple-scattered waves as well as single-scattered waves contributed to the signal intensity at the target in Fig. 3d. Before the application of CLASS algorithm, the intensity of CLASS image at the target is the sum of single-scattered waves and the multiple-scattered waves that survived the time-gating as well as spatial coherence gating, i.e. $\mathrm{I}_{\mathrm{before}} = \mathrm{I}_{\mathrm{single}} +$

I_{multi} with initial SNR given by $I_{single}/I_{multi} = (\eta\gamma/\beta)N_m$. After the aberration correction, the intensity at the target becomes, $I_{after} = I_{single}/\eta + I_{multi}$ with SNR increased to $(\gamma/\beta)N_m$.

Since the enhancement of the apparent signal intensity at the target was about 20 times, we can set up the following equation,

$$\frac{I_{after}}{I_{before}} = \frac{I_{single}/\eta + I_{multi}}{I_{single} + I_{multi}} = 20. \quad \text{(R3)}$$

By inserting the estimated η from the measured aberration maps, we can obtain the initial single-to-multiple scattering ratio in the position basis,

$$\frac{I_{single}}{I_{multi}} = (\eta\gamma/\beta)N_m = \frac{19}{184} \simeq 0.1.$$

Therefore, multiple scattering was about 10 times stronger than the single scattering before the aberration correction, and this was responsible for the discrepancy mentioned above. Since the initial SNR of $(\eta\gamma/\beta)N_m \simeq 0.1$ was far less than unity, multiple scattering has strongly degraded the resolving power of imaging. By the aberration correction, this SNR was increased to $(\gamma/\beta)N_m \simeq 21.1$ by selectively enhancing the intensity of single-scattered waves.

Regarding the discussion on Fig. S6(b) (Fig. S7(b) in the revised SI), S and η were acquired for the numerically assumed aberrations shown in Figs. S1a and S1b in the revised SI. For these aberrations, $S = 1/3600$ at $\eta = 1/400$. In the case of experimental result shown in Fig. 3, $S = 1/1300$, and $\eta = 1/204$. Although the exact relation between S and η varies depending on the type of aberrations, the relative values of S to η in simulations and experiments are reasonably similar.

We added the following sentences to the revised manuscript to respond to all the issues raised by the reviewer. And the detailed discussion given above is added to the section III.6 of the revised SI.

"Moreover, the signal intensity at the target estimated from Fig. 3d and 3l was increased in magnitude by more than 20 times, suggesting that the cross-correlation of the aberration-corrected pupil functions had been increased."

"From the acquired angle-dependent phase correction maps in Figs. 3i and 3j, the initial Strehl ratio (see Supplementary section II.5 for the definition) was estimated to be S = 1/1300. This is two orders of magnitude smaller than the conventional adaptive optics typically handles. We also obtained $\eta = 1/204$ from Eq. (3) and measured aberration maps, which means that the total single scattering intensity was increased by about 200 times after the application of CLASS algorithm. There was a discrepancy between the apparent signal enhancement of about 20 times estimated from Figs. 3d and 3l and the increase of single scattering intensity by 200 times. And this was mainly because multiple-scattered waves as well as single-scattered waves contributed to the signal intensity at the target in Fig. 3d. Further analysis revealed that the initial single-to-multiple scattering intensity ratio was $\eta(\gamma/\beta)N_m \simeq 0.1$ (see detailed analysis for Supplementary section III. 6)."

I am convinced by the authors' reply and changes made in the manuscript and supplementary.

The previous reviewer's comment was an important stimulus for this analysis. We once again thank for the reviewer's critical comments.

8. Related to this relation between S and η, I don't understand why they should diverge at high aberration level. The explanation provided by the authors at page 11 of the supplementary material is not clear. Moreover, this in complete contradiction with Eqs.S15 and S16 that show an exactly similar expression for S and η.

As we clarified in the response to the reviewer's point #6, $S \neq \eta$ from the definitions shown in Eqs. S15 and S16. The discrepancy between S and η increases with the increase of the degree of aberration as the peak height of the PSF is more susceptible than its total intensity to the aberrations.

OK

9. Several times in the paper, the authors claim that "Typical adaptive optics can deal with aberrations for S >0.1". I think it would be relevant to provide at least one reference to corroborate that statement.

We appreciate the reviewer's suggestion, and added the following reference to the revised manuscript.

X. Tao et al., Adaptive optics confocal microscopy using direct wavefront sensing, Optics Letters **36,** 1062 (2011).

OK

10. In the last experiment shown in the paper, the authors do not discuss about the increase of the signal intensity and do not give any estimation of the Strehl ratio. Yet, in Fig.5, it seems that the signal intensity is only increased by a factor 2, which is one order of magnitude smaller than the previous experiments. Is it because there is not too much aberration in their sample or is this because the CLASS approach does not work so well in speckle (the object to image in the two first experiments is a resolution target)? This limited performance should be discussed.

The apparent signal increase at the fungal filament was about 9.05 times, not 2 times in Fig. 5h. In fact, the images were a bit saturated for better visualization of the fungal structures across the entire view field. Signal enhancement was smaller than the phantom case, but not by an order of magnitude.

As discussed above, this apparent signal increase is not equal to the enhancement of single scattering intensity η. From the obtained aberration maps of the output (Fig. 5j) and input (not shown in the figure), we obtained S=1/437 and η=1/44.7. The initial single-to-multiple scattering ratio was $(\eta\gamma/\beta)N_m \sim 0.23$ from the same analysis given to the reviewer's point #7.

For the case of the specimen in Fig. 5i, the enhancement of intensity was about 3.49 with S=1/890, η=1/68.2, respectively. With these parameters, the initial single-to-multiple scattering ratio was estimated to be about 0.04, which indicates that the biological specimens were under the influence of strong multiple scattering.

In imaging biological tissues, the single scattering intensity is inevitably smaller than the resolution target case. Therefore, the degree of multiple scattering and aberrations that can be overcome tends to be smaller. But what is important in assessing the performance of the imaging method is the initial single-to-multiple scattering ratio that the method can deal with. In this way, the performance can be characterized independent of the types of samples. In this respect, our CLASS microscope works equally well for biological tissues since we could handle initial SNR as small as 0.04 even for biological specimens.

In response to the reviewer's comment, we added S, η and initial SNR of the biological data to the revised manuscript. Also, the following discussion on the limited performance of CLASS microscope (in fact, all the coherent imaging method) for biological specimens was added to the conclusion section.

"On the other hand, CLASS microscopy requires target samples to have sufficient reflectivity for its best performance in imaging depth similar to the other coherent imaging methods working in the epi-detection geometry. In fact, the maximum possible imaging depth is determined by the reflectivity of the sample as well as the scattering properties of the scattering medium. If the reflectivity of the sample is reduced by 1/10, then the imaging depth should be reduced by 1 scattering mean free path."

I am convinced by the authors' reply and changes made to the manuscript and supplementary.

11. In their conclusion, the authors say that one important aspect is that "aberration correction is performed over a wide field of view with finely stepped illumination angles". A larger field of view also implies the presence of several isoplanatic patches in the field of view. The aberration correction provided by the CLASS algorithm is limited to a field of view that contains one single isoplanatic patch. The authors should mention it in their conclusion. It is important to mention the improvements provided by the CLASS approach but also its limits.

In our experiment, we could obtain the spatial resolution of 600 nm across the entire view field of $30 \times 30\ \mu m^2$ even at the severe aberrations shown in Fig. 3. This is mainly because we corrected aberrations for 2,800 wavevectors covering all the orthogonal modes given by the view field and numerical aperture. In fact, this is the maximum degree of correction that can be made for the given view field. As a consequence, the entire view field was seen as a single isoplanatic patch. Therefore, the CLASS algorithm is not limited to the field of view that contains one single isoplanatic patch, but enables us to treat the view field as a single isoplanatic patch by employing the maximum available degree of aberration corrections.

If the number of wavevectors is reduced by increasing the angular stepping, then the reviewer's reasoning is correct. In such case, the view field should be split into multiple isoplanatic patches, and corrections should be made to individual patches. In fact, this is the case for conventional adaptive optics. The number of modes that deformable mirrors can control is much smaller than the number of orthogonal modes set by the view field and the numerical aperture in ordinary adaptive optics. Because of this deficiency in the number of control, the area where the diffraction-limit resolution is maintained is smaller than the view field. Even in this case of reduced sampling, our CLASS microscopy has a strong benefit. After the completion of image acquisition, we can select a subimage whose area is set by the number of measured orthogonal modes, and then apply CLASS algorithm for the selected subimage. By performing this subimage correction for multiple isoplanatic patches in the original wide area image, we can

ensure diffraction-limit spatial resolution over the entire view field. Indeed, the post-processing capability of CLASS microscopy works as a merit in this case.

In response to the reviewer's comment, we added the following sentence to the discussion section of the revised manuscript.

"In addition, due to the aberration correction for all the orthogonal modes within the view field, diffraction-limit spatial resolution can be obtained across the entire view field."

I'm not sure to fully agree with the authors: the high spatial frequencies of an aberrating layer are associated with a smaller isoplanatic patch. See for instance J. Mertz, H. Paudel, and T. G. Bifano, Appl. opt. 54, 3498, 2015 where it is shown that the size of one isoplanatic patch scales with the characteristic length scale of the phase variations induced by the aberrating layer. Reducing the angular stepping thus increases the size of the isoplanatic patches. Anyway, the field of view considered in that paper is relatively reduced, so this problem is probably out-of-the-scope of that paper. I was just curious about the way they could face this issue.

The size of the isoplanatic patch has been a subtle issue as it is affected by many factors including the spatial steepness of aberrations, the thickness of scattering media, target diffraction-limit spatial resolution and the specifications of spatial light modulators in their number of pixels and numerical aperture coverage. There can be diverse viewpoints such as the one presented in the work J. Mertz et al, but here we present the interpretation directly relevant to our system.
At first, we need to understand why the isoplanatic patch is much smaller than the field of view in the conventional adaptive optics where spatial light modulator is typically located at the pupil plane. This is mainly because each SLM pixel covering the spatial frequency resolution Δk controls not only the ballistic single-scattered waves but also the snake photons for the aberration correction. The control of snake photons is beneficial in terms of the excitation intensity, but we need to consider its downside. The point-spread-function (PSF) of the ballistic photons gets spread due to the angle-dependent phase retardation by the sample. And the way the snake photons get spread in their PSF is different from that of the ballistic photons. With a single SLM, it is impossible to correct both ballistic and snake photons at the same time. In fact, the correction is made to their averaged field at the SLM pixel. More importantly, the snake photons are position-dependent (or spatially variant), thereby degrading the effectiveness of aberration correction at positions other than the corrected spot. This explains why the isoplanatic patch becomes smaller as aberrations get steeper and the Δk of the SLM is larger, where the contribution of snake photons increases.
The uniqueness of our approach is that the CLASS algorithm exclusively controls ballistic photons, not the snake-like or multiple-scattered photons, by enhancing the coherent summation of single-scattered waves over wide view field (see Supplementary section II.4). Considering that the single-scattered waves are spatially invariant, our correction can be effective over the entire view field so long as all the orthogonal free modes are covered in the matrix measurements. In this respect, the entire field of view could be considered a single isoplanatic patch in our experiment. For reducing the number of measurements, we can increase the angular stepping size of illumination, or equivalently the Δk. In such case, the patch size will be reduced to $1/\Delta k$.

Since we could obtain the diffraction-limit resolution over the entire view field in the present study and the size of the isoplanatic patch is not the main scope of this study, we think that it would be better to leave this issue for further investigation in our future study.

Reviewer #2

The authors addressed most of the points that I raised during my first response. However, some additional issues raised by Reviewer #1 are valid and the authors' responses to those have created concerns on which I also comment below:

We appreciate the reviewer for acknowledging our previous response, and also for providing us with additional concerns and comments. We made efforts to address each concern to the best of our knowledge, and hope that it would be satisfactory.

Regarding my question about the difference between the phase maps of the input and output aberrations (as also raised by Reviewer #1 in point 5) and Figure R5, it seems that the total difference between the two phase maps is dominated by spherical system aberrations. In that case, this isn't the best example to demonstrate the technique, unless the authors only want to claim the ability to correct system aberrations.

We think that there is slight misunderstanding in the reviewer's comment. Upon the request by the reviewer, we presented the difference between the input and output aberration maps in Fig. R5. In the original input and output aberration maps, we already identified the sample-induced aberrations as well as the system aberration. The difference between the two maps was shown only to emphasize the difference in the system aberration between the illumination and collection beam paths. Once again, we would like stress that the sample-induced aberrations were identified and corrected in the present study, let alone the system aberrations.

I think that there is some confusion introduced by the use of the term "single scattered waves" vs "multiple scattered waves" as used by the authors. By the term "single scattered waves" the authors mean the ballistic back-scattered waves from the target and not the waves that have been involved in any scattering events inside the medium. In the CASS technique, the authors accumulate this ballistic light and suppress the contribution of sample induced scattered light. Therefore, for the authors, any light that is not ballistic back-scattered by the object is "multiply scattered light" which I believe is misleading. Reviewer #1 raised an interesting point about the extent of the diagonal elements in real space. From that one can see that scattering seems to be dominated by "sample inhomogeneity single/few scattering events" and not by multiple scattering of light (elements close to the main diagonal), since multiple scattered light will be outside the time gate (due to a longer optical path inside the medium). Furthermore, the single to multiple scattered light ratio would be more correctly called ballistic to scattered light ratio and should not be confused with multiple scattering events.

To address the reviewer's point, we feel that it is necessary to reiterate the overall experimental procedure. In fact, we used a time-gated detection to filter out those multiple-scattered waves having different flight time from single-scattered waves in the first place (see Supplementary section 4 of Ref. 10 for the effect of time gating). We then applied CLASS algorithm to preferably accumulate single-scattered waves over the residual multiple-scattered waves. As the reviewer conjectured, the residual multiple-scattered waves are mostly due to a few scattering events and distributed around the diagonal in the time-gated reflection matrix as they were already time-gated. Therefore, it is natural for us to compare single-scattered waves with these residual multiple-scattered waves in assessing the performance of CLASS algorithm.
Regarding the reviewer's comment on the terminology, we are not sure why these residual multiple-scattered waves should not be called multiple-scattered waves. To our picture, the detected backscattered waves can be categorized into single- and multiple-scattered waves. And

the multiple-scattered waves may further be divided into a-few-times scattered waves and strongly scattered waves depending on the number of scattering events. The residual multiple-scattered waves may not be due to 'strong' multiple scattering, but certainly by the scattering events more than once. In this respect, we think that the use of single- to multiple-scattering ratio is valid.
In our demonstration, the thickness of the scattering layer was about $7l_s$, and we described this condition as a 'strong' multiple scattering regime in our original manuscript. In the revised manuscript, we dropped the adjective 'strong' as its definition varies from one literature to another.

The response of the authors to point #11 of Reviewer #1 is not clear and also raises a number of issues. My thoughts on the issue:
a. The number of wave vectors used to create the CASS image defines the maximum number of independent modes at the pupil plane that can be measured and is not related to the isoplanatic patch.
b. Based on the experimental setup and the way that the problem is formulated, the sample induced aberrations are treated as a single surface phase aberration layer that affects the incoming and outgoing waves all at the same time. As already discussed, those should be the complex transpose of one another apart from system aberrations that might be different of the input and output arms. Therefore, the sample aberrations that are corrected form indeed a single isoplanatic patch.
c. The treatment of different isoplanatic patches would require the independent measurement of smaller spatially separated fields of view and the corresponding generation of different correction phase maps.

The size of the isoplanatic patch has been a subtle issue as it is affected by many factors including the spatial steepness of aberrations, the thickness of scattering media, target diffraction-limit spatial resolution and the specifications of spatial light modulators in their number of pixels and numerical aperture coverage. There can be diverse viewpoints, but here we present the interpretation relevant to our system.
At first, we need to understand why the isoplanatic patch is much smaller than the field of view in the conventional adaptive optics where spatial light modulator is typically located at the pupil plane. This is mainly because each SLM pixel covering the spatial frequency resolution Δk controls not only the ballistic single-scattered waves but also the snake photons for the aberration correction. The control of snake photons is beneficial in terms of the excitation energy, but it has its downside. The point-spread-function (PSF) of the ballistic photons gets spread due to the angle-dependent phase retardation by the sample. And the way the snake photons get spread in their PSF is different from that of the ballistic photons. With a single SLM, it is impossible to correct both ballistic and snake photons at the same time. In fact, the correction is made to their averaged field at the SLM pixel. More importantly, the snake photons are position-dependent (or spatially variant), thereby degrading the effectiveness of aberration correction at positions other than the corrected spot. This explains why the isoplanatic patch becomes smaller as aberrations get steeper and the Δk of the SLM is larger, where the contribution of snake photons increases.
The uniqueness of our approach is that the CLASS algorithm exclusively controls ballistic photons, not the snake-like or multiple-scattered photons, by enhancing the coherent summation of single-scattered waves over wide view field (see Supplementary section II.4). Considering that the single-scattered waves are spatially invariant, our correction can be effective over the entire view field so long as all the orthogonal free modes are covered in the matrix measurements. In this respect, the entire field of view could be considered a single isoplanatic

patch in our experiment. For reducing the number of measurements, we can increase the angular stepping size of illumination, or equivalently the Δk. In such case, the patch size will be reduced to 1/Δk in our case.

Since we could obtain the diffraction-limit resolution over the entire view field in the present study and the size of the isoplanatic patch is not the main scope of this study, we think that it would be better to leave this issue of isoplanatic patch for further investigation in our future study.

Very importantly, motivated by the points raised by Reviewer #1 regarding the Strehl ratio, my opinion is that the use of the Strehl ratio for the quantification of the aberrations in the current context is problematic and could easily lead to confusions. The authors have estimated the Strehl ratio from Equation R1 (also present in the Supplementary Material), which defines it as the ratio between the squared amplitude of the summed crosscorrelation between the input and output aberrations over the equivalent measure for a uniform pupil. Such a metric is equivalent to comparing the expected intensity at the center of the aberrated PSF against the intensity at the center of an ideal diffraction-limited PSF. Although this definition can be found in the literature, I think it could lead to an overestimation of the actual aberrations. Conventionally, when the ground-truth is not known, the Strehl ratio should be calculated by removing piston, tilt and defocus terms, since those are trivially corrected by the optical setup and do not lead to image degradation relative to the ideal diffraction limited PSF. Even further, the Strehl ratio was conceived to quantify the deterioration of the image quality of an imaging system under the presence of aberrations. Since this metric is indeed what matters, I think that only the image intensity enhancement factors are the ones that reflect important information and are not misleading.

In addition to the intensity enhancement factor η, we think that the Strehl ratio is a relevant reference parameter to the general audience as it has been widely used for describing the degree of sample-induced aberration. As the reviewer mentioned, we might have followed the definition in the literature too strictly in our estimation of the Strehl ratio. In response to the reviewer's suggestion, we estimated it again after removing the piston, tilt and defocus terms. The new value is now 1/531 (original value was 1/1300) for the aberrations shown in Fig 3. Strehl ratio for the other samples were all estimated again and the values in the main text were revised accordingly. The enhancement in Strehl ratio may seem unusually high, but it is in the reasonable range considering that we used 2,800 angular elements for the aberration correction.

Revisions were made at the abstract and main text.

In abstract,

"We demonstrated the enhancement of Strehl ratio by more than 500 times, an order of magnitude or more improvement over conventional adaptive optics,"

In main text,

"However, the degree of aberrations that we dealt with in the present study was so severe that S is around 1/500 (piston, tilt, and defocus terms were removed in our estimation of Strehl ratio following the convention in adaptive optics), more than an order of magnitude smaller than that the conventional adaptive optics can handle."

REVIEWERS' COMMENTS:

Reviewer #1 (Remarks to the Author):

I'm convinced by the response of the authors and the corrections made on the paper. I suggest to publish the paper as it is.

Reviewer #2 (Remarks to the Author):

The authors addressed all my questions.
